# Phenotypic-dependent variability and the emergence of tolerance in bacterial populations

**DOI:** 10.1371/journal.pcbi.1009417

**Published:** 2021-09-23

**Authors:** José Camacho Mateu, Matteo Sireci, Miguel A. Muñoz

**Affiliations:** 1 Departamento de Matemáticas, Universidad Carlos III de Madrid, Leganés, Spain; 2 Departamento de Electromagnetismo y Física de la Materia and Instituto Carlos I de Física Teórica y Computacional, Universidad de Granada, Granada, Spain; University of Zurich, SWITZERLAND

## Abstract

Ecological and evolutionary dynamics have been historically regarded as unfolding at broadly separated timescales. However, these two types of processes are nowadays well-documented to intersperse much more tightly than traditionally assumed, especially in communities of microorganisms. Advancing the development of mathematical and computational approaches to shed novel light onto eco-evolutionary problems is a challenge of utmost relevance. With this motivation in mind, here we scrutinize recent experimental results showing evidence of rapid evolution of tolerance by lag in bacterial populations that are periodically exposed to antibiotic stress in laboratory conditions. In particular, the distribution of single-cell lag times—i.e., the times that individual bacteria from the community remain in a dormant state to cope with stress—evolves its average value to approximately fit the antibiotic-exposure time. Moreover, the distribution develops right-skewed heavy tails, revealing the presence of individuals with anomalously large lag times. Here, we develop a parsimonious individual-based model mimicking the actual demographic processes of the experimental setup. Individuals are characterized by a single phenotypic trait: their intrinsic lag time, which is transmitted with variation to the progeny. The model—in a version in which the amplitude of phenotypic variations grows with the parent’s lag time—is able to reproduce quite well the key empirical observations. Furthermore, we develop a general mathematical framework allowing us to describe with good accuracy the properties of the stochastic model by means of a macroscopic equation, which generalizes the Crow-Kimura equation in population genetics. Even if the model does not account for all the biological mechanisms (e.g., genetic changes) in a detailed way—i.e., it is a phenomenological one—it sheds light onto the eco-evolutionary dynamics of the problem and can be helpful to design strategies to hinder the emergence of tolerance in bacterial communities. From a broader perspective, this work represents a benchmark for the mathematical framework designed to tackle much more general eco-evolutionary problems, thus paving the road to further research avenues.

## Author summary

Problems in which ecological and evolutionary changes occur at similar timescales and feedback into each other are ubiquitous and of outmost importance, especially in microbiology. A particularly relevant problem is that of the emergence of tolerance to antibiotics by lag, that has been recently shown to emerge very fast in bacterial (E. coli) populations under controlled laboratory conditions. Here, we present a computational individual-based model, allowing us to reproduce empirical observations and, also, introduce a very general analytical framework to rationalize such results. We believe that our combined computational and analytical approach may inform the development of well-informed strategies to mitigate the emergence of bacterial tolerance and resistance to antibiotics and, more generally, can help shedding light onto more general eco-evolutionary problems.

## Introduction

The extraordinary ability of species to adapt and survive in unpredictably-changing and unfavorable environments is certainly one of the most astonishing features among the many wonders of the phenomenon that we call life. Such adaptations can occur at extremely fast temporal scales thus interspersing ecological and evolutionary processes [1–3]. A widely spread surviving strategy is *latency* or *dormancy*, i.e., the possibility for organisms to enter a period of reduced metabolic activity and non-replication adopted during adverse environmental conditions [4–8]. Examples of dormancy can be found across kingdoms, with examples ranging from microorganisms such as viruses, bacteria or fungi [9–12] to plants [13, 14] and animals [7]. During the latency period the organism is said to be in a *latent or dormant state* and the time it takes to wake up is referred to as “lag time” or simply “lag”. Entering and exiting a dormant state are not cost-free processes, since individuals may require of a specific metabolic machinery for performing such transitions and/or the development of specifically-devised “resting structures” [4, 5, 15–17]. The exit from the dormant state can occur either as a response to environmental signals or cues [4, 5, 10, 18] or, alternatively, in a stochastic way [19–23]. As a matter of fact, the duration of the lag intervals often varies widely between conspecific individuals and even between genetically identical organisms exposed to the very same environmental conditions [10, 19, 24, 25]. Such a variability is retained as an example of *phenotypic diversification* or *bet-hedging strategy* [26, 27] that confers a crucial competitive advantage in unpredictable and rapidly changing environments, thus compensating the above-mentioned individual costs and providing important benefits to the community as a whole [4, 5, 10, 14, 28, 29].

Although, as already stated, latency is a widespread phenomenon, bacterial communities constitute the most suitable playground for quantitative analysis of latency owing to their diversity, fast life cycle, and the well-controlled conditions in which they can grow and proliferate in the laboratory [30–33]. Actually, latency was first described by Müller back in 1895 as an explanation for the observed irregularities in the growth rate of bacterial cultures in his laboratory [34]. In recent years it has been realized that bacterial latency is a more complex and rich phenomenon than previously thought. Indeed, paraphrasing a recent review on the subject, the lag phase is “dynamic, organized, adaptive, and evolvable” [10].

Bacterial latency is at the root of *tolerance* to antibiotics as, rather often, bactericidal antibiotics act during the reproduction stage and thus, by entering a dormant state, bacteria become transiently insensitive to antibiotics. Let us recall that bacterial *tolerance* is not to be confused with bacterial *resistance* [35]. While *resistance* refers to the ability of organisms to grow within a medium with antibiotics, provided these are not in high concentrations, *tolerance* is the ability to transiently overcome antibiotics, even at very high concentrations, provided the exposition time is not too large [24, 35, 36]. The strengths of these two complementary surviving strategies are quantified, respectively, in terms of quantities: **(i)** the *minimum inhibitory concentration* (MIC) of drug that must be supplied to stop the population growth—a quantity that is significantly increased in resistant strains [24, 25, 35, 37]—and **(ii)** the *minimum duration to kill 99% of the cells MDK*_99_, which is increased in tolerant strains [38].

While the importance of bacterial resistance has long been recognized, studies underlining the crucial role played by tolerance are less frequent and more recent [24, 25, 35, 37]. An important caveat is that, while resistance is specific to one or a few antibiotics, tolerance is generically effective for a large diversity of them, leading to survival even under intensive multidrug treatment [24, 25, 35]. Moreover, there exists firm evidence that tolerance is the first response to antibiotic stress [37], facilitating the later appearance of resistance [25]. Therefore, understanding the emergence of tolerance is crucial for the development of more effective therapies aimed at dealing with recalcitrant infections and possibly preventing them. Aimed at shedding light on these issues, here we present an eco-evolutionary approach to analyze the emergence of tolerance by lag in bacterial communities under controlled laboratory experiments. In particular, we scrutinize the conditions under which modified lag-time distributions evolve as a response to stressful environments and investigate the origin of the experimentally-observed broad heavy tails in lag-time distributions (see below).

Beside this specific focus, the present work has a broader breath. The example of rapid evolution of lag-time distributions is used as a test to prove a theoretical framework that we are presently developing. Our framework is similar in spirit to existing approaches such as the theory of *“adaptive dynamics”* and related models in population genetics [39–42], but aims at reconciling and generalizing them.

As a historical sidenote, let us recall that *adaptive dynamics* (AD) was born as a generalization of evolutionary game theory [43] to allow for a set of strategies that is continuously varying and, upon which selection acts. AD led to the satisfactory explanation of intriguing phenomena such as evolutionary branching [39, 40, 44, 45], speciation [46–48], diversification [49, 50], the emergence of altruism and cooperation [51, 52], and the evolution of dispersal [53]. Importantly, its foundations are also mathematically well-established [54]. However, in spite of its very successful history, AD in its standard formulation has some limitations that make it not directly applicable to complex situations such as the one we aim at describing here:

1. First of all, in its standard formulation, populations are considered as monomorphic, i.e. point-like in phenotypic space; thus it does not allow for phenotypically-structured populations (see however [55, 56]).
2. The “macroscopic equations” of AD for the populations are not easily connected to microscopic birth-death processes in individual-based models [57].
3. Variations are assumed to be small, typically Gaussian-distributed and independent of the parent’s phenotypic state.
4. Variations are considered to be rare: “after every mutational event, the ecological dynamics has time to equilibrate and reach a new ecological attractor” [58]. In other words, a separation is assumed between ecological and evolutionary timescales, while in microbial communities, such processes may occur in concomitance. Such a convergence of characteristic timescales is the hallmark of eco-evolutionary dynamics [1–3] and is at the basis of fascinating phenomena such as eco-evolutionary tunneling [58, 59] and other rapid evolutionary phenomena [32, 60–63] which are difficult to account for in the standard formulation of adaptive dynamics.

In what follows, we employ a theoretical framework in the spirit of statistical mechanics that aims to fill the gap between theory, phenomenological models and, most importantly, experiments. Thus, our approach—which is similar in spirit to previous work on bacterial quorum-sensing by E. Frey and collaborators [64, 65]—implements a number of extensions with respect to standard AD [66], as it makes explicit the connection between individuals (microscale), and community dynamics (macroscale), introduces a general variation kernel, allows for large and phenotypic-state-dependent variations, etc. These extensions allow us to study phenotypic diversity within a well-characterized eco-evolutionary framework. A full account of this general theoretical framework will be presented elsewhere [67]. Let us finally emphasize that many of the above questions and extensions have been already tackled in the mathematical literature, at a formal level [54, 68]. Yet, to the best of our knowledge, these results have been confined to rigorous analyses of toy models and have not fully percolated through the biological and physical literature.

The paper is organized as follows: in the first section we discuss in detail the experimental setup and empirical findings object of our study; then, we introduce a stochastic individual-based model implementing phenotypic variability and inheritability to account for experimental results. We present an extensive set of both computational and analytical results for it, discussing in particular the conditions under which the mathematical results deviate from computational ones. Finally, we discuss the implications of our work both from a biological viewpoint and how it contributes to the understanding of the evolution of heterogeneous phenotypic distributions, as well as from a more general eco-evolutionary perspective.

### Empirical observations: Rapid evolution of lag-time distributions

For the sake of concreteness, we focus on recent experimental results on the rapid evolution of tolerance in populations of *Escherichia coli* in laboratory batch cultures in Balaban’s lab [24]. In particular, a bacterial population is periodically exposed to antibiotics (*amplicillin*) in very high concentrations (much larger than the *MIC*) during a fixed-duration time interval *T*_*a*_ (e.g., *T*_*a*_ = 3, 5, or 8 hours). After antibiotic exposure the system is washed and the surviving population is regrown in a fresh medium during a time interval *T* (with *T* = 23*h* − *T*_*a*_). The antibiotics/fresh-medium cycle is iterated at least 8 or 10 times. Results are averaged over 2 experimental realizations for each *T*_*a*_ and the resulting maximal carrying capacity is about 10^9^ individuals (we refer to [24] for further biological and experimental details).

Once the cycles are completed, Fridman et al. [24] isolated some individuals from the surviving community and by regrowing them in a fresh medium they found that the distribution *P*(*τ*) of lag times *τ*—i.e. the time individual dormant cells take to start generating a new colony after innoculation into a fresh medium—changes from its ancestral shape to a modified one, shifted towards larger *τ* values. More specifically, the mean value grew to a value that approximately matches the duration of the antibiotic-exposure time interval, *T*_*a*_ (see [24]). This modified lag-time distribution entails an increase in the survival probability under exposure to ampicillin but, also, to antibiotics of a different bactericidal class such as *norfloxacin*, for the same time period. Furthermore, mutations were identified in diverse genes, some of them known to be related with regulatory circuits controlling the lag-time distribution, such as the toxin–antitoxin one [69]. Subsequently, after many cycles, the population was also observed to develop resistance to ampicillin [24]. Thus, the conclusion is that non-specific tolerance—stemming from lag—emerges in a very rapid way as a first adaptive change/response to antibiotic stress. More in general, these results reveal that the adaptive process is so fast that ecological and evolutionary processes occur at comparable timescales [63, 70–73].

The experimentally-determined lag-time distributions reveal another intriguing aspect that—to the best of our knowledge—has not been extensively analyzed so far: their variance is also significantly increased as *T*_*a*_ grows and, related to this, the resulting mean value of the distribution is always larger than its median [74]. This is an indication that, as a matter of fact, the empirically-obtained lag-time distributions are skewed and exhibit heavy tails, including phenotypes with anomalously-large lag times—much larger than *T*_*a*_—, especially for large *T*_*a*_’s. This observation is surprising as, under such controlled lab conditions, one could naively expect to find lag-time distributions sharply peaked around the optimal time value, *T*_*a*_, since, ideally, the best possible strategy would be to wake-up right after antibiotics are removed and any further delay comes at the price of a reduction of the overall growth rate or fitness. Fridman *et al*. proposed that the increase in the variance might suggest a past selection for a bet-hedging strategy in natural unpredictable environments; however, anomalously-large lag-time values were not present in the original wild-type population. The authors also suggested that there could be constraints at the molecular level imposing the mean and the variance of the lag-time distribution to increase concomitantly [24, 69], a possibility that inspired us and that we will carefully scrutinize from a theoretical and computational perspective in what follows.

## Model building

**Fig 1:**
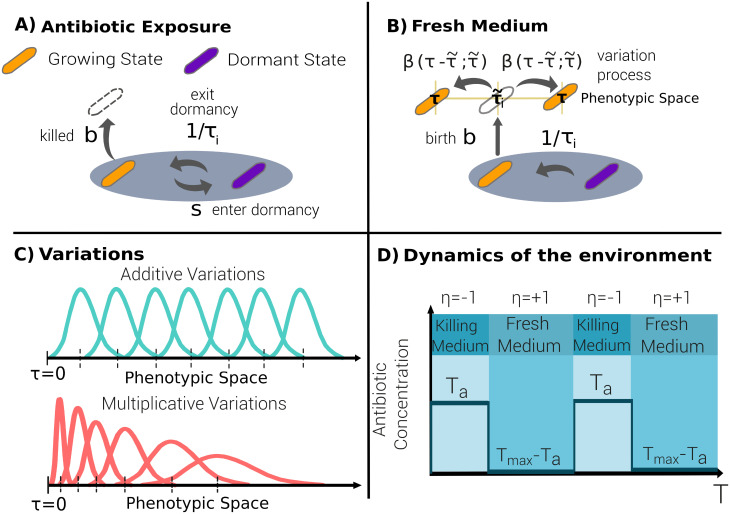
Sketch of the main ingredients of the individual-based stochastic model. Each individual bacterium (*i*) is characterized by its phenotypic state, lag time *τ*_*i*_ and experiences demographic processes. **(A)** In the presence of antibiotics, bacteria can stochastically switch between the dormant and the growing state (at transition rates *s* and 1/*τ*_*i*_, respectively); growing individuals can also attempt reproduction (at a “birth” rate *b*) and be immediately killed by the action of antibiotics (as bactericidal antibiotics usually act during duplication attempts). **(B)** In the fresh medium, dormant bacteria can wake up at a rate 1/*τ*_*i*_, that depends on their intrinsic (phenotypic) lag time; on the other hand, growing cells can reproduce asexually by duplication; the resulting offspring inherit the characteristic time scale with some variation, as specified by a function *β*. **(C)** Two possible types of variation functions *β*: in the additive case (top), the standard deviation is constant, i.e. independent of the initial state *τ*_*i*_, while in the multiplicative case (bottom) the standard deviation is assumed to grow linearly with the parent’s lag time *τ*_*i*_. **(D)** Sketch of the environmental variation, alternating periodically between antibiotic exposure (time *T*_*a*_) and a fresh medium (*T*_*max*_ − *T*_*a*_).

Aimed at shedding light onto these empirical findings, here we propose an individual-based stochastic model for phenotypic adaptation in which each single individual cell can be either in an “awake” or in a “dormant” state [22, 23, 75–77] (see Fig 1 for a sketch of the model). Mimicking the experimental protocol of Fridman et al.—a population of such individuals is exposed to alternating adverse and favorable conditions with durations *T*_*a*_ and 23*h* − *T*_*a*_, respectively (a function *η*(*t*) labels the environmental state at any given time *t*: *η*(*t*) = −1 in the presence of antibiotics and *η*(*t*) = +1 in the fresh medium).

The model assumes that each awake cell is able to sense the environment and respond to it by regulating its state: they can sense the presence of antibiotics and enter the dormant state at rate *s*, while such a machinery is assumed to be turned off during dormancy. In S1 Text, Sec. S5, we also consider a generalization of the model in which awake individuals can also enter the dormant state as a response to other sources of stress such as starvation [78]. Indeed, the wake-up is assumed to occur as a result of a Markovian stochastic process; each individual bacteria *i* is *phenotypically* characterized by its intrinsic typical mean lag time *τ*_*i*_ meaning that, it wakes up stochastically at a constant transition rate 1/*τ*_*i*_. Therefore, the time *t* at which a dormant cell actually resumes growth is a random variable distributed as $P\left(t|\tau_{i}\right)=e^{-t/\tau_{i}}/\tau_{i}$, with mean value *τ*_*i*_ [79, 80]. In the last section we discuss recent alternatives to Markovian processes, i.e. including some form of “memory”, which can give rise to non-exponential residence times, to describe this type of waking-up phenomena [81, 82].

Awake individuals are exposed to stochastic demographic processes: they attempt asexual reproduction (i.e., duplication) at a constant birth rate *b* and die spontaneously at rate *d* (that we fix to 0 without loss of generality). Reproduction attempts are successful in the fresh medium while, in the presence of antibiotics, they just lead to the parent’s death and its removal from the community. Following this dynamics, the population can freely grow, until its size reaches a maximal carrying capacity *K*. Once this limit has been reached, the population enters a saturated regime, within which each new birth is immediately compensated by a random killing (much as in the Moran process [83]).

Importantly, in parallel with the above demographic processes, the model implements an evolutionary/adaptive dynamics. The phenotypic state *τ*_*i*_ of each successfully dividing individual is transmitted, with possible variation, to its progeny. In particular, the two offspring resulting from duplication have phenotypic states *τ*_*i*_ + *ξ*_1_ and *τ*_*i*_ + *ξ*_2_, respectively, where *ξ*_1_ and *ξ*_2_ are the phenotypic stochastic variations, sampled from some probability distribution, that we generically call *β*(*ξ*; *τ*_*i*_) and that, in the more general case, can be state-dependent, i.e. depend on *τ*_*i*_. More specifically, we implemented two different variants of the model, depending of the standard deviation of the probability distribution *β*(*ξ*; *τ*_*i*_):

- The *additive* model, with a standard deviation, *α*_*A*_, common to all phenotypes.
- The *multiplicative* model, with a state-dependent standard deviation, *α*_*M*_ *τ*_*i*_, for individuals with intrinsic lag time *τ*_*i*_, where *α*_*M*_ is a constant (see Methods).

Observe that in the multiplicative case, the larger the parent’s lag time the larger the possible amplitude of variations, in a sort of rich-get-richer or Matthew-effect mechanism, well-known in the theory of stochastic processes to generate heavy tails [84–93]. As a motivation for this choice, let us mention that there is solid evidence that the genetic circuits involved in the regulation of the lag-time distribution (such as the toxin-antitoxin one), can indirectly produce this type of fluctuations at the phenotypic level [69]. Furthermore, similar phenotypic-variation kernels have been argued to arise from non-linear effects in the way genotypic changes (mutations) are manifested into phenotypic variability (see e.g. [94, 95]).

## Analytical (mean-field) theory

Before delving into computational analyses of the model, let us present a mathematical framework allowing us to obtain theoretical insight. Readers not particularly interested in analytical approaches can safely skip this section, and just be aware that it is possible to mathematically understand all the forthcoming computational results.

The previous Markovian stochastic individual-based model is mathematically defined as a “many-particle” Master equation ruling the time evolution of the joint probability-distribution functions for the whole set of all “particles” (i.e., cells). The resulting master equation can be simulated computationally by employing the Gillespie Algorithm (see below and S1 Text, Sec. S1B, for details) [79, 80, 96]. However, as it is often the case for such many-particle Master equations, it is hard to handle analytically in an exact way. Thus, in order to gain quantitative understanding beyond purely computational analyses, here we develop an approximation—which becomes exact in the limit of infinitely large population sizes [64, 65]—that allows us to derive a macroscopic (or “mean-field”) description of the stochastic model in terms of the probability density of finding an individual at any given phenotypic state, *τ* (i.e. the “one-particle” probability density). The mean-field approach that we employ in what follows is just a first example of a much more general framework that we will expose in detail elsewhere [67].

A first step toward the derivation of a *macroscopic equation* relies on a marginalization of the many-particle probability-distribution function to obtain a one-particle probability density (see S1 Text, Sec. S2A). The resulting marginalized distribution function encapsulates the probability density *ϕ*(*τ*, *t*) that a randomly sampled individual at time *t* has lag time *τ*. This probability—that needs to be normalized, so that $\int_{0}^{\infty }\phi \left(\tau ,t\right)d\tau =1$—can be decomposed in two contributions *ϕ*(*τ*, *t*) = *ϕ*_*G*_(*τ*, *t*) + *ϕ*_*D*_(*τ*, *t*) representing, respectively, the relative fraction of individuals in growing (G) and dormant (D) states. Observe that these two densities are not probability distributions and thus they are not normalized to unity separately. In the limit of infinitely-large population sizes, the evolution of the probability density for individuals in the growing state, *ϕ*_*G*_(*τ*, *t*) is ruled by the following equation (details of the derivation can be found in S1 Text, Sec S2B):

$$\begin{array}{ccc} \partial_{t}\phi_{G}\left(\tau ,t\right) & = & \frac{1+\eta (t)}{2}[-b\phi_{G}\left(\tau ,t\right)+2b\int_{0}^{\infty }d\tilde{\tau }\beta \left(\tau -\tilde{\tau };\tilde{\tau }\right)\phi_{G}\left(\tilde{\tau },t\right)\\ & - & b\phi_{G}\left(\tau ,t\right)\int_{0}^{\infty }d\tilde{\tau }\phi_{G}\left(\tilde{\tau },t\right)]\\ & - & \frac{1-\eta (t)}{2}[b(1-\int_{0}^{\infty }d\tilde{\tau }\phi_{G}\left(\tilde{\tau },t\right))+s]\phi_{G}\left(\tau ,t\right)+\frac{1}{\tau }\phi_{D}\left(\tau ,t\right). \end{array}$$ (1)

Even if this equation might look cumbersome, its different terms have a rather intuitive interpretation:

- *In the fresh medium* (terms proportional to 1 + *η*(*t*)): (i) the first term represents the negative probability flow stemming from growing individuals with generic phenotypic trait *τ* that reproduce (at rate *b*) and change to any other arbitrary phenotypic state; (ii) the second represents the positive contribution of reproducing individuals (at rate *b*) with any arbitrary trait $\tilde{\tau }$, for which one of the two resulting offspring jumps to *τ* (controlled by the function $\beta (\tau -\tilde{\tau };\tilde{\tau })$); (iii) the third *selection term* stems from the normalization of the overall probability density: if the population size grows because any individual with arbitrary trait $\tilde{\tau }$ reproduces successfully (at rate *b*), then the relative probability to observe phenotype *τ* decreases to keep the overall probability-density conserved.
- *In the presence of antibiotics* (terms proportional to 1 − *η*(*t*)): (i) the first term represents the rate at which growing individuals that attempt reproduction (at rate *b*) are killed by antibiotics; (ii) the second term is a selection term, fully analogous to the above-discussed one: when any arbitrary individual dies the overall probability density at *τ* increases; (iii) the third term represents the outflow of individuals entering the dormant state at rate *s*.
- *In both environments* (no dependence on *η*(*t*)): the only term, proportional to the rate 1/*τ*, describes the probability inflow stemming from dormant individuals that become awake.

Similarly, the equation for the density of individuals in the dormant state is

$$\begin{array}{ccc} \partial_{t}\phi_{D}\left(\tau ,t\right) & = & -\eta \left(t\right)b\phi_{D}\left(\tau ,t\right)\int_{0}^{\infty }d\tilde{\tau }\phi_{G}\left(\tilde{\tau },t\right)-\frac{1}{\tau }\phi_{D}\left(\tau ,t\right)+\frac{1-\eta (t)}{2}s\phi_{G}\left(\tau ,t\right) \end{array}$$ (2)

where the first (selection) term stems from the overall probability conservation when the population either grows or shrinks (negative or positive signs, respectively), and the remaining two terms have the opposite meaning (and signs) of their respective counterparts in Eq (1).

In order to make further analytical progress, in the case in which variations are assumed to be small, it is possible to introduce a further (“diffusive” or “Kimura”) approximation as often done in population genetics as well as in adaptive or evolutionary mathematical approaches [97]. More specifically, one can perform a standard (Kramers-Moyal) expansion of the master equation by assuming that jumps in the phenotypic space are relatively small [79, 80], i.e. expanding the function *beta* in Taylor series around 0. After some simple algebra (see S1 Text, Sec. S3) one obtains a particularly simple expression for the overall probability distribution:

$$\begin{array}{ccc} \partial_{t}\phi \left(\tau ,t\right) & = & \eta \left(t\right)[f\left(\tau ,t\right)-\bar{f}\left(t\right)]\phi \left(\tau ,t\right)\\ & - & \left(\eta \left(t\right)+1\right)[\partial_{\tau }\theta \left(\tau \right)f\left(\tau ,t\right)\phi \left(\tau ,t\right)-\frac{1}{2}\partial_{\tau }^{2}\sigma^{2}\left(\tau \right)f\left(\tau ,t\right)\phi \left(\tau ,t\right)] \end{array}$$ (3)

where the *“effective fitness function” f*(*τ*, *t*) ≡ *bϕ*_*G*_(*τ*, *t*)/*ϕ*(*τ*, *t*) and its population average $\bar{f}\left(t\right)=\int_{0}^{\infty }d\tau f\left(\tau ,t\right)\phi \left(\tau ,t\right)$ have been introduced, and where *θ*(*τ*) and *σ*^2^(*τ*) are the first and second cumulants of the variation function *β* (in first approximation we can assume *θ*(*τ*) = 0, while $\sigma^{2}\left(\tau \right)=\alpha_{A}^{2}$ for the additive case and $\sigma^{2}\left(\tau \right)=\alpha_{M}^{2}\tau^{2}$ for the multiplicative case). Observe that, remarkably, this last equation is a generalization of the celebrated continuous-time *Crow-Kimura equation* of population genetics [98], also called *selection-mutation equation* [99, 100]. In particular, notice that the dynamics of the probability density is exposed to the combined action of the process of selection (first term in Eq (3), which is nothing but the *replicator equation* [43, 101]) and mutation, as specified by the drifts in the second line. This type of equations, combining replicator dynamics with Fokker-Planck type of terms—even if with a slightly different interpretation—have been also studied by Sato & Kaneko and Mora & Walzak [102–104]. The main—and crucial—differences between Eq (3) and the standard Crow-Kimura equation are:

- The fitness function appears in the mutation terms—whereas in the standard Crow-Kimura equation the diffusion term would read $\partial_{\tau }^{2}\phi \left(\tau \right)$—thus correlating reproduction rates and mutation amplitudes. Observe that here variations are always associated with reproduction events, as typically in bacteria and viruses, in such a way that a higher fitness rate implies a higher mutation rate.
- There is a general dependence on the cumulants of the variation kernel that, in general, can be trait-dependent and asymmetric.

These generalizations are essential ingredients to capture the essence of our Markovian model as we will see and, to the best of our knowledge, have not been carefully analyzed in the past. From here on, we refer to Eq (3) as the *generalized Crow-Kimura (GCK) equation*.

## Results

In order to scrutinize whether the proposed adaptive stochastic model can account for the key empirical findings of Fridman *et al*. [24], we perform both (i) extensive computational simulations and (ii) numerical studies of the mean-field macroscopic equation, Eq (3).

- Computational simulations rely on the Gillespie algorithm [96], which allows us to simulate exactly the master equation defining the stochastic model. In all cases, we consider at least 10^3^ independent realizations to derive statistically-robust results. Without loss of generality and owing to computational costs, the maximal population size or carrying capacity is fixed to *K* = 10^5^.
- On the other hand, for analytical approaches, in spite of the relatively simple form of Eq (3) owing to its non-linear nature and to the time-variability of environmental conditions *η*(*t*), it is not possible to solve it analytically in a closed way and, thus, it becomes mandatory to resort to numerical-integration schemes. In particular, from this equation—or, more precisely, from integration of its two additive components: Eqs (1) and (2)—one can derive the time-dependent as well as the asymptotic lag-time distributions and, from them, monitor the leading moments or cumulants as a function of time.

Further details of both computational simulations and numerical integration of the macroscopic equation can be found in the Methods section as well as in the S1B. In what follows we present together both types of analyses, underlining where the mean-field approach works well and where its predictions deviate from direct simulations.

### Transient dynamics: Determining variational amplitudes

Parameter values in the model are fixed to agree as much as possible with the empirical ones measured by Fridman et al. [24] (see Methods). In particular, we used (i) the same set of environmental-period durations *T*_*a*_ and *T*_*max*_ − *T*_*a*_ as in the antibiotics/fresh-medium cycle, (ii) the experimentally measured reproduction rate in the fresh medium, (iii) the empirical “falling-asleep” rate *s*, as well as (iv) the same number of antibiotic cycles (ten) as in the experimental setup. Initially all individuals are assumed to have small intrinsic lag-time values of *τ*. In particular, we consider a truncated normal distribution with mean value and variance as in the actual ancestral population in the experiments (〈*τ*〉^*exp*.^ = 1.0 ± 0.2 *h*).

Employing this set of experimentally-constrained parameter values and initial conditions, we ran stochastic simulations in which the whole population expanded and then shrank following the periodically alternating environments. Along this dynamical cyclic process the distribution of *τ* values across the population varies in time; in particular, we monitored the histogram of *τ* values and obtained the corresponding probability distributions right at the end of each antibiotic cycle, just before regrowth, as in the experiments.

**Fig 2:**
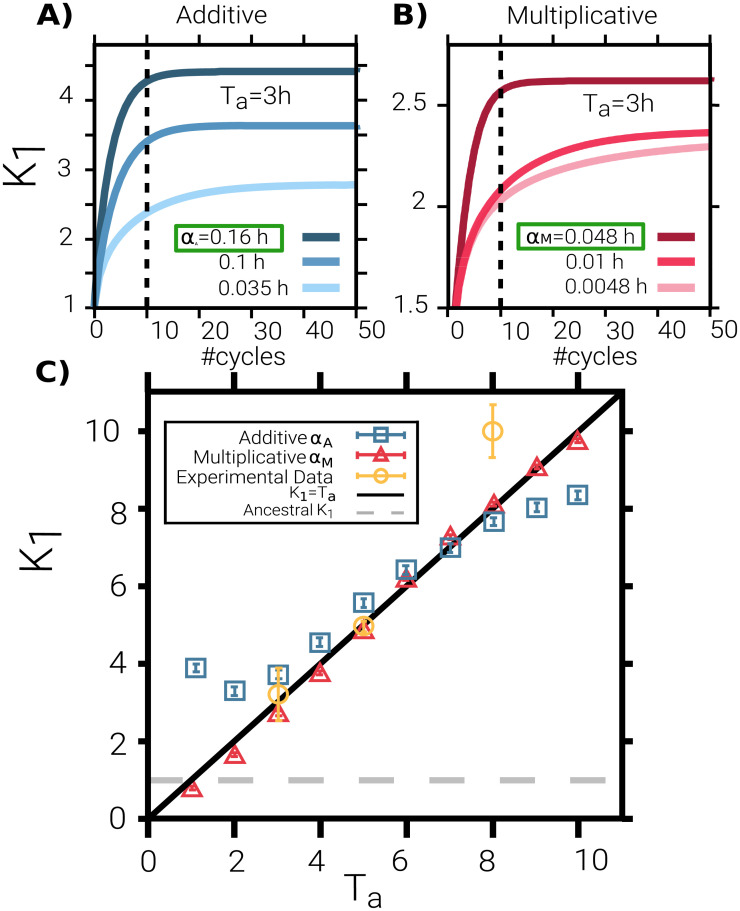
Tuning the only free parameter to match empirical results. **(A-B)** Mean of the lag-time distribution *K*_1_, measured right at the end of each antibiotic cycle, as a function of the number of cycles. Computational results are shown for antibiotic duration *T*_*a*_ = 3*h* for both the additive (A) and the multiplicative (B) versions of the model. The different curves (color coded) correspond to different mutation amplitudes *α*_*A*_ (in A) and *α*_*M*_ (in B), respectively. The dashed vertical lines indicate the 10*th* cycle, when experiments stop. Remarkably, the mean lag time strongly depends on the mutational amplitude, both in the transient regime and in the asymptotic state. We implement an algorithmic search to tune the only free parameter (either *α*_*A*_ or *α*_*M*_) to best fit the experimental mean lag times for all values of *T*_*a*_ together and, in particular, their experimentally-reported linear dependence on the antibiotic exposure time *T*_*a*_ (see Methods). **(C)** Mean of the lag-time distribution as a function of *T*_*a*_ for the model (squares for additive and triangles for multiplicative versions of the model) tuned to reproduce experimental values (yellow symbols). While empirical data are available for *T*_*a*_ = 3, 5 and 8*h*, the model can be analyzed for generic values of *T*_*a*_. The solid line indicates the linear dependence between the mean and lag-time distribution, *K*_1_ = *T*_*a*_, while the horizontal dashed line represents the mean lag time of the ancestral population. Parameter values: *K* = 10^5^, *α*_*A*_ = 0.16*h*, *α*_*M*_ = 0.048, *b* = 2.4*h*^−1^, *d* = 3.6 ⋅ 10^−5^*h*^−1^, *s* = 0.12 *h*^−1^, *T*_*fresh*_ = 23*h* − *T*_*a*_, 10 cycles (see Methods).

**Fig 3:**
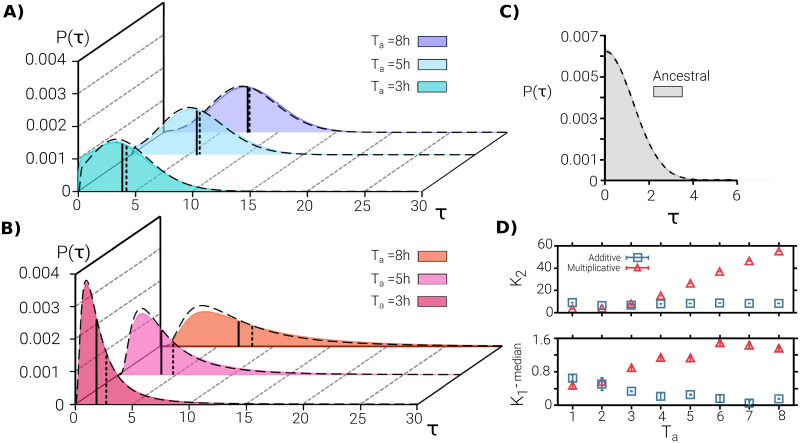
Lag-time probability distributions: Theory and simulations. **(A-B)** Lag-time distribution after 10 cycles as obtained in the simulation of the individual-based model in both the additive (A) and the multiplicative (B) case, for different antibiotic-exposure periods, *T*_*a*_ = 3, 5 and 8*h* (marked with different colours). Solid and dotted vertical lines indicate, respectively, the median and mean of the corresponding distributions (a large separation between these two indicators reflects asymmetries in the distribution such as the emergence of a heavy tail to the right). Dashed lines represent results from the numerical integration of the GCK equation, Eq 3, using the same parameters and external conditions. Observe that the multiplicative model generates much larger tails, reproducing the experimental phenomenology better than the additive one. **(C)** Initial lag-time distribution mimicking the experimentally observed one for the ancestral population. **(D)** Variance, *K*_2_, and difference between mean and median, *K*_1_ − *median*, of the lag-time distribution as a function of *T*_*a*_ in the additive (blue squared symbols) and multiplicative (red triangular symbols) versions of the model. *K*_2_ grows with the antibiotic exposure time in the multiplicative case, while in the additive case it remains nearly constant. The difference between the mean and the median is very small in the additive case, while it increases with *T*_*a*_ almost monotonically in the multiplicative one. In summary, the multiplicative model generates a distribution with a variance that grows with the mean, as well as heavy tails, reproducing well the key experimental findings. Parameter values are as in Fig 2.

Fig 2A and 2B show the evolution of the mean (i.e. the first cumulant, *K*_1_) across cycles, while Fig 3A and 3B illustrate the full distribution and higher-order cumulants after 10 cycles. Observe that the value of *K*_1_ after 10 cycles depends on the choice made for the only remaining free parameter, i.e. the variation-amplitude parameter *α*_*A*_ or *α*_*M*_, for the additive or multiplicative versions of the model, respectively. In order to tune either of these parameters, we imposed that *K*_1_(10) reproduces in the closest way the experimentally determined values, as measured right before the 10*th* regrowth cycle. This tuning procedure leads to *α*_*A*_ = 0.16(1)*h* and *α*_*M*_ = 0.048(1) for the additive and multiplicative cases, respectively (parentheses indicate uncertainty in the last digit); these are the values that best reproduce the empirical findings in the sense of least-square deviation from the available empirical data for different *T*_*a*_’s (see Fig 2C).

Let us remark that both variants of the model are able to reproduce the key experimental feature of generating mean lag times close to *T*_*a*_ (observe, however, that there is always a small deviation in the case *T*_*a*_ = 8 *h*, for which even experimentally, *K*_1_ ≈ 10 *h* > *T*_*a*_). Nevertheless, as illustrated in Fig 3A and 3B there are significant differences between the two variants. In particular, the additive model fails to reproduce the following empirical observations:

- *T*_*a*_-dependent variances,
- large differences between median and mean values, and
- strongly skewed distributions with large tails.

For instance, in the experiments, for *T*_*a*_ = 8 *h*, the difference between the mean and the median is 1.1(1) *h* while in the additive model is 0.15 *h*, i.e. about one order of magnitude smaller. Furthermore, in the experiments, lag times of up to 30 *h* are observed, while in the additive model values above ≈ 15 are exponentially cancelled; i.e. they have an extremely low (negligible) probability to be observed. This is also illustrated in Fig 3D where the second and third cumulants (variance and skewness) of the distribution after 10 cycles are plotted as a function of *T*_*a*_. Observe that both cumulants remain almost constant, revealing the absence of heavy tails for large values of *T*_*a*_.

On the other hand, the multiplicative model is able to reproduce not only the experimental values of the mean but also—with no additional parameter nor fine tuning—(i) the existence of large lag-time variances that increase with *T*_*a*_, (ii) the above-mentioned large differences between the mean and the median (1.3(1) in this case), as well as (iii) heavily skewed lag-time distributions that strongly resemble the empirically measured ones (see Fig 2 in Fridman *et al*. [24]). In particular, lag times of the order of 30 *h* have a non-negligible probability to be observed for *T*_*a*_ = 8 *h* within the multiplicative version of the model, after 10 cycles. The resulting probability and the corresponding cumulants (see Fig 3D depend strongly on *T*_*a*_.

Importantly, the previous results are quite robust against changes in the model. In particular, if growing cells are allowed to switch to dormancy in response of starvation, the mean lag time increases, as expected, but the qualitative shape of the lag-time distribution remains unchanged (see S6). Hence, just by modifying accordingly the parameter *α* allows one to recover the same conclusions.

As a word of caution let us emphasize that the distributions in Fig 3 are not obtained exactly in the same way as the experimental ones. The first are distributions of characteristic times *τ* (inverse of intrinsic transition rates) while the second are the actual lag times *t* measured after regrown in a fresh medium. Actually, the characteristic time *τ*, in our model, is just a proxy for the actual time that it takes for the colony formed by such an individual to be observable or detectable in actual experimental setups. Below we discuss this issue more extensively as well as the possible limitations it implies and extensions of the modelling approach to circumvent them.

Let us also underline that Fig 3 reports not only the results of direct simulations but also the theoretical predictions (dashed lines) derived from numerical integration of the macroscopic equations for the two different cases. The agreement with simulation results is remarkably accurate; the origin of the existing small discrepancies will be analyzed in detail in a forthcoming section.

Thus, the main conclusion of these computational and theoretical analyses is that *state-dependent (multiplicative) variability is needed in order to account for the empirically observed key features of the lag-time distributions emerging after a few antibiotic/fresh-medium cycles*. Once this variant of the model is chosen, a good agreement with experimental findings if obtained by fitting the only free parameter: the amplitude of variations.

### Asymptotic state

**Fig 4:**
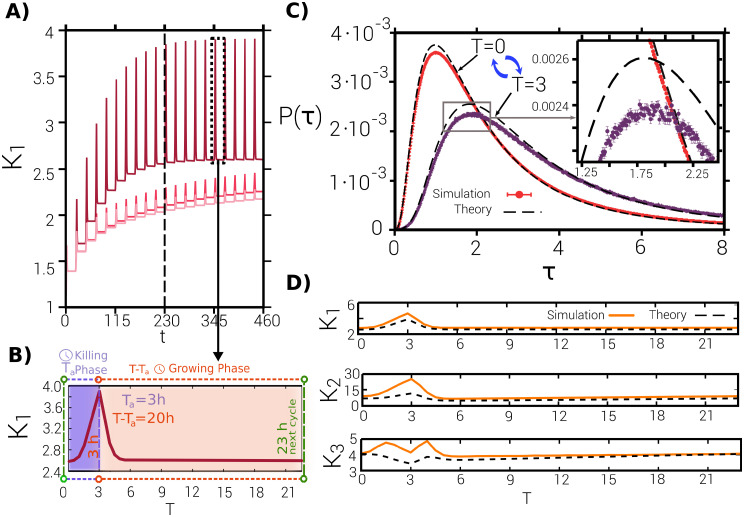
Characterization of the asymptotic state in the multiplicative version of the model. **(A)** Approach to the dynamic asymptotic state for the multiplicative case, as resulting from the integration of Eqs (1) and (2) for *T*_*a*_ = 3*h* (*t* indicates overall time as measured in hours). The different curves correspond to three different values of the variation amplitude (from bottom to top: *α*_*M*_ = 0.0048, 0.01, 0.048). The difference between this plot and Fig 2 is that *K*_1_ is measured at different times within the cycle and not just right at the end of antibiotic exposure (see (B)). The vertical dashed line marks the 10*th* cycle at which the experiment stopped. Observe that the steady-state mean value, the oscillations amplitude, and the relaxation time depend on the variation amplitude *α*_*M*_. **(B)** Mean lag time within a single cycle (*T* ∈ [0, 23]*h*.) in a asymptotic state. During the killing phase (antibiotic exposure), i.e. for *t* < *T*_*a*_, the mean lag time increases to maximize the number of dormant individuals; then, in the fresh medium the mean relaxes back to the initial value. **(C)** Lag-time probability distribution—as derived from theory (dashed lines) and computationally (solid lines)—at the start of the cycle (leftmost curve) and when antibiotics are removed (rightmost curve); in the asymptotic state the system oscillates between these two limiting probability distributions, both of them exhibiting heavy tails. **(D)** Evolution of the three first cumulants *K*_1_, *K*_2_, *K*_3_ (mean, variance, and skewness, respectively) within a asymptotic cycle (both theoretical and computational results are shown). Observe in (C) and (D) that the theory correctly predicts the properties of the distribution but there are some small errors due to finite size effects.

Even if experimental results are available for a fixed and limited number (10) of antibiotic-exposition cycles, the already-calibrated model allows us to scrutinize the possible emergence of asymptotic states after a much-larger number of cycles. In other words, it is possible to go beyond the experimental limits and analyze the fate of the population. In this sense, the experimental results can be seen as a “transient adaptation” to the environment, while the evolutionary cycle would be completed only when an asymptotic (evolutionary stable) state is reached. Let us remark that the asymptotic state is necessarily a *periodic* one, as the phenotypic distributions vary at different instants of the cycle, i.e. the asymptotic distributions—measured at arbitrary times within the cycle—exhibit periodic oscillations in its shape, tracking the perpetual environmental cyclic changes. This is illustrated in Fig 4, showing results obtained by numerically integrating the macroscopic equations, Eqs (1) and (2). First of all, it shows periodic oscillations of the mean lag time *K*_1_; as shown in panel (A) it first increases from its initial value *K*_1_ = 1 and then, eventually, reaches an oscillatory steady state. More specifically, as clearly seen in the zoomed plot of panel (B), within the steady state, the maximum mean value within each cycle is reached right before antibiotics removal. This is an expected result as in the first part of the cycle, i.e. during the “killing phase”, the presence of antibiotics induces a selective pressure towards increasing the mean lag-time value because delaying the exit from the lag phase provides protection from the antibiotics. On the other hand, in the fresh medium (growing phase) the selective pressure quickly reduces the mean lag time to foster fast growth and increased fitness. Thus, summing up, the periodic alternation of environmental conditions induces a stable periodic change in the mean lag-time value.

Actually, it is not only the mean that changes periodically, but the whole probability distribution that varies cyclically. This is illustrated in Fig 4C and 4D which shows computational and theoretical results for the lag-time probability distribution and its first cumulants, *K*_1_, *K*_2_ and *K*_3_, for the multiplicative case (similar plots for the additive case are shown in S1 Text, Sec. S6). Observe, in particular, in panel C, that the distribution oscillates between two extreme or limiting cases corresponding to the times of antibiotics inoculation and antibiotics removal, respectively. This effect can be more vividly seen in the S1 and S2 Videos.

Let us also highlight that the probability distributions exhibit non-Gaussian tails and are right-skewed. In particular, to make these observations more quantitative, Fig 4D shows the variance, *K*_2_, and the skewness, *K*_3_, along the cycle in the steady state. Notice also the very-good—though not perfect—agreement between computational results and theoretical estimates (dashed lines in Fig 4C and 4D).

Furthermore, let us emphasize that, importantly, the amplitude of the variations—as controlled by the parameter *α*_*M*_ (or, similarly, *α*_*A*_ for the additive case)—has a non-trivial effect on both the transient and the asymptotic behavior. In particular, the value of such amplitude not only affects the mean value of lag times after 10 cycles—as illustrated by the plateau of the oscillations in Fig 4A and 4B—but also (i) its asymptotic value, i.e. the mean lag time, (ii) the amplitude of the oscillations across a cycle in the steady state, and (iii) the relaxation time to the asymptotic state (i.e., the speed of evolution). This is due to the heavy tails of the distribution: increasing the amplitude of variations directly increments the variance of lag times, but this also enlarges the left-skewness of the distribution, feeding-back to the mean value. Therefore, the eco-evolutionary attractor is shaped both by selection and mutation, departing from the classical evolutionary scenario, as e.g. in adaptive-dynamics, in which the amplitude of the variations just affects the variance of the resulting distribution but not the overall attractor.

**Fig 5:**
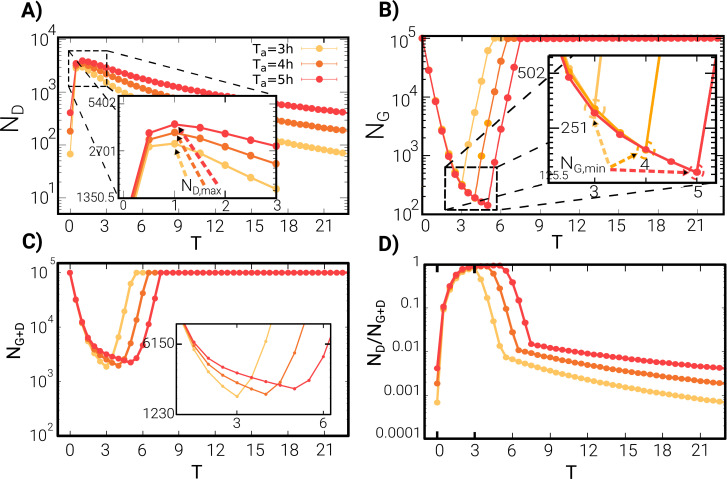
Population dynamics. Abundances *N*_*D*_ and *N*_*G*_ of dormant **(A)** and growing **(B)** cells, respectively, along a full cycle in the asymptotic regime (reached after only three cycles) for the multiplicative model (the curves are the result of averaging over many independent realizations for different *T*_*a*_ as color coded; observe the semi-logarithmic scale). Dormant cells abundances reach a maximum value after approximately one hour of exposition to antibiotics, almost independently of *T*_*a*_, and then start a slow decrease, while *N*_*G*_ exhibits an opposite trend: it rapidly decreases and reaches a minimum at *T* = *T*_*a*_ (see inset), after which it grows exponentially fast until the carrying capacity is reached. **(C)** The total number of cells *N* = *N*_*G*_ + *N*_*D*_ is plotted along the cycle: for all values of *T*_*a*_ the absolute minimum is reached near *T*_*a*_ (as clearly seen in the inset). **(D)** The fraction of dormant cells relative to the total number is maximal nearby *T*_*a*_ and decreases when antibiotics are removed.

Finally, we complement our observations with the population structure dynamics, i.e. proportion, minimum and maximum of dormant and awake cell numbers, in Fig 5. In particular, panels (A) and (B) show the abundances of dormant and awake cells as function of time along an asymptotic cycle. Observe that the number of dormant cells reaches a maximum, *N*_*D*,*max*_, after one hour, independently of the antibiotic duration time *T*_*a*_, while its height is proportional to this parameter. On the other hand, the position of minimum of growing cells number, *N*_*G*,*min*_, scales with *T*_*a*_ and its magnitude decreases correspondently. In Fig 5 we also show and discuss the dependence the total number of cells *N* = *N*_*G*_ + *N*_*D*_ (panel C) as well as the relative fraction of dormant individuals along a full cycle in the stationary state (which is reached after onley a few (three) antibiottic cycles).

For the sake of completeness, let us also emphasize that both versions of the model are able to generate *MDK*_99_ values that grow as a function of the number of antibotic cycles, converging to an asymptotic-state value; at the end of the tenth cycle simulations compare well with empirical observations for different values of *T*_*a*_ (see S10 and S11 Figs; observe that the largest difference appears for *T*_*a*_ = 8, a case for which also *K*_1_ deviates slightly from *T*_*a*_ in the experiments).

### Deviations between theory and simulations: Finite-size effects

Thus far, we have reported results stemming from computational analyses of the individual based model as well as from numerical integration of the associated macroscopic theory, i.e. the GCK equation. Small but systematic discrepancies between theory and simulations are evident, see for example Fig 4C and 4D. Let us here discuss the origin of such differences.

The theoretical approach relies on two different approximations: (i) on the one hand it considers the small-variation approximation to include just the first two moments of the variation function (i.e. a diffusion approximation); (ii) on the other hand, in order to derive the macroscopic GCK equation, one needs to neglect correlations between individuals, a type of mean-field approximation that, as usual, is expected to be exact only in the *infinite population-size limit* [79, 80]. In S1 Text, Sec. S4, we show computational evidence that the small mutation approximation is not a significant source of errors; hence, the discrepancies necessarily stem from finite-size effects. Indeed, in the present experimental set up, there is a bottleneck at the end of each antibiotics cycle, when there is a small number of surviving individuals, thus limiting the validity of the mean-field approximation in such a regime. As a matter of fact, one can clearly see from Fig 4D that the largest discrepancies appear around the end of the killing phase, when the population is the smallest. Note also that the main features of the dynamics in phenotypic space are reproduction and variation: i.e., offspring are similar to their progeny. But reproduction events occur only within the awake (growing) sub-population; the full population-size, involving also dormant ones, is not the most relevant quantity to gauge finite-size effects. Therefore, in order to minimize the discrepancies between theory and simulations it does not suffice to consider larger population sizes: even for huge values of the carrying capacity *K*, we find that the population at the end of the killing phase is always rather small and, hence, exposed to large demographic fluctuations, i.e. to finite-size effects.

**Fig 6:**
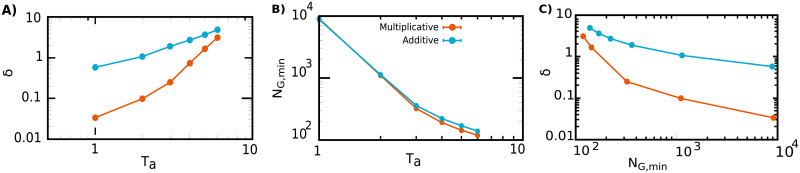
Analysis of the deviations between simulations and theory. The parameter *δ* is defined as the difference between the mean lag times (right at the end of the antibiotic cycle) in the theoretical approach and in computer simulations. **(A)** Double-logarithmic plot reporting the dependence of the error parameter *δ* on the antibiotic time exposure *T*_*a*_ for both the additive (blue dots) and the multiplicative (red dots) versions of the model; in either case, the larger the exposure time the larger the error. **(B)** Minimum number of awake individual during the cycle in the asymptotic regime, *N*_*G*,*min*_, as function of *T*_*a*_ in double-logarithmic scale. As expected, the larger the exposure time the smaller the number of surviving individuals. **(C)** Combining the data from (A) and (B) it follows that *δ* decreases with increasing *N*_*G*,*min*_, meaning that deviations between theory (expected to be exact for infinitely large population sizes) and computational results stem from finite-population-size effects. Notice that errors are smaller in the multiplicative version of the model.

To put these observations on more quantitative bases, we define a parameter *δ* as the deviation between theoretical and computational results for the mean lag-time value after antibiotic exposure and monitor its dependence on the minimal size of the awake population (i.e. right at the end of the antibiotic phase). Fig 6 illustrates that: (A) the deviation grows with the antibiotic-exposure time *T*_*a*_, whereas (B) the minimum awake subpopulation size decreases with *T*_*a*_. Combining these two pieces of information one can see (C) that the deviation parameter *δ* decreases as the minimum subpopulation of awake individuals increases. Unfortunately, the convergence to zero of this last curve is very slow, and thus, it is computationally very expensive to remove finite-size effects.

Finally, let us remark that we leave for future work the formulation of an extension of the mathematical theory accounting for finite-size effects [105–107], including corrections to the GCK equation.

## Conclusions, discussion and perspectives

**Summary of results and conclusions**. We have presented a mathematical and computational model to quantitatively analyze the emergence and evolution of tolerance by lag in bacteria. Our first goal was to reproduce the main results reported in the laboratory experiments of Fridman *et al*. [24] in which the authors found a very fast evolution of tolerance by lag in a community of *Escherichia coli* bacteria periodically exposed to an antibiotics/fresh-medium cycle. In particular, after a relatively small number of such cycles, there is a clear change in the individual-cell lag-time distribution with its mean value evolving to match the duration of antibiotic exposure. This is remarkable, and demonstrates that tolerance by lag is the first and generic strategy adopted by bacteria to survive under harsh environmental conditions such as the presence of antibiotics. A second key empirical finding is that concomitantly with the evolution of the mean lag time, also the variance of lag times is significantly increased for longer antibiotic-exposure periods: i.e. the harsher the conditions the more diversified the lag times within the population. More generally, the full lag-time distribution becomes wider and develops a heavy tail for sufficiently large times. This means that there exist individual phenotypes that are clearly sub-optimal under the strictly controlled laboratory conditions and most-likely reflects a bet-hedging strategy, preparing the community to survive under even harsher conditions (i.e. longer stressful periods).

To shed light onto these observations we developed a stochastic individual-based model assuming that individuals are characterized by an intrinsic lag time, setting the “typical” time at which such individual stochastically wakes up after dormancy. This phenotypic trait is transmitted to the progeny with possible variation. By considering a protocol analogous to the experimental one (i.e. alternating antibiotic exposure and fresh medium growth) the model is able to produce a distribution of characteristic lag times across the population that reproduces quite well the empirical results in all cases by tuning a single parameter value. In particular, the emerging lag-time distributions have a mean that matches the period of antibiotic exposure *T*_*a*_, an increase of the mean and variance with *T*_*a*_, as well as a large difference between the mean and the median, which result from the appearance of heavy tails in the lag-time distributions. Nevertheless, it is important to underline that the distributions that the model generates are just a proxy for the empirically-determined ones, where the actual times in which individual bacteria give rise to new and detectable (i.e. visible with the available technology) colonies are measured.

Importantly, in order to account for all the above empirical phenomenology, the model needs to assume *multiplicative variations*, i.e. that the variability between the parent’s trait and those of its offspring increases (linearly) with the parent’s lag time: the larger the parent’s lag time the larger the possible variation. This multiplicative process—at the roots of the emerging heavy tails in the lag-time distribution—resembles the so-called *rich-get-richer* mechanism of the *Matthew effect* [84–87, 91]. This type of variations implements an effective dependence between the parent’s trait value and the variation amplitude, that was hypothesized as a possible mechanism behind the experimental results and that could stem from a highly non-linear map between genotypic changes and their phenotypic manifestations [24, 69].

Notably, our analyses reveal that the amplitude of variations affects not just the variance (*K*_2_) of the resulting lag-time distribution, but also its mean (*K*_1_) as well as other higher-order cumulants such as the skewness (*K*_3_). This is in blatant contrast with standard approaches to evolutionary or adaptive dynamics, in which the “mutational amplitude” only influences the “broadness” (*K*_2_) of the distribution of traits in phenotypic space, but does not alter the attractor of the dynamics (e.g. *K*_1_). Thus, the introduction of state-dependent (multiplicative) variability constitutes a step forward into our understanding of how simple adaptive/evolutionary processes can generate complex outcomes.

Let us finally mention that our model describes rapid evolution, where ecological and mutational time scales are comparable. This interplay between ecological and evolutionary processes is explicit in the asymptotic state: it is not an “evolutionary stable state” but a *“non-equilibrium evolutionary stable state.”* By non-equilibrium we mean that the detailed-balance condition—a requirement of equilibrium states [108]—is violated and thus, there are net probability fluxes in phenotypic space. These correspond to adaptive oscillations in phenotypic space. Key properties of such a state (oscillation plateaus, amplitudes, etc.) depend on the mutational amplitude, i.e., the amplitude of variations determine the eco-evolutionary attractor. In future work we will scrutinize much in depth non-equilibrium characteristic properties, such as non-vanishing entropy-production of these type of complex eco-evolutionary processes [23, 109, 110].

**Advantages and limitations of the phenotypic-modeling approach**. As already underlined, the present model assumes adaptation at a phenotypic level. Is this a biologically realistic assumption? The answer to this question, in principle, is affirmative but some caveats are in order.

First of all, let us recall that a large part of the theoretical work on evolutionary dynamics and adaptation developed during the last decades focuses on phenotypic adaptation. For instance, in the theory of adaptive dynamics, individuals are always characterized by some phenotypic trait or set of traits which is subject to selection and transmission to the progeny with variation [39–41, 46, 47] (see also e.g. [71, 72]). In general, this is the most parsimonious way of modeling adaptation as the details of the genotypic-phenotypic mapping are usually highly non-linear or simply unknown (see e.g. [95, 111–114]).

On the one hand, adaptation beyond genetic changes—for example epigenetic adaptation—is a well-documented phenomenon in the bacterial world [115] and is the focus of intense research activity [27, 116–119]. For instance, recent work explores “the evolutionary advantage of heritable phenotypic heterogeneity”, which suggests that evolutionary mechanisms at a phenotypic level, such as the ones employed in our approach, might be biologically favored with respect to more-standard genetic mechanisms, under certain circumstances [120]. In particular, such phenotypic variability can provide a faster and more flexible type of response than the one associated with traditional genetic mutations.

Nevertheless, it is important to underline that Fridman *et al*. found empirical evidence that—in their specific setup—genetic mutations were always present in the evolved strains. In particular, they found mutations in genes controlling the so-called toxin-antitoxin circuit, mediating the response to antibiotic stress [24]. This regulatory circuit is known to lead to “multiplicative fluctuations” in the lag-time distribution at the phenotypic level [69]. Thus, strictly speaking, our modeling approach constitutes an effective or phenomenological approximation to the more complex biology of this problem.

This observation opens promising and exciting avenues for future research to shed light on how broad probability distributions of lag times—possibly with heavy tails—can be actually encoded in phenotypic or genetic models. Actually, scale-free (power-law) distributions of bacterial lag times have been recently reported in a specifically-devised experimental setup [121]. Similarly to our conclusions, this work also emphasizes that a broad distribution of individual-cell waking-up rates is needed to generate non-exponential decays of the overall lag-time distribution.

Similarly, another exciting possibility would be to develop computational models akin to the phenotypic one proposed here but implementing genetic circuitry; i.e. models where the phenotype is the (possibly stochastic) outcome of an underlying regulated genetic process and where the object of selection are not specific lag times but their whole distributions as genetically encoded.

**Future developments and perspectives**. In future research, we would like to further delve onto several aspects, both biological and theoretical, of the present work. As a first step, we leave for forthcoming work the analysis of the pertinent question of how similar systems respond to randomly fluctuating environments as opposed to periodically changing ones; do they develop heavier tails to cope with such unpredictable conditions in a sort of bet-hedging strategy? How do the statistical features of the environmental variability translate into the emerging lag-time distributions? [22, 27, 28, 82, 122, 123].

From a more theoretical perspective, we leave for an impending work the formulation of an extension of our approach that fully accounts for finite-size effects, thus introducing the next-to-leading order corrections to the generalized-Crow-Kimura macroscopic equation accounting for demographic fluctuations. Within this context, treating the variation-amplitude itself as an evolving trait is also a potentially fruitful route for further studies.

Finally, as a long-term project we plan to develop models and analytical approaches, similar to the ones explored here, but focusing on genetic evolution, employing explicit genotypic-phenotypic mappings, rather than just on phenotypic changes. In particular, by introducing this further layer of complexity it would be possible to generate more general types of single-cell lag-time distributions, not limited to exponential ones as the purely Markovian approach considered here. Let us recall that a more general stochastic non-Markovian framework—i.e., including memory effects (see e.g. [81, 82, 124])—is a challenging goal that promises to be very pertinent and relevant for many diverse problems in which the control of time is important.

## Methods

### Numerical values of the parameters

In order to fix parameter values we employed the experimental values and measurements in [24] as closely as possible. The number of bacteria involved in the experiment reaches values of the order of ∼ 10^9^; however this number is prohibitively large for computer simulations and we fixed a maximum carrying capacity of *K* = 10^5^, verifying that results do not depend strongly on such a choice (see finite-size effects section). Initially the number of cells in the growing state is fixed to be equal to the carrying capacity *K*; thus no cell is initially in the dormant state). The doubling time of both the ancestral and the evolved populations is 25 ± 0.3 min; thus on average every single bacteria attempts reproduction at a rate *b* = 1/25 min = 2.4 *h*^−1^. The death rate for (natural) causes (i.e. other than antibiotics) is *d* = 3.6 ⋅ 10^−5^ *h*^−1^. The awakening rate is given by the inverse of the characteristic time *a* = 1/*τ* [75]. The initial condition (ancestral or wild population) was randomly sampled from a truncated Gaussian peaked at *τ* = 0. Since the empirical ancestral distribution is narrow and close to the origin [24] (mean lag time ${\langle \tau \rangle }_{0}^{exp.}=1\pm 0.2\;h$) we fix the standard deviation of the truncated Gaussian distribution to *σ* = 1 *h* 16 min in such a way that ${\langle \tau \rangle }_{0}^{sim.}∼1\;h$. Neither the exit rate from dormancy *s* nor the amplitude of the mutations, *α*_*A*_ and *α*_*M*_, can be experimentally measured, but we can fix them indirectly (the rest of the parameters are kept fixed with the values specified above). First, *s* can be chosen using the experimental information that for the ancestral population *MDK*_99_ ∼ 2.55*h*. Hence, we leave the (simulated) ancestral population in the antibiotic phase until the 99% becomes extinct; averaging over different initial conditions, we found that *s* = 0.12 *h*^−1^ is a good approximation. To fix the constants *α*_*A*_ and *α*_*M*_ we performed simulations for diverse values of such parameters and looked for those that best reproduce the experimental tendency after 10 exposure cycles for the different exposure times under consideration (in particular, we performed a least-square deviation analysis to match the straight-line 〈*τ*〉 = *T*_*a*_ when performing a linear interpolation for all *T*_*a*_’s). A systematic sweep of the values of the first two significant digits led us to *α*_*A*_ = 0.16*h* and *α*_*M*_ = 0.048.

### Variation functions

We consider two different variation kernels for lag-time variations $\delta =\tau -\tilde{\tau }$: the additive one, $\beta_{A}\left(\delta ;\tilde{\tau }\right)$ and multiplicative one, $\beta_{M}\left(\delta ;\tilde{\tau }\right)$. Both of them are probability density functions of *δ*, normalized in $\left[-\tilde{\tau },\infty \right]$ and may depend on the initial phenotype $\tilde{\tau }$. In particular, the additive case reads: $\beta_{A}(\delta ;\tilde{\tau })=e^{-\frac{\delta^{2}}{2\alpha_{A}^{2}}}/Z_{A}(\tilde{\tau })$ with $Z_{A}\left(\tau \right)=\alpha_{A}\frac{\sqrt{\pi }}{\sqrt{2}}Erfc\left(-\frac{\tau }{\sqrt{2}\alpha_{A}}\right)$ (where *Erfc* stands for the complementary error function), while in the multiplicative case, we consider $\beta_{M}\left(\delta ;\tilde{\tau }\right)=e^{-\frac{\delta^{2}}{2\alpha_{M}\tilde{\tau }}}/Z_{M}\left(\tilde{\tau }\right)$ with $Z_{M}\left(\tau \right)=\alpha_{M}\tau \sqrt{\frac{\pi }{2}}Erfc\left(-\frac{1}{\sqrt{2}\alpha_{M}}\right)$.

### Measuring lag-time distributions

In order to determine lag-time distributions, we computed histograms in phenotypic space, as discretized in bins of size Δ*τ* = 10^−2^ and averaged over many realizations of the process. In the asymptotic steady state, similar histograms were computed at different times along the antibiotic/fresh-medium cycle (e.g. right after antibiotic inoculation or after antibiotic removal). To obtain results for the transient state we determined the histogram after running for 10 cycles. On the other hand, to determine the steady state, we started measuring after 300 cycles (to make sure that a steady state has been reached) and then collect statistics up to cycle 1500, at intervals of 10 cycles to avoid correlations. We repeated the process for 30 realizations and calculated the histogram as well as the associated cumulants.

### Numerical integration of the macroscopic equation

The parameter set and initial condition for numerical integration of the mean-field equations are the same as specified above. Numerical integration was carried out using the finite differences method. In particular ∂_*t*_ was approximated using first order forward differences, $\partial_{\tau }^{2}$ using second-order centered differences, and integrals were approximated as Riemann sums [125]. The numerical integration steps used in the figures are: *h*_*t*_ = 10^−6^ and *h*_*τ*_ = 10^−2^. Note that, when we calculate the probability distributions during the simulation, we must use the same bin size to be able to correctly compare with the theoretical distributions later. We used absorbing boundary conditions *ϕ*(*τ* = 0, *t*) = *ϕ*(*τ* = *τ*_*max*_, *t*) = 0, where *τ*_*max*_ is the limit of the phenotype space considered for the numerical integration, in particular: *τ*_*max*_ = 60*h*.

## Supporting information

S1 Text Sections: S1: The Microscopic process, S1A: Master equation, S1B: Gillespie algorithm. S2: From Microscopic to Macroscopic Process: S2A. Marginalization, S2B: Mean Field Approximation. S3: Small Variation Approximation. S4: Deviation between theory and simulations, S4A: Validity of the small variation approximation and border effects, S4B: FInite-size effects. S5: Spontaneous shifting to the dormant state. S6: Additional Figures. S7: Movies.
(PDF)

S1 Fig Validity of the small variation approximation in the additive case. Evolution of the first three cumulants (*K*_1_, *K*_2_, *K*_3_) in a asymptotic cycle calculated by the numerical integration of both the general mean-field equation (S95)(solid line) and the generalized Crow-Kimura equation (S114)(points) for two different values of *α*_*A*_, in **(A)** *α*_*A*_ = 0.16*h* and in **(B)** *α*_*A*_ = 0.035*h*. The first variation value is the best fit the experimental results (main text), while the second causes negligible border effects. In both cases the small-variation approximation is a good approximation. Parameter values: *T*_*a*_ = 3*h*, the rest of the parameters, as well as the initial conditions, are kept fixed as specified in the main text).
(EPS)

S2 Fig Range of validity of the small variation approximation in the additive scenario. Systematic comparison of Eq.(S114) and Eq.(S95) via parameter Eq.(S124) for different *α*_*A*_ values, both axis in *log*−scale. Note that the deviation monotonically increases with *α*_*A*_. In particular for *α*_*A*_ = 0.16*h*, the one used in the main text results, the deviation *δ*_*st*._ = (3.5 ± 0.2) ⋅ 10^−2^*h*, Eq.(S124) is small enough to use Eq.(S114). Parameter values: *T*_*a*_ = 3*h*, the other as in the main text.
(EPS)

S3 Fig Validity of the small variation approximation in the multiplicative scenario. Evolution of the first three cumulants (*K*_1_, *K*_2_, *K*_3_) in a asymptotic cycle calculated by the numerical integration of both the general mean-field Eq.(S95)(solid line) and the generalized Crow-Kimura Eq.(S114)(points). The generalized Crow-Kimura equation is a good approximation of the general mean-field equation for the *α*_*M*_-value that best fits the experimental results, e.g.*δ*_*st*._ = (1.5 ± 0.3) ⋅ 10^−3^*h*. Parameter values: *T*_*a*_ = 3*h*, *α*_*M*_ = 0.048, the remaining are specified in the main text.
(EPS)

S4 FigBorder effects in the variation functions.First (**A**-**B**) and second (**C**-**D**) moment of the variation functions as function of the trait *τ* in additive (left) and multiplicative (right) variation cases. In **(A)** the additive case, *θ*_*A*_(*τ*) is positive in *τ* = 0 but decays rapidly to zero as *τ* increases, such that it is sufficient to restrict *τ* axis between 0 and 1. In **(C)** the same is shown for the second moment $\sigma_{A}^{2}\left(\tau \right)$. Note that the magnitude of the dependence decreases with *α*_*A*_). Nevertheless, the value used in the main text, *α*_*A*_ = 0.16*h*, is too large to neglect Eq.(S120) this effect in the generalized Crow-Kimura eq. On the other hand, in the multiplicative case the main text value, *α*_*M*_ = 0.048, is small enough to avoid the border effects in the GCK eq. In **(B)** one can observe that *θ*_*A*_(*τ*) almost vanishes, and in **(C)** that the exact moment $\sigma_{M}^{2}\left(\tau \right)$ coincides quite well with the approximation *α*_*M*_*τ*^2^, Eq.(S123).(EPS)

S5 Fig Schematic definition of the parameter *δ* (illustrated in the multiplicative amplitude scenario). *α*_*M*_ = 0.048, *T*_*a*_ = 6*h*.
(EPS)

S6 Fig **A)** Functional dependence of the rate to enter the dormant state in fresh medium *s*_*f*_ and the number of particles in growing state *N*_*G*_. **B)** and **C)** Lag-time probability distribution function, *P*(*τ*), at the end of the tenth cycle (for the multiplicative case) for a constant *s*_*f*_ and *s*_*f*_ = *s*_*k*_ (tanh[−*cN*_*G*_/*K*]), respectively. The results in the main text, for *s*_*f*_ = 0, are represented by a dashed line. Observe that in both cases the value of *K*_1_, *K*_2_, *K*_3_ increase, but the qualitative form of the distributions remains unchanged. Parameter values: *h*_*τ*_ = 0.01, *α*_*M*_ = 0.048, *T*_*a*_ = 3*h*, *c* = 3, *T* = 23*h*, the rest of parameters are fixed as in the main text.
(EPS)

S7 FigDynamics of the averaged population structure within one cycle.Abundances *N*_*G*_ and *N*_*D*_ along the 10th cycle for *s*_*f*_ constant—**A)** and **C)**—and $s_{f}=\tilde{s}(\text{tanh}[-cN_{G}/K])$—**B)** and **D)**—. The two scenarios are very similar to each other. At the beginning of the cycle *N*_*D*_ is non-vanishing, since bacteria entered this state during the fresh phase of the previous cycle. During the antibiotic exposure phase both, *N*_*D*_ and *N*_*G*_, decrease to a minimum as the bacteria die. When the antibiotic is removed and a fresh medium is added, *N*_*G*_ grows towards the system’s carrying capacity. Observe that the bacteria still enter the dormant state, but the reproduction rate is much higher, in such a way that an overall reduction of *N*_*G*_ is only observed as the system approaches the carrying capacity.(EPS)

S8 Fig Characterization of the asymptotic state in the additive version of the model. **(A)** Relaxation of the mean lag-time to its asymptotic state (curves obtained from the integration of Equation Eq.(S114) with *T*_*a*_ = 3*h* and additive model. The different curves correspond to three different values of the variation amplitude, *α*_*A*_; from the lowest to the highest: *α*_*A*_ = 0.035*h*, *α*_*A*_ = 0.1*h* and *α*_*A*_ = 0.16*h*. **(B)** Zoom of the curve *α*_*A*_ = 0.16*h* for one single cycle. In particular, the mean lag time, *K*_1_, is shown along a cycle in the asymptotic regime. At *T* = 0 the antibiotic is added and the system enters in the “killing phase” (*T* ∈ [0, *T*_*a*_]). When the antibiotic is present, the system experiences a selection pressure towards longer lag times, in consequence, *K*_1_ increases. At *T* = *T*_*a*_ the antibiotic is washed and the fresh medium is added (*T* ∈ [*T*_*a*_, *T*_*max*_]). In this regime, the selection pressure is towards shorter lag times and *K*_1_ relaxes back to the initial value. **(C)** Lag-time probability distribution at *T* = 0 (leftmost curve) and *T* = *T*_*a*_ = 3*h* (rightmost curve) as derived theoretically (Eq.(S114), dashed lines) and computationally (dots). In the asymptotic state the system oscillates between these two limiting probability distributions, both of them exhibiting weak tails. **(D)** Evolution of the first three cumulants, *K*_1_, *K*_2_ and *K*_3_, (mean, variance, and skewness respectively) along a cycle in the asymptotic state (both theoretical and computational results are displayed). Observe that in C/D the theory correctly predicts the properties of the distribution, however there are deviations due to finite size effects.
(EPS)

S9 Fig Simulated number of cells via the Gillespie algorithm. —additive-amplitude scenario, linear scale—. **a)** Number of cell in dormant state during a whole cycle in the asymptotic state for different exposition times *T*_*a*_. **b)** Same as a) but for growing state bacteria. Obviously, the minimum number of bacteria in growing state *N*_*G*,*min*_ is reached at *t* = *T*_*a*_, since, during the antibiotic exposure phase, that number can only decrease. Parameters: *α*_*A*_ = 0.16*h*, the rest are fixed as in the main text.
(EPS)

S10 Fig Evolution of the *MDK*_99_ over 10 exposure cycles. **a)** Additive case. **b)** Multiplicative case. In our simulations, the *MDK*_99_ is calculated analogously to the experimental procedure. After a certain number cycles (i.e. # in the figure) of antibiotics-fresh environment, the evolved population is posed back in the antibiotic phase for a long time. The maximum number of cycles is 10 as in the experiments. The *MDK*_99_ is estimated by the time necessary to kill to the 99% of the population. Both in the additive and multiplicative case, the *MDK*_99_ increases with the # and *T*_*a*_. Interestingly, in the multiplicative case by increasing *T*_*a*_ the change in *MDK*_99_ is bigger than the additive one.
(EPS)

S11 FigComparison of experimental and simulated *MDK*_99_ of the evolved population after 10 cycles of exposure.For both the additive and multiplicative cases, we observe that the simulated *MDK*_99_ falls within—or it is very close—the experimental values (i.e. the mean plus error) for *T*_*a*_ = 3*h* and 5*h*, but outside for the case of *T*_*a*_ = 8*h*. This result is to be expected given the higher noise of the experimental measurements. In particular the experimental mean is ${\langle \tau \rangle }_{T_{a}=8h}^{exp.}=10\pm 1h$ higher than the theoretical prediction of 8*h*.(EPS)

S1 Video This supporting information file contains a video showing the evolution of the asymptotic lag-time probability distribution for the additive version of the model. (MP4)

S2 Video This supporting information file contains a video showing the evolution of the asymptotic lag-time probability distribution for the multiplicative version of the model. (MP4)

## References

[1] Hendry AP. Eco-evolutionary dynamics. Princeton university press; 2016.

[2] Schoener TW. The Newest Synthesis: Understanding the Interplay of Evolutionary and Ecological Dynamics. Science. 2011;331(6016):426–429. doi: 10.1126/science.1193954 21273479

[3] Pelletier F, Garant D, Hendry AP. Eco-evolutionary dynamics. Philosophical Transactions of the Royal Society B: Biological Sciences. 2009;364(1523):1483–1489. doi: 10.1098/rstb.2009.0027 PMC2690510 19414463

[4] Lennon JT, Jones SE. Microbial seed banks: the ecological and evolutionary implications of dormancy. Nature reviews microbiology. 2011;9(2):119–130. doi: 10.1038/nrmicro2504 21233850

[5] Shoemaker WR, Lennon JT. Evolution with a seed bank: the population genetic consequences of microbial dormancy. Evolutionary applications. 2018;11(1):60–75. doi: 10.1111/eva.12557 29302272 PMC5748526

[6] Fraser D, Kaern M. A chance at survival: gene expression noise and phenotypic diversification strategies. Molecular microbiology. 2009;71(6):1333–1340. doi: 10.1111/j.1365-2958.2009.06605.x 19220745

[7] Guppy M, Withers P. Metabolic depression in animals: physiological perspectives and biochemical generalizations. Biological Reviews. 1999;74(1):1–40. doi: 10.1111/j.1469-185X.1999.tb00180.x 10396183

[8] Sturm A, Dworkin J. Phenotypic diversity as a mechanism to exit cellular dormancy. Current Biology. 2015;25(17):2272–2277. doi: 10.1016/j.cub.2015.07.018 26279233 PMC4778719

[9] Barton ES, White DW, Cathelyn JS, Brett-McClellan KA, Engle M, Diamond MS, et al. Herpesvirus latency confers symbiotic protection from bacterial infection. Nature. 2007;447(7142):326–329. doi: 10.1038/nature05762 17507983

[10] Bertrand RL. Lag phase is a dynamic, organized, adaptive, and evolvable period that prepares bacteria for cell division. Journal of bacteriology. 2019;201(7):e00697–18. doi: 10.1128/JB.00697-18 30642990 PMC6416914

[11] van Vliet S. Bacterial dormancy: how to decide when to wake up. Current Biology. 2015;25(17):R753–R755. doi: 10.1016/j.cub.2015.07.039 26325134

[12] Rittershaus ES, Baek SH, Sassetti CM. The normalcy of dormancy: common themes in microbial quiescence. Cell host & microbe. 2013;13(6):643–651. doi: 10.1016/j.chom.2013.05.012 23768489 PMC3743100

[13] Childs DZ, Metcalf C, Rees M. Evolutionary bet-hedging in the real world: empirical evidence and challenges revealed by plants. Proceedings of the Royal Society B: Biological Sciences. 2010;277(1697):3055–3064. doi: 10.1098/rspb.2010.0707 20573624 PMC2982066

[14] Gremer JR, Venable DL. Bet hedging in desert winter annual plants: optimal germination strategies in a variable environment. Ecology Letters. 2014;17(3):380–387. doi: 10.1111/ele.12241 24393387

[15] Rechinger KB, Siegumfeldt H, Svendsen I, Jakobsen M. Early protein synthesis of Lactobacillus delbrueckii ssp. bulgaricus in milk revealed by methionine labeling and two-dimensional gel electrophoresis. Electrophoresis: An International Journal. 2000;21(13):2660–2669. doi: 10.1002/1522-2683(20000701)21:13<2660::AID-ELPS2660>3.0.CO;2-7 10949143

[16] Larsen N, Boye M, Siegumfeldt H, Jakobsen M. Differential expression of proteins and genes in the lag phase of Lactococcus lactis subsp. lactis grown in synthetic medium and reconstituted skim milk. Appl Environ Microbiol. 2006;72(2):1173–1179. doi: 10.1128/AEM.72.2.1173-1179.2006 16461664 PMC1392913

[17] Van Bodegom P. Microbial maintenance: a critical review on its quantification. Microbial ecology. 2007;53(4):513–523. doi: 10.1007/s00248-006-9049-5 17333428 PMC1915598

[18] Paredes-Sabja D, Setlow P, Sarker MR. Germination of spores of Bacillales and Clostridiales species: mechanisms and proteins involved. Trends in microbiology. 2011;19(2):85–94. doi: 10.1016/j.tim.2010.10.004 21112786

[19] Wright ES, Vetsigian KH. Stochastic exits from dormancy give rise to heavy-tailed distributions of descendants in bacterial populations. Molecular Ecology. 2019;28(17):3915–3928. doi: 10.1111/mec.15200 31355980

[20] Epstein SS. Microbial awakenings. Nature. 2009;457(7233):1083–1083. doi: 10.1038/4571083a 19242455

[21] Buerger S, Spoering A, Gavrish E, Leslin C, Ling L, Epstein S, et al. Microbial scout hypothesis, stochastic exit from dormancy, and the nature of slow growers. Appl Environ Microbiol. 2012;78(9):3221–3228. doi: 10.1128/AEM.07307-11 22367083 PMC3346438

[22] Kussell E, Leibler S. Phenotypic Diversity, Population Growth, and Information in Fluctuating Environments. Science. 2005;309(5743):2075–2078. doi: 10.1126/science.1114383 16123265

[23] Leibler S, Kussell E. Individual histories and selection in heterogeneous populations. Proceedings of the National Academy of Sciences. 2010;107(29):13183–13188. doi: 10.1073/pnas.0912538107 20616073 PMC2919897

[24] Fridman O, Goldberg A, Ronin I, Shoresh N, Balaban NQ. Optimization of lag time underlies antibiotic tolerance in evolved bacterial populations. Nature. 2014;513:418–421. doi: 10.1038/nature13469 25043002

[25] Levin-Reisman I, Ronin I, Gefen O, Braniss I, Shoresh N, Balaban NQ, et al. Antibiotic tolerance facilitates the evolution of resistance. Science. 2017;355(6327):826–830. doi: 10.1126/science.aaj2191 28183996

[26] Philippi T, Seger J. Hedging one’s evolutionary bets, revisited. Trends in Ecology and Evolution. 1989;4(2):41—44. doi: 10.1016/0169-5347(89)90138-9 21227310

[27] Veening JW, Smits WK, Kuipers OP. Bistability, Epigenetics, and Bet-Hedging in Bacteria. Annual Review of Microbiology. 2008;62(1):193–210. doi: 10.1146/annurev.micro.62.081307.163002 18537474

[28] Villa Martín P, Muñoz MA, Pigolotti S. Bet-hedging strategies in expanding populations. PLoS computational biology. 2019;15(4):e1006529. doi: 10.1371/journal.pcbi.1006529 30998676 PMC6490941

[29] Xu Y, Vetsigian K. Phenotypic variability and community interactions of germinating Streptomyces spores. Scientific reports. 2017;7(1):1–13. doi: 10.1038/s41598-017-00792-7 28386097 PMC5429633

[30] Kussell E. Evolution in Microbes. Annual Review of Biophysics. 2013;42(1):493–514. doi: 10.1146/annurev-biophys-083012-130320 23654305

[31] Lenski RE. Experimental evolution and the dynamics of adaptation and genome evolution in microbial populations. The ISME Journal: Multidisciplinary Journal of Microbial Ecology. 2017;11(10):2181–2194. doi: 10.1038/ismej.2017.69 28509909 PMC5607360

[32] Good B, McDonald M, Barrick J, Lenski R, Desai M. The dynamics of molecular evolution over 60,000 generations. Nature. 2017;551(7678):45–50. doi: 10.1038/nature24287 29045390 PMC5788700

[33] Elena SF, Lenski RE. Evolution experiments with microorganisms: the dynamics and genetic bases of adaptation. Nature Reviews Genetics. 2003;4(6):457–469. doi: 10.1038/nrg1088 12776215

[34] Müller M. Ueber den Einfluss von Fiebertemperaturen auf die Wachsthumsgeschwindigkeit und die Virulenz des Typhus. Zeitschrift für Hygiene und Infektionskrankheiten, medizinische Mikrobiologie, Immunologie und Virologie. 1895;20:245.

[35] Brauner A, Fridman O, Gefen O, Balaban NQ. Distinguishing between resistance, tolerance and persistence to antibiotic treatment. Nature Reviews Microbiology. 2016;14(5):320. doi: 10.1038/nrmicro.2016.34 27080241

[36] Kohanski MA, Dwyer DJ, Collins JJ. How antibiotics kill bacteria: from targets to networks. Nature Reviews Microbiology. 2010;8(6):423–435. doi: 10.1038/nrmicro2333 20440275 PMC2896384

[37] Li B, Qiu Y, Shi H, Yin H. The importance of lag time extension in determining bacterial resistance to antibiotics. Analyst. 2016;141(10):3059–3067. doi: 10.1039/C5AN02649K 27077143

[38] Brauner A, Shoresh N, Fridman O, Balaban NQ. An experimental framework for quantifying bacterial tolerance. Biophysical journal. 2017;112(12):2664–2671. doi: 10.1016/j.bpj.2017.05.014 28636922 PMC5479142

[39] Geritz SAH, Metz JAJ, Kisdi E, Meszéna G. Dynamics of Adaptation and Evolutionary Branching. Phys Rev Lett. 1997;78:2024–2027. doi: 10.1103/PhysRevLett.78.2024

[40] Geritz SAH, Metz JAJ, Kisdi E, Meszéna G. Evolutionarily singular strategies and the adaptive growth and branching of the evolutionary tree. Evolutionary Ecology. 1998;12:35–37. doi: 10.1023/A:1006554906681

[41] Diekmann O D.O. 2004. A beginner’s guide to adaptive dynamics school. Banach Center Publications; 2004.

[42] Dieckmann U, Metz JA. Surprising evolutionary predictions from enhanced ecological realism. Theoretical Population Biology. 2006;69(3):263–281. doi: 10.1016/j.tpb.2005.12.001 16469342

[43] Hofbauer J, Sigmund K. Evolutionary game dynamics. Bulletin of the American Mathematical Society. 2003;40(4):479–519. doi: 10.1090/S0273-0979-03-00988-1

[44] Dieckmann U, Law R. The dynamical theory of coevolution: a derivation from stochastic ecological processes. Journal of mathematical biology. 1996;34(5-6):579–612. doi: 10.1007/BF02409751 8691086

[45] Doebeli M, Dieckmann U. Evolutionary branching and sympatric speciation caused by different types of ecological interactions. The american naturalist. 2000;156(S4):S77–S101. doi: 10.1086/303417 29592583

[46] Dieckmann Ulf D M. On the origin of species by sympatric speciation. Nature. 1999;400:354–357. doi: 10.1038/22521 10432112

[47] Doebeli M, Dieckmann U. Speciation along environmental gradients. Nature. 2003;421(6920):259–264. doi: 10.1038/nature01274 12529641

[48] Dieckmann U, Doebeli M, Metz JA, Tautz D. Adaptive Speciation. Cambridge Studies in Adaptive Dynamics. Cambridge University Press; 2004.

[49] Spencer CC, Tyerman J, Bertrand M, Doebeli M. Adaptation increases the likelihood of diversification in an experimental bacterial lineage. Proceedings of the National Academy of Sciences. 2008;105(5):1585–1589. doi: 10.1073/pnas.0708504105 PMC2234188 18216261

[50] Friesen ML, Saxer G, Travisano M, Doebeli M. Experimental evidence for sympartric ecological diversification due to frequency-dependent competition in escherirchia coli. Evolution. 2004;58(2):245—260. doi: 10.1111/j.0014-3820.2004.tb01642.x 15068343

[51] Fletcher JA, Doebeli M. A simple and general explanation for the evolution of altruism. Proceedings of the Royal Society B: Biological Sciences. 2009;276(1654):13–19. doi: 10.1098/rspb.2008.0829 18765343 PMC2614248

[52] Doebeli M, Hauert C, Killingback T. The Evolutionary Origin of Cooperators and Defectors. 2004;306(5697):859–862. 10.1126/science.1101456 15514155

[53] Doebeli M, Ruxton GD. Evolution of disperal rates in metapopulations models: branching and cyclic dynamics in phenotype space. Evolution;51(6):1730–1741. doi: 10.1111/j.1558-5646.1997.tb05097.x 28565110

[54] Champagnat N, Ferrière R, Méléard S. Unifying evolutionary dynamics: From individual stochastic processes to macroscopic models. Theoretical Population Biology. 2006;69(3):297—321. doi: 10.1016/j.tpb.2005.10.004 16460772

[55] Henriques GJB, Ito K, Hauert C, Doebeli M. On the importance of evolving phenotype distributions on evolutionary diversification. PLOS Computational Biology. 2021;17(2):1–21. doi: 10.1371/journal.pcbi.1008733 33591967 PMC7909671

[56] Rubin IN, Doebeli M. Rethinking the evolution of specialization: A model for the evolution of phenotypic heterogeneity. Journal of Theoretical Biology. 2017;435:248–264. doi: 10.1016/j.jtbi.2017.09.020 28943404

[57] Doebeli M, Yaroslav. Towards a mechanistic foundation of evolutionary theory. Elife. 2017;6(:e23804). doi: 10.7554/eLife.23804 28198700 PMC5333952

[58] Kotil Seyfullah K Vetsigian Enes. Emergence of evolutionarily stable communities through eco-evolutionary tunnelling. Nature Ecology and Evolution. 2018;. doi: 10.1038/s41559-018-0655-7 30242295

[59] Iwasa Y, Michor F, Nowak MA. Stochastic Tunnels in Evolutionary Dynamics. Genetics. 2004;166(3):1571–1579. 15082570 10.1534/genetics.166.3.1571 PMC1470783

[60] Hiltunen T, Virta M, Laine AL. Antibiotic resistance in the wild: an eco-evolutionary perspective. Philosophical Transactions of the Royal Society B: Biological Sciences. 2017;372(1712):20160039. doi: 10.1098/rstb.2016.0039 PMC5182435 27920384

[61] Cordero OX, Polz MF. Explaining microbial genomic diversity in light of evolutionary ecology. Nature reviews Microbiology. 2014;12(4):263—273. doi: 10.1038/nrmicro3218 24590245

[62] Sanchez A, Gore J. Feedback between Population and Evolutionary Dynamics Determines the Fate of Social Microbial Populations. PLOS Biology. 2013;11(4):1–9. doi: 10.1371/journal.pbio.1001547 23637571 PMC3640081

[63] Callahan BJ, Fukami T, Fisher DS. Rapid evolution of adaptive niche construction in experimental microbial populations. Evolution. 2014;68(11):3307–3316. doi: 10.1111/evo.12512 25138718

[64] Bauer M, Knebel J, Lechner M, Pickl P, Frey E. Ecological feedback in quorum-sensing microbial populations can induce heterogeneous production of autoinducers. Elife. 2017;6:e25773. doi: 10.7554/eLife.25773 28741470 PMC5526673

[65] Frey E, Knebel J, Pickl P. Mean-field equation for a stochastic many-particle model of quorum-sensing microbial populations. arXiv preprint arXiv:180205307. 2018.

[66] Geritz SA, Metz JA, Kisdi É, Meszéna G. Dynamics of adaptation and evolutionary branching. Physical Review Letters. 1997;78(10):2024. doi: 10.1103/PhysRevLett.78.2024

[67] Sireci M, Muñoz MA. Statistical physics of phenotypic evolution: Adaptive dynamics and beyond.

[68] Buerger R. The Mathematical Theory of Selection, Recombination, and Mutation. John Wiley & Sons; 2000.

[69] Rotem E, Loinger A, Ronin I, Levin-Reisman I, Gabay C, Shoresh N, et al. Regulation of phenotypic variability by a threshold-based mechanism underlies bacterial persistence. Proceedings of the National Academy of Sciences. 2010;107(28):12541–12546. doi: 10.1073/pnas.1004333107 20616060 PMC2906590

[70] Messer PW, Ellner SP, Hairston NG Jr. Can population genetics adapt to rapid evolution? Trends in Genetics. 2016;32(7):408–418. doi: 10.1016/j.tig.2016.04.005 27185237

[71] Bonachela JA, Wortel MT, Stenseth NC. Eco-evolutionary Red Queen dynamics regulate biodiversity in a metabolite-driven microbial system. Scientific reports. 2017;7(1):1–9. doi: 10.1038/s41598-017-17774-4 29247226 PMC5732168

[72] Vallina SM, Martinez-Garcia R, Smith SL, Bonachela JA. Models in microbial ecology. In: Encyclopedia of Microbiology. Elsevier; 2019. p. 211–246.

[73] Wortel MT, Peters H, Bonachela JA, Stenseth NC. Continual evolution through coupled fast and slow feedbacks. Proceedings National Academy of Sciences. 2020;117(8):4234–4242. doi: 10.1073/pnas.1916345117 32029592 PMC7049158

[74] Levin-Reisman I, Gefen O, Fridman O, Ronin I, Shwa D, Sheftel H, et al. Automated imaging with ScanLag reveals previously undetectable bacterial growth phenotypes. Nature methods. 2010;7(9):737—739. doi: 10.1038/nmeth.1485 20676109

[75] Balaban NQ, Merrin J, Chait R, Kowalik L, Leibler S. Bacterial Persistence as a Phenotypic Switch. Science. 2004;305(5690):1622–1625. doi: 10.1126/science.1099390 15308767

[76] Baranyi J. Stochastic modelling of bacterial lag phase. International journal of food microbiology. 2002;73(2-3):203–206. doi: 10.1016/S0168-1605(01)00650-X 11934028

[77] Métris A, Le Marc Y, Elfwing A, Ballagi A, Baranyi J. Modelling the variability of lag times and the first generation times of single cells of E. coli. International journal of food microbiology. 2005;100(1-3):13–19. doi: 10.1016/j.ijfoodmicro.2004.10.004 15854688

[78] Moreno-Gámez S, Kiviet DJ, Vulin C, Schlegel S, Schlegel K, van Doorn GS, et al. Wide lag time distributions break a trade-off between reproduction and survival in bacteria. Proceedings of the National Academy of Sciences. 2020;117(31):18729–18736. doi: 10.1073/pnas.2003331117 PMC7414188 32669426

[79] Van Kampen NG. Stochastic processes in physics and chemistry. vol. 1. Elsevier; 1992.

[80] Gardiner C. Stochastic Methods: A Handbook for the Natural and Social Sciences. Springer Series in Synergetics. Springer; 2009.

[81] Norman TM, Lord ND, Paulsson J, Losick R. Stochastic switching of cell fate in microbes. Annual review of microbiology. 2015;69:381–403. doi: 10.1146/annurev-micro-091213-112852 26332088

[82] Himeoka Y, Mitarai N. When to wake up? The optimal waking-up strategies for starvation-induced persistence. PLoS computational biology. 2021;17(2):e1008655. doi: 10.1371/journal.pcbi.1008655 33571191 PMC7904209

[83] Moran PAP. Random processes in genetics. Mathematical Proceedings of the Cambridge Philosophical Society. 1958;54(1):60–71. doi: 10.1017/S0305004100033193

[84] Sornette D. Multiplicative processes and power laws. Phys Rev E. 1998;57(4):4811. doi: 10.1103/PhysRevE.57.4811

[85] Barabási AL, Albert R. Emergence of scaling in random networks. Science. 1999;286(5439):509–512. doi: 10.1126/science.286.5439.509 10521342

[86] Mitzenmacher M. A Brief History of Generative Models for Power Law and Lognormal Distributions. Internet Mathematics. 2002;1:226–251. doi: 10.1080/15427951.2004.10129088

[87] Newman MEJ. Power laws, Pareto distributions and Zipf’s law. Contemp Phys. 2005;46(5):323–351. doi: 10.1080/00107510500052444

[88] Manrubia SC, Zanette DH. Stochastic multiplicative processes with reset events. Physical Review E. 1999;59(5):4945. doi: 10.1103/PhysRevE.59.4945 11969447

[89] Grinstein G, Muñoz MA, Tu Y. Phase structure of systems with multiplicative noise. Physical Review Letters. 1996;76(23):4376. doi: 10.1103/PhysRevLett.76.4376 10061274

[90] Tu Y, Grinstein G, Muñoz MA. Systems with multiplicative noise: critical behavior from KPZ equation and numerics. Physical Review Letters. 1997;78(2):274. doi: 10.1103/PhysRevLett.78.274

[91] Muñoz M. Multiplicative noise in non-equilibrium phase transitions: A tutorial; in Advances in Condensed Matter and Statistical Physics, Ed. Koroutcheva E. and Cuerno R.; 2004.

[92] Muñoz MA, Colaiori F, Castellano C. Mean-field limit of systems with multiplicative noise. Physical Review E. 2005;72(5):056102. doi: 10.1103/PhysRevE.72.056102 16383683

[93] Genovese W, Muñoz MA. Recent results on multiplicative noise. Physical Review E. 1999;60(1):69. doi: 10.1103/PhysRevE.60.69 11969738

[94] van Nimwegen E. Influenza Escapes Immunity Along Neutral Networks. Science. 2006;314(5807):1884–1886. doi: 10.1126/science.1137300 17185589

[95] Koelle K, Cobey S, Grenfell B, Pascual M. Epochal Evolution Shapes the Phylodynamics of Interpandemic Influenza A (H3N2) in Humans. Science. 2006;314(5807):1898–1903. doi: 10.1126/science.1132745 17185596

[96] Gillespie DT. A general method for numerically simulating the stochastic time evolution of coupled chemical reactions. Journal of computational physics. 1976;22(4):403–434. doi: 10.1016/0021-9991(76)90041-3

[97] Kimura M. Diffusion models in population genetics. Journal of Applied Probability. 1964;1(2):177–232. doi: 10.2307/3211856

[98] Crow JF, Kimura M, et al. An introduction to population genetics theory. An introduction to population genetics theory. 1970.

[99] Hofbauer J. The selection mutation equation. Journal of mathematical biology. 1985;23(1):41–53. doi: 10.1007/BF00276557 4078498

[100] Page KM, Nowak MA. Unifying evolutionary dynamics. Journal of theoretical biology. 2002;219(1):93–98. doi: 10.1016/S0022-5193(02)93112-7 12392978

[101] Nowak MA. Evolutionary dynamics: exploring the equations of life. Harvard University Press; 2006.

[102] Sato K, Kaneko K. On the distribution of state values of reproducing cells. Physical biology. 2006;3(1):74. doi: 10.1088/1478-3975/3/1/008 16582472

[103] Sato K, Kaneko K. Evolution equation of phenotype distribution: General formulation and application to error catastrophe. Physical Review E. 2007;75(6):061909. doi: 10.1103/PhysRevE.75.061909 17677302

[104] Mora T, Walczak AM. Effect of phenotypic selection on stochastic gene expression. The Journal of Physical chemistry B. 2013;117(42):13194–13205. doi: 10.1021/jp403231f 23795617

[105] Cremer J, Melbinger A, Frey E. Evolutionary and population dynamics: a coupled approach. Physical Review E. 2011;84(5):051921. doi: 10.1103/PhysRevE.84.051921 22181458

[106] Cremer J, Melbinger A, Frey E. Growth dynamics and the evolution of cooperation in microbial populations. Scientific reports. 2012;2(1):1–6. doi: 10.1038/srep00281 22355791 PMC3282947

[107] Wakano JY, Iwasa Y. Evolutionary Branching in a Finite Population: Deterministic Branching vs. Stochastic Branching. Genetics. 2013;193(1):229–241. 23105010 10.1534/genetics.112.144980 PMC3527248

[108] Gnesotto FS, Mura F, Gladrow J, Broedersz CP. Broken detailed balance and non-equilibrium dynamics in living systems: a review. Reports on Progress in Physics. 2018;81(6):066601. doi: 10.1088/1361-6633/aab3ed 29504517

[109] Mustonen V, Lässig M. Fitness flux and ubiquity of adaptive evolution. Proceedings of the National Academy of Sciences. 2010;107(9):4248–4253. doi: 10.1073/pnas.0907953107 20145113 PMC2840135

[110] Kussell E, Vucelja M. Non-equilibrium physics and evolution—adaptation, extinction, and ecology: a Key Issues review. Reports on Progress in Physics. 2014;77(10):102602. doi: 10.1088/0034-4885/77/10/102602 25303141

[111] Dalziel AC, Rogers SM, Schulte PM. Linking genotypes to phenotypes and fitness: how mechanistic biology can inform molecular ecology. Molecular ecology. 2009;18(24):4997–5017. doi: 10.1111/j.1365-294X.2009.04427.x 19912534

[112] Arias CF, Catalán P, Manrubia S, Cuesta JA. toyLIFE: a computational framework to study the multi-level organisation of the genotype-phenotype map. Scientific reports. 2014;4(1):1–10. 10.1038/srep07549 PMC4269896 25520296

[113] Manrubia S, Cuesta JA. Distribution of genotype network sizes in sequence-to-structure genotype–phenotype maps. Journal of The Royal Society Interface. 2017;14(129):20160976. doi: 10.1098/rsif.2016.0976 28424303 PMC5414908

[114] Manrubia S, Cuesta JA, Aguirre J, Ahnert SE, Altenberg L, Cano AV, et al. From genotypes to organisms: State-of-the-art and perspectives of a cornerstone in evolutionary dynamics. arXiv preprint arXiv:200200363. 2020. 10.1016/j.plrev.2021.03.004 34088608

[115] Novick A, Weiner M. Enzyme induction as an all-or-none phenomenon. Proceedings of the National Academy of Sciences (USA). 1957;43(7):553. doi: 10.1073/pnas.43.7.553 16590055 PMC528498

[116] Casadesús J, Low D. Epigenetic gene regulation in the bacterial world. Microbiology and molecular biology reviews. 2006;70(3):830–856. doi: 10.1128/MMBR.00016-06 16959970 PMC1594586

[117] Casadesús J, Low DA. Programmed heterogeneity: epigenetic mechanisms in bacteria. Journal of Biological Chemistry. 2013;288(20):13929–13935. doi: 10.1074/jbc.R113.472274 PMC3656251 23592777

[118] Ronin I, Katsowich N, Rosenshine I, Balaban NQ. A long-term epigenetic memory switch controls bacterial virulence bimodality. Elife. 2017;6:e19599. doi: 10.7554/eLife.19599 28178445 PMC5295817

[119] Mutlu A, Trauth S, Ziesack M, Nagler K, Bergeest JP, Rohr K, et al. Phenotypic memory in Bacillus subtilis links dormancy entry and exit by a spore quantity-quality tradeoff. Nature Comm. 2018;9(1):1–12. doi: 10.1038/s41467-017-02477-1 29302032 PMC5754360

[120] Carja O, Plotkin JB. The evolutionary advantage of heritable phenotypic heterogeneity. Scientific reports. 2017;7(1):1–12. doi: 10.1038/s41598-017-05214-2 28698577 PMC5505965

[121] Şimşek E, Kim M. Power-law tail in lag time distribution underlies bacterial persistence. Proceedings of the National Academy of Sciences. 2019;116(36):17635–17640. doi: 10.1073/pnas.1903836116 31427535 PMC6731627

[122] Hidalgo J, Pigolotti S, Muñoz MA. Stochasticity enhances the gaining of bet-hedging strategies in contact-process-like dynamics. Phys Rev E. 2015;91:032114. doi: 10.1103/PhysRevE.91.032114 25871061

[123] Villa Martin P, Hidalgo J, Rubio de Casas R, Muñoz MA. Eco-evolutionary model of rapid phenotypic diversification in species-rich communities. PLoS computational biology. 2016;12(10):e1005139. doi: 10.1371/journal.pcbi.1005139 27736874 PMC5063285

[124] Zhang J, Zhou T. Computation of stationary distributions in stochastic models of cellular processes with molecular memory. bioRxiv. 2019; p. 521575.

[125] Pearson CE. Numerical methods in engineering & science. CRC Press; 1986.

