# Supplementary material for: Phenotypic-dependent variability and the emergence of tolerance in bacterial populations
Source: PLoS Comput Biol. 2021 Sep 23;17(9):e1009417. doi: 10.1371/journal.pcbi.1009417 (PMC8492070; doi:10.1371/journal.pcbi.1009417)
Supplement: S1 Text — Sections: S1: The Microscopic process, S1A: Master equation, S1B: Gillespie algorithm. S2: From Microscopic to Macroscopic Process: S2A. Marginalization, S2B: Mean Field Approximation. S3: Small Variation Approximation. S4: Deviation between theory and simulations, S4A: Validity of the small variation approximation and border effects, S4B: FInite-size effects. S5: Spontaneous shifting to the dormant state. S6: Additional Figures. S7: Movies. (PDF) [file pcbi.1009417.s001.pdf]

Supplementary information
Phenotypic-dependent variability and the emergence of tolerance in bacterial populations

José Camacho Mateu,[1] Matteo Sireci,[2] and Miguel A. Muñoz[2]

[1] *Departamento de Matemáticas, Universidad Carlos III de Madrid, Leganés, Spain*
[2] *Departamento de Electromagnetismo y Física de la Materia and Instituto Carlos I de Física Teórica y Computacional. Universidad de Granada. Granada, Spain*

(Dated: This manuscript was compiled on September 9, 2021)

CONTENTS

S1. The Microscopic process 2
A. Master equation 3
B. Gillespie algorithm 7

S2. From Microscopic to Macroscopic Process 8
A. Marginalization 8
B. Mean Field Approximation 14

S3. Small Variation Approximation 16

S4. Deviation between theory and simulations 19
A. Validity of the small variation approximation and border effects 19
B. Finite-size effects 21

S5. Spontaneous shifting to the dormant state 22

S6. Additional Figures 24

S7. Movies 27

References 27

S1. THE MICROSCOPIC PROCESS

Consider the dynamics of a population of N individual bacteria cyclically exposed to antibiotics, as described in the main text. Each bacteria is characterized by its intrinsic lag time $\tau_i \in \mathcal{P} = \mathbb{R}^+$, where $\mathcal{P}$ denotes the continuous one$-$dimensional phenotype space of possible lag time values. In addition, each individual can be in two different states, as indicated by the variable y_i: if $y_i = 1$ the individual is in the awake state, such that it can reproduce, while if $y_i = -1$ it is in dormant state, where no activity happens. Finally, the environment, represented by the parameter η, varies between two states characterized by the presence ($\eta = -1$) or absence ($\eta = 1$) of antibiotics. The environmental dynamics is considered to be cyclical with period T_{max}, $\eta(t) = \eta(t + T_{max})$, and within each cycle:

$$\eta = \begin{cases} -1 & \text{if} \quad 0 \leq T \leq T_a \\ +1 & \text{if} \quad T_a < T \leq T_{max} \end{cases} \tag{S1}$$

where $T \in [0, T_{max}]$ is the time within a cycle and T_a is the duration of the antibiotic phase.

The whole population state is thus represented by the vectors $\boldsymbol{\tau}_N = (\tau_1, \tau_2, .., \tau_i, .., \tau_N)$ and $\boldsymbol{y}_N = (y_1, y_2, .., y_i, .., y_N)$. The vector subindex indicates the dimensionality, i.e., the number of bacteria: $dim(\boldsymbol{\tau}_N) = N, dim(\tilde{\boldsymbol{\tau}}^{i,\hat{j}}_{N-1}) = N - 1$. To further proceed, let us consider that all individuals are indistinguishable, i.e., that the system state is invariant under individual permutations; for example: $\boldsymbol{\tau}_N = (\tau_1, \tau_2, .., \tau_i, .., \tau_N) = \boldsymbol{\tau'_N} = (\tau_i, \tau_1, \tau_N, \ldots, \tau_2, \ldots, \tau_3)$. The following notation is implemented in order to account for changes in the population; consider a reference state $\boldsymbol{\tau}_N = (\tau_1, \tau_2, \ldots, \tau_i, \ldots, \tau_j, \ldots, \tau_N)$; then a "modified" state with respect to $\boldsymbol{\tau}_N$ is defined as $\tilde{\boldsymbol{\tau}}^{i,\hat{j}}_{N-1} = (\tau_1, \tau_2, \ldots, \tilde{\tau}_i, \ldots, \tau_{j-1}, \tau_{j+1}, .., \tau_N) = (\tau_1, \tau_2, \ldots, \tilde{\tau}_i, \ldots, \tau_{N-1})$, where the i individual has a different trait, $\tilde{\tau}_i \neq \tau_i$, and the j one is absent, while the rest of individuals have the same traits in both states. Note that the property of indistinguishability has been used to derive the last equality.

Having defined the states, one can define the dynamics. In particular, each individual cell can perform diverse stochastic "reactions" (using the jargon of stochastic processes), that are illustrated in Fig. 1 of the main text.

Any individual i in the growing state($y_i = 1$) with trait $\tilde{\tau}_i$ is exposed to a demographic-adaptive process (Δ_D):

- It can try to reproduce asexually at rate $g(\tau_i, \boldsymbol{\tau}_N) = b \in \mathbb{R}^+$. On the one hand, if the antibiotics are present ($\eta = -1$), the reproduction does not happen and the individual is killed. On the other hand, in fresh environment, ($\eta = 1$), the reproduction is successful and the individual is replaced by two offspring with phenotypes $\tau_i = \tilde{\tau}_i + \xi_i$ and $\tau_j = \tilde{\tau}_i + \xi_j$, where ξ_i, ξ_j are the variations, which are sampled from probability distribution $\beta(\xi_i = \tau_i - \tilde{\tau}_i; \tilde{\tau}_i)$ and $\beta(\xi_j = \tau_j - \tilde{\tau}_i; \tilde{\tau}_i)$ respectively. If the population size N has reached the carrying capacity K this reproduction

event must be accompanied by the death of individual j with trait τ_j, chosen at random (i.e. with rate $\mu = \frac{1}{K-1}$), much as in the Moran process. In principle, β is an arbitrary probability distribution normalized in the jump amplitude (the first argument): $\int_{-\tilde{\tau}}^{\infty} \beta(\tau - \tilde{\tau}; \tilde{\tau}) d(\tau - \tilde{\tau}) = 1$, but it can depend on the initial phenotype, that is indicated with the second argument. In the following, for the sake of notation, the second argument will be omitted when not necessary.

- If in the awake state, it can die by natural causes at rate $\mu(\tau_i, \boldsymbol{\tau}_N) = d \in \mathbb{R}^+$.

Furthermore, any individual with trait τ_i can switch on and off from dormancy with the following processes (Δ_{GD}):

- If dormant ($y_i = 1$), it can wake up at rate $A(\tau_i, \boldsymbol{\tau}_N) = 1/\tau_i$.
- If awake ($y_i = 1$) and in the presence of antibiotics ($\eta = -1$) it can sense their presence and switch to dormancy at rate $S(\tau_i, \boldsymbol{\tau}_N) = s \in \mathbb{R}^+$.

A. Master equation

The general trait-dependent expression of the rates will be used for the sake of theoretical comprehensiveness, and it will be reduced to the particular one used in the main text when necessary. From these processes it is possible to write down a master equation for probability of the system configurations $P(\boldsymbol{\tau}_N, \mathbf{y}_N, t)$:

$$\frac{dP(\boldsymbol{\tau}_N, \mathbf{y}_N, t)}{dt} = \Delta_D P(\boldsymbol{\tau}_N, \boldsymbol{y}_N, t) + \Delta_{GD} P(\boldsymbol{\tau}_N, \boldsymbol{y}_N, t), \tag{S2}$$

where Δ_{BD} stands for the time change with respect to the demographic part of the dynamics, and the Δ_{GD} for the *growing-dormant* state transitions. In addition, it is useful to separate the demographic change into two contributions, the *birth-death* part, i.e. the set of processes that change the population size N, and the *Moran* processes part that drives the dynamics when the carrying capacity K is reached and does not change N:

$$\frac{dP(\boldsymbol{\tau}_N, \boldsymbol{y}_N, t)}{dt} = (1 - \delta_{N,K}) \Delta_{BD} P(\boldsymbol{\tau}_N, \boldsymbol{y}_N, t) + \delta_{N,K} \Delta_M P(\boldsymbol{\tau}_N, \mathbf{y}_N, t) + \Delta_{GD} P(\boldsymbol{\tau}_N, \boldsymbol{y}_N, t). \tag{S3}$$

Birth-death contribution To take into account the change in population size, it is assumed that the probability of a state $(\boldsymbol{\tau}_N, \boldsymbol{y}_N, t)$ can be written as the ratio of some population state function $\psi(\boldsymbol{\tau}_N, \boldsymbol{y}_N, t)$, $\psi : \mathcal{P} \to \mathcal{R}^{0,+}$, and its normalization:

$$P(\boldsymbol{\tau}_N, \boldsymbol{y}_N, t) \equiv \frac{\psi(\boldsymbol{\tau}_N, \boldsymbol{y}_N, t)}{\int_{\mathcal{P}^N} d\boldsymbol{\tau}_N \sum_{\mathbf{y} \in \{\pm 1\}^N} \psi(\boldsymbol{\tau}_N, \boldsymbol{y}_N, t)}. \tag{S4}$$

ψ is non-negative, and its integral is heuristically assumed not to diverge, but it is not a probability function since it is not normalized. It can be thought as a scalar field generalizing, to population configurations, the concept of the number of individuals of a particular species in a discrete set of them (e.g. in evolutionary game theory, where the frequency of species i is written as $p_i = \frac{n_i}{N}$, where n_i is the number of individual of species i and N the population size). The time evolution of the probability density is specified by:

$$\begin{aligned}
\Delta_{BD} P(\boldsymbol{\tau}_N, \boldsymbol{y}_N, t) &= \frac{\dot{\psi}(\boldsymbol{\tau}_N, \boldsymbol{y}_N, t)}{\int_{\mathcal{P}^N} d\boldsymbol{\tau}_N \sum_{\mathbf{y} \in \{\pm 1\}^N} \psi(\boldsymbol{\tau}_N, \boldsymbol{y}_N, t)} - \frac{\psi(\boldsymbol{\tau}_N, \boldsymbol{y}_N, t)}{\int_{\mathcal{P}^N} d\boldsymbol{\tau}_N \sum_{\mathbf{y} \in \{\pm 1\}^N} \psi(\boldsymbol{\tau}_N, \boldsymbol{y}_N, t)} \frac{\int_{\mathcal{P}^N} d\boldsymbol{\tau}_N \sum_{\mathbf{y} \in \{\pm 1\}^N} \dot{\psi}(\boldsymbol{\tau}_N, \boldsymbol{y}_N, t)}{\int_{\mathcal{P}^N} d\boldsymbol{\tau}_N \sum_{\mathbf{y} \in \{\pm 1\}^N} \psi(\boldsymbol{\tau}_N, \boldsymbol{y}_N, t)} \\
&= \frac{\dot{\psi}(\boldsymbol{\tau}_N, \boldsymbol{y}_N, t)}{\int_{\mathcal{P}^N} \sum_{\mathbf{y} \in \{\pm 1\}^N} d\boldsymbol{\tau}_N \psi(\boldsymbol{\tau}_N, \boldsymbol{y}_N, t)} - P(\boldsymbol{\tau}_N, \boldsymbol{y}_N, t) \frac{\int_{\mathcal{P}^N} d\boldsymbol{\tau}_N \sum_{\mathbf{y} \in \{\pm 1\}^N} \dot{\psi}(\boldsymbol{\tau}_N, \boldsymbol{y}_N, t)}{\int_{\mathcal{P}^N} d\boldsymbol{\tau}_N \sum_{\mathbf{y} \in \{\pm 1\}^N} \psi(\boldsymbol{\tau}_N, \boldsymbol{y}_N, t)} \\
&= \frac{\dot{\psi}(\boldsymbol{\tau}_N, \boldsymbol{y}_N, t)}{\int_{\mathcal{P}^N} \sum_{\mathbf{y} \in \{\pm 1\}^N} d\boldsymbol{\tau}_N \psi(\boldsymbol{\tau}_N, \boldsymbol{y}_N, t)} - \Lambda_N(t) P(\boldsymbol{\tau}_N, \boldsymbol{y}_N, t),
\end{aligned} \tag{S5}$$

where we have defined the normalizing factor:

$$\Lambda_N(t) \equiv \frac{\int_{\mathcal{P}^N} d\boldsymbol{\tau}_N \sum_{\mathbf{y} \in \{\pm 1\}^N} \dot{\psi}(\boldsymbol{\tau}_N, \boldsymbol{y}_N, t)}{\int_{\mathcal{P}^N} d\boldsymbol{\tau}_N \sum_{\mathbf{y} \in \{\pm 1\}^N} \psi(\boldsymbol{\tau}_N, \boldsymbol{y}_N, t)}. \tag{S6}$$

Note that in (S5) the probability is conserved, so once the equation of motion for ψ is written down, one has an equation for P. Thus, for the Δ_{BD} term it is mandatory to take into account this normalization, whereas it is not necessary for the Δ_{GD} and Δ_M terms, which do not change N.

Let us consider the various processes that change the population size:

- Birth, that increases the population's size of one, e.g. from a state $\tilde{\tau}^{i,\hat{j}}_{N-1}$ to τ_N, with rate:

$$\mathcal{B}(\boldsymbol{\tau}_N, \boldsymbol{y}_N|\tilde{\boldsymbol{\tau}}^{i,\hat{j}}_{N-1}\tilde{\boldsymbol{y}}^{i,\hat{j}}_{N-1}) = \beta(\tau_i - \tilde{\tau}_i)\beta(\tau_j - \tilde{\tau}_i)g(\tilde{\tau}_i, \tilde{\boldsymbol{\tau}}^{i,\hat{j}}_{N-1})\delta_{\eta,1}\delta_{\tilde{y}_i,1}\delta_{y_i,1}\delta_{y_j,1}. \tag{S7}$$

  where the i individual with trait $\tilde{\tau}_i$ produces two offspring with traits τ_i, τ_j. Naturally, to reproduce individual i must be in the growing state and antibiotics must be absent.

- Death of the i individual by the effect of antibiotics, which reduces the population size from N to $N-1$ at rate:

$$\mathcal{Q}(\boldsymbol{\tau}^{\hat{i}}_{N-1}, \boldsymbol{y}^{\hat{i}}_{N-1}|\boldsymbol{\tau}_N, \boldsymbol{y}_N) = g(\tau_i, \boldsymbol{\tau}_N)\delta_{\eta,-1}\delta_{y_i,1}, \tag{S8}$$

  where the index $\hat{i}$ represent the absence of the i individual with respect to $\boldsymbol{\tau}_N$.

- Natural death of individual i at rate:

$$\mathcal{T}(\boldsymbol{\tau}^{\hat{i}}_{N-1}, \boldsymbol{y}^{\hat{i}}_{N-1}|\boldsymbol{\tau}_N, \boldsymbol{y}_N) = \mu(\tau_i, \boldsymbol{\tau_N})\delta_{y_i,1}. \tag{S9}$$

  Thus, by adding these two rates (eq.S8-S9) one gets the full death rate:

$$\mathcal{D}(\boldsymbol{\tau}^{\hat{i}}_{N-1}, \boldsymbol{y}^{\hat{i}}_{N-1}|\boldsymbol{\tau}_N, \boldsymbol{y}_N) \equiv \mathcal{Q}(\boldsymbol{\tau}^{\hat{i}}_{N-1}, \boldsymbol{y}^{\hat{i}}_{N-1}|\boldsymbol{\tau}_N, \tilde{\boldsymbol{y}}_N) + \mathcal{T}(\boldsymbol{\tau}^{\hat{i}}_{N-1}, \boldsymbol{y}^{\hat{i}}_{N-1}|\boldsymbol{\tau}_N, \boldsymbol{y}_N). \tag{S10}$$

Putting together all these contributions, Δ_{BD} can be written as:

$$\begin{aligned}
\Delta_{BD}P(\boldsymbol{\tau}_N, \boldsymbol{y}_N, t) = &\sum_{i=1}^{N}\sum_{j=1, j\neq i}^{N}\sum_{\tilde{y}_i\in\{\pm 1\}}\int_{\mathcal{P}} d\tilde{\tau}_i \frac{1}{N-1}\mathcal{B}(\boldsymbol{\tau}_N, \boldsymbol{y}_N|\tilde{\boldsymbol{\tau}}^{i,\hat{j}}_{N-1}\tilde{\boldsymbol{y}}^{i,\hat{j}}_{N-1})\frac{\psi(\tilde{\boldsymbol{\tau}}^{i,\hat{j}}_{N-1}, \tilde{\boldsymbol{y}}^{i,\hat{j}}_{N-1}, t)}{\int_{\mathcal{P}^N} d\boldsymbol{\tau}_N \sum_{\mathbf{y}\in\{\pm 1\}^N}\psi(\boldsymbol{\tau}_N, \boldsymbol{y}_N, t)} \\
&+ \sum_{i=1}^{N+1}\sum_{y_i\in\{\pm 1\}}\int_{\mathcal{P}} d\tau_i \frac{1}{N+1}\mathcal{D}(\boldsymbol{\tau}^{\hat{i}}_N, \boldsymbol{y}^{\hat{i}}_N|\boldsymbol{\tau}_{N+1}, \boldsymbol{y}_{N+1})\frac{\psi(\boldsymbol{\tau}_{N+1}, \boldsymbol{y}_{N+1}, t)}{\int_{\mathcal{P}^N} d\boldsymbol{\tau}_N \sum_{\mathbf{y}\in\{\pm 1\}^N}\psi(\boldsymbol{\tau}_N, \boldsymbol{y}_N, t)} \\
&- \sum_{i=1}^{N}\sum_{j=1, j\neq i}^{N+1}\sum_{\tilde{y}_i\in\{\pm 1\}}\int_{\mathcal{P}^2} d\tilde{\tau}_i d\tilde{\tau}_j \frac{1}{N}\mathcal{B}(\tilde{\boldsymbol{\tau}}^{i,j}_{N+1}, \tilde{\boldsymbol{y}}^{i,j}_{N+1}|\boldsymbol{\tau}_N, \boldsymbol{y}_N)\frac{\psi(\boldsymbol{\tau}_N, \boldsymbol{y}_N, t)}{\int_{\mathcal{P}^N} d\boldsymbol{\tau}_N \sum_{\mathbf{y}\in\{\pm 1\}^N}\psi(\boldsymbol{\tau}_N, \boldsymbol{y}_N, t)} \\
&- \sum_{i=1}^{N}\sum_{y_i\in\{\pm 1\}}\mathcal{D}(\boldsymbol{\tau}^{\hat{i}}_{N-1}, \boldsymbol{y}^{\hat{i}}_{N-1}|\boldsymbol{\tau}_N, \boldsymbol{y}_N)\frac{\psi(\boldsymbol{\tau}_N, \boldsymbol{y}_N, t)}{\int_{\mathcal{P}^N} d\boldsymbol{\tau}_N \sum_{\mathbf{y}\in\{\pm 1\}^N}\psi(\boldsymbol{\tau}_N, \boldsymbol{y}_N, t)} \\
&- P(\boldsymbol{\tau}_N, \boldsymbol{y}_N, \boldsymbol{y}_N, t)\Lambda_N(t).
\end{aligned} \tag{S11}$$

Note that in the first three lines it is necessary to normalise the sums by the initial population size to respect individuals' indistinguishability. Consider for example the first line: it quantifies the probabilities that the state $\boldsymbol{\tau}_N$ is the result of a birth event in the state $\tilde{\boldsymbol{\tau}}^{i,\hat{j}}_{N-1}$ where the i individual has reproduced. One of the offspring is indicated as x_i but there are $N-1$ possibilities of assigning the index j to the second one. Due to individual indistinguishability all these possibilities are equivalent, and therefore the sum must be normalized by $N-1$.

To rewrite this expression in terms of probabilities, we normalize it in the following way:

$$\frac{\psi(\boldsymbol{\tau}_N)}{\int_{\mathcal{P}^M} d\boldsymbol{\tau}_M \psi(\boldsymbol{\tau}_M, t)} = \frac{\psi(\boldsymbol{\tau}_N)}{\int_{\mathcal{P}^N} d\boldsymbol{\tau}_N \psi(\boldsymbol{\tau}_N, t)}\frac{\int_{\mathcal{P}^N} d\boldsymbol{\tau}_N \psi(\boldsymbol{\tau}_N, t)}{\int_{\mathcal{P}^M} d\boldsymbol{\tau}_M \psi(\boldsymbol{\tau}_M, t)} = P(\boldsymbol{\tau}_N)\frac{\int_{\mathcal{P}^N} d\boldsymbol{\tau}_N \psi(\boldsymbol{\tau}_N, t)}{\int_{\mathcal{P}^M} d\boldsymbol{\tau}_M \psi(\boldsymbol{\tau}_M, t)} \equiv P(\boldsymbol{\tau}_N)a_{N,M}, \tag{S12}$$

where the re-scaling factor is defined as:

$$a_{N,M} \equiv \frac{\int_{\mathcal{P}^N} d\boldsymbol{\tau}_N \psi(\boldsymbol{\tau}_N, t)}{\int_{\mathcal{P}^M} d\boldsymbol{\tau}_M \psi(\boldsymbol{\tau}_M, t)}. \tag{S13}$$

Thanks to this definition, Δ_{BD} reads:

$$\begin{aligned}\Delta_{BD}P(\boldsymbol{\tau}_N,\boldsymbol{y}_N,t) &= a_{N-1,N}\sum_{i=1}^{N}\sum_{j=1,i\neq j}^{N}\sum_{\tilde{y}_i\in\{\pm1\}}\int_{\mathcal{P}}d\tilde{\tau}_i\frac{1}{N-1}\mathcal{B}(\boldsymbol{\tau}_N,\boldsymbol{y}_N|\tilde{\boldsymbol{\tau}}^{i,\hat{j}}_{N-1}\tilde{\boldsymbol{y}}^{i,\hat{j}}_{N-1})P(\tilde{\boldsymbol{\tau}}^{i,\hat{j}}_{N-1},\tilde{\boldsymbol{y}}^{i,\hat{j}}_{N-1})\\
&+ a_{N+1,N}\sum_{i=1}^{N+1}\sum_{y_i\in\{\pm1\}}\int_{\mathcal{P}}d\tau_i\frac{1}{N+1}\mathcal{D}(\boldsymbol{\tau}^{\hat{i}}_N,\boldsymbol{y}^{\hat{i}}_N|\boldsymbol{\tau}_{N+1},\boldsymbol{y}_{N+1})P(\boldsymbol{\tau}_{N+1},\boldsymbol{y}_{N+1},t)\\
&-\sum_{i=1}^{N}\sum_{j=1\neq i}^{N+1}\sum_{\tilde{y}_i\in\{\pm1\}}\int_{\mathcal{P}^2}d\tilde{\tau}_i d\tilde{\tau}_j\frac{1}{N}\mathcal{B}(\tilde{\boldsymbol{\tau}}^{i,j}_{N+1},\tilde{\boldsymbol{y}}^{i,j}_{N+1}|\boldsymbol{\tau}_N,\boldsymbol{y}_N)P(\boldsymbol{\tau}_N,\boldsymbol{y}_N,t)\\
&-\sum_{i=1}^{N}\sum_{y_i\in\{\pm1\}}\mathcal{D}(\boldsymbol{\tau}^{\hat{i}}_{N-1},\boldsymbol{y}^{\hat{i}}_{N-1}|\boldsymbol{\tau}_N,\boldsymbol{y}_N)P(\boldsymbol{\tau}_N,\boldsymbol{y}_N,t)\\
&-\Lambda_N(t)P(\boldsymbol{\tau}_N,\boldsymbol{y}_N,\boldsymbol{y}_N,t). \qquad \text{(S14)}\end{aligned}$$

Let us now calculate explicitly the normalization factor $\Lambda_N(t)$:

$$\begin{aligned}\Lambda_N(t) &= \frac{\int_{\mathcal{P}^N}d\boldsymbol{\tau}_N\sum_{\mathbf{y}\in\{\pm1\}}\dot{\psi}(\boldsymbol{\tau}_N,\boldsymbol{y}_N,t)}{\int_{\mathcal{P}^N}d\boldsymbol{\tau}_N\sum_{\mathbf{y}\in\{\pm1\}}\psi(\boldsymbol{\tau}_N,\boldsymbol{y}_N,t)} =\\
&= a_{N-1,N}\sum_{\mathbf{y}\in\{\pm1\}}\int_{\mathcal{P}^N}d\boldsymbol{\tau}_N\sum_{i=1}^{N-1}\sum_{j=1,i\neq j}^{N}\sum_{\tilde{y}_i\in\{\pm1\}}\int_{\mathcal{P}}d\tilde{\tau}_i\frac{1}{N-1}\mathcal{B}(\boldsymbol{\tau}_N,\boldsymbol{y}_N|\tilde{\boldsymbol{\tau}}^{i,\hat{j}}_{N-1}\tilde{\boldsymbol{y}}^{i,\hat{j}}_{N-1})P(\tilde{\boldsymbol{\tau}}^{i,\hat{j}}_{N-1},\tilde{\boldsymbol{y}}^{i,\hat{j}}_{N-1})\\
&+ a_{N+1,N}\sum_{\mathbf{y}\in\{\pm1\}}\int_{\mathcal{P}^N}d\boldsymbol{\tau}_N\sum_{i=1}^{N+1}\sum_{y_i\in\{\pm1\}}\int_{\mathcal{P}}d\tau_i\frac{1}{N+1}\mathcal{D}(\boldsymbol{\tau}^{\hat{i}}_N,\boldsymbol{y}^{\hat{i}}_N|\boldsymbol{\tau}_{N+1},\boldsymbol{y}_{N+1})P(\boldsymbol{\tau}_{N+1},\boldsymbol{y}_{N+1},t)\\
&-\sum_{\mathbf{y}\in\{\pm1\}}\int_{\mathcal{P}^N}d\boldsymbol{\tau}_N\sum_{i=1}^{N}\sum_{j=1\neq i}^{N+1}\sum_{\tilde{y}_i=\pm1}\int_{\mathcal{P}^2}d\tilde{\tau}_i d\tilde{\tau}_j\frac{1}{N}\mathcal{B}(\tilde{\boldsymbol{\tau}}^{i,j}_{N+1},\tilde{\boldsymbol{y}}^{i,j}_{N+1}|\boldsymbol{\tau}_N,\boldsymbol{y}_N)P(\boldsymbol{\tau}_N,\boldsymbol{y}_N,t)\\
&-\sum_{\mathbf{y}\in\{\pm1\}}\int_{\mathcal{P}^N}d\boldsymbol{\tau}_N\sum_{i=1}^{N}\sum_{\tilde{y}_i\in\{\pm1\}}\mathcal{D}(\boldsymbol{\tau}^{\hat{i}}_{N-1},\boldsymbol{y}^{\hat{i}}_{N-1}|\boldsymbol{\tau}_N,\boldsymbol{y}_N)P(\boldsymbol{\tau}_N,\boldsymbol{y}_N,t). \qquad \text{(S15)}\end{aligned}$$

The expression can be split in four terms:

$$\Lambda_N(t) = \Lambda_1+\Lambda_2-\Lambda_3-\Lambda_4, \qquad \text{(S16)}$$

and each of them can be calculated separately. The first term is:

$$\begin{aligned}\Lambda_1 &= a_{N-1,N}\sum_{\mathbf{y}\in\{\pm1\}}\int_{\mathcal{P}^N}d\boldsymbol{\tau}_N\sum_{i=1}^{N}\sum_{j=1,j\neq i}^{N}\sum_{\tilde{y}_i\in\{\pm1\}}\int_{\mathcal{P}}d\tilde{\tau}_i\frac{1}{N-1}\mathcal{B}(\boldsymbol{\tau}_N,\boldsymbol{y}_N|\tilde{\boldsymbol{\tau}}^{i,\hat{j}}_{N-1}\tilde{\boldsymbol{y}}^{i,\hat{j}}_{N-1})P(\tilde{\boldsymbol{\tau}}^{i,\hat{j}}_{N-1},\tilde{\boldsymbol{y}}^{i,\hat{j}}_{N-1})\\
&= a_{N-1,N}\sum_{\mathbf{y}\in\{\pm1\}}\int_{\mathcal{P}^N}d\boldsymbol{\tau}_N\sum_{i=1}^{N}\sum_{j=1,j\neq i}^{N}\sum_{\tilde{y}_i=\pm1}\int_{\mathcal{P}}d\tilde{\tau}_i\frac{1}{N-1}\beta(\tau_i-\tilde{\tau}_i)\beta(\tau_j-\tilde{\tau}_i)g(\tilde{\tau}_i,\tilde{\boldsymbol{\tau}}^{i,\hat{j}}_{N-1})\delta_{\eta,1}\delta_{\tilde{y}_i,1}\delta_{y_i,1}\delta_{y_j,1}P(\tilde{\boldsymbol{\tau}}^{i,\hat{j}}_{N-1},\tilde{\boldsymbol{y}}^{i,\hat{j}}_{N-1}).\end{aligned}$$

(S17)

Using the fact that individuals are indistinguishable the sums $\sum_{i=1}^{N}$, $\sum_{j=1,i\neq j}^{N}$ can be replaced with a factor $N\cdot(N-1)$ by fixing i and j, e.g. $i=1, j=2$:

$$\begin{aligned}\Lambda_1 &= N\cdot a_{N-1,N}\sum_{\boldsymbol{y}\in\{\pm1\}}\int_{\mathcal{P}^N}d\boldsymbol{\tau}_N\sum_{\tilde{y}_1\in\{\pm1\}}\int_{\mathcal{P}}d\tilde{\tau}_1\beta(\tau_1-\tilde{\tau}_1)\beta(\tau_2-\tilde{\tau}_1)g(\tilde{\tau}_1,\tilde{\boldsymbol{\tau}}^{1,\hat{2}}_{N-1})\delta_{\eta,1}\delta_{\tilde{y}_1,1}\delta_{y_1,1}\delta_{y_2,1}P(\tilde{\boldsymbol{\tau}}^{1,\hat{2}}_N,\tilde{\boldsymbol{y}}^{1,\hat{2}}_N)\\
&= N\cdot a_{N-1,N}\sum_{\boldsymbol{y}\in\{\pm1\}}\int_{\mathcal{P}^{N-1}}d\tilde{\boldsymbol{\tau}}^{1,\hat{2}}_{N-1}\sum_{\tilde{y}_1=\pm1}\int_{\mathcal{P}}d\tau_1\beta(\tau_1-\tilde{\tau}_1)\int_{\mathcal{P}}d\tau_2\beta(\tau_2-\tilde{\tau}_1)g(\tilde{\tau}_1,\tilde{\boldsymbol{\tau}}^{1,\hat{2}}_{N-1})\delta_{\eta,1}\delta_{\tilde{y}_1,1}\delta_{y_1,1}\delta_{y_2,1}P(\tilde{\boldsymbol{\tau}}^{1,\hat{2}}_N,\tilde{\boldsymbol{y}}^{1,\hat{2}}_N).\end{aligned}$$

(S18)

By using the normalization of the β's in the variable $\delta = \tau - \tilde{\tau}$, $\int_0^\infty d\tau_1 \beta(\tau_1 - \tilde{\tau}_1) = \int_{-\tilde{\tau}_1}^\infty d\delta \beta(\delta) = \int_0^\infty d\tau_2 \beta(\tau_2 - \tilde{\tau}_1) = 1$, one obtains:

$$\Lambda_1 = N \cdot a_{N-1,N} \sum_{\boldsymbol{y} \in \{\pm 1\}} \int_{\mathcal{P}^{N-1}} d\tilde{\boldsymbol{\tau}}_{N-1}^{1,\hat{2}} \sum_{\tilde{y}_1 \in \{\pm 1\}} g(\tilde{\tau}_1, \tilde{\boldsymbol{\tau}}_{N-1}^{1,\hat{2}}) \delta_{\eta,1} \delta_{\tilde{y}_1,1} \delta_{y_1,1} \delta_{y_2,1} P(\tilde{\boldsymbol{\tau}}_N^{1,\hat{2}}, \tilde{\boldsymbol{y}}_N^{1,\hat{2}}). \tag{S19}$$

By performing a marginalization $\sum_{\boldsymbol{y} \in \{\pm 1\}} P(\tilde{\boldsymbol{\tau}}_N^{1,\hat{2}}, \tilde{\boldsymbol{y}}_N^{1,\hat{2}}) = P(\tilde{\boldsymbol{\tau}}_N^{1,\hat{2}}, \tilde{y}_1)$ and defining the average birth and death rates:

$$\bar{g}_N = \int_{\mathcal{P}^N} d\boldsymbol{\tau}_N g(\tau_i, \boldsymbol{\tau}_N) P(\boldsymbol{\tau}_N, y_i, t) \delta_{y_i,1}, \tag{S20}$$

$$\bar{\mu}_N = \int_{\mathcal{P}^N} d\boldsymbol{\tau}_N \mu(\tau_i, \boldsymbol{\tau}_N) P(\boldsymbol{\tau}_N, y_i, t) \delta_{y_i,1}, \tag{S21}$$

one readily obtains:

$$\Lambda_1 = N \cdot a_{N-1,N} \sum_{\mathbf{y} \in \{\pm 1\}} \int_{\mathcal{P}^{N-1}} d\tilde{\boldsymbol{\tau}}_{N-1}^{1,\hat{2}} \sum_{\tilde{y}_1 \in \{\pm 1\}} g(\tilde{\tau}_1, \tilde{\boldsymbol{\tau}}_{N-1}^{1,\hat{2}}) \delta_{\eta,1} \delta_{\tilde{y}_1,1} \delta_{y_1,1} \delta_{y_2,1} P(\tilde{\boldsymbol{\tau}}_N^{1,\hat{2}}, \tilde{\boldsymbol{y}}_N^{1,\hat{2}}) = a_{N-1,N} N \bar{g}_{N-1} \delta_{\eta,1} \tag{S22}$$

Similarly, one can work out the second term:

$$\begin{aligned}
\Lambda_2 &= a_{N+1,N} \sum_{\mathbf{y} \in \{\pm 1\}} \int_{\mathcal{P}^N} d\boldsymbol{\tau}_N \sum_{i=1}^{N+1} \sum_{y_i \in \{\pm 1\}} \int_{\mathcal{P}} d\tau_i \frac{1}{N+1} \mathcal{D}(\boldsymbol{\tau}_N^{\hat{i}}, \boldsymbol{y}_N^{\hat{i}} | \boldsymbol{\tau}_{N+1}, \boldsymbol{y}_{N+1}) P(\boldsymbol{\tau}_{N+1}, \boldsymbol{y}_{N+1}, t) \\
&= a_{N+1,N} \sum_{\mathbf{y} \in \{\pm 1\}} \int_{\mathcal{P}^N} d\boldsymbol{\tau}_N \sum_{y_{N+1} \in \{\pm 1\}} \int_{\mathcal{P}} d\tau_{N+1} P(\boldsymbol{\tau}_{N+1}, \boldsymbol{y}_{N+1}, t) \left[\mu(\tau_{N+1}, \boldsymbol{\tau}_{\boldsymbol{N+1}}) \delta_{y_{N+1},1} + g(\tau_{N+1}, \boldsymbol{\tau}_{N+1}) \delta_{\eta,-1} \delta_{y_{N+1},1} \right] = \\
&= a_{N+1,N} (\bar{\mu}_{N+1} + \bar{g}_{N+1} \delta_{\eta,-1}),
\end{aligned} \tag{S23}$$

where i has been fixed to $N+1$. The third one reads:

$$\Lambda_3 = \sum_{\mathbf{y} \in \{\pm 1\}} \int_{\mathcal{P}^N} d\boldsymbol{\tau}_N \sum_{i=1}^{N} \sum_{j=1 \neq i}^{N+1} \sum_{\tilde{y}_i \in \{\pm 1\}} \int_{\mathcal{P}^2} d\tilde{\tau}_i d\tilde{\tau}_j \frac{1}{N} \mathcal{B}(\tilde{\boldsymbol{\tau}}_{N+1}^{i,j}, \tilde{\boldsymbol{y}}_{N+1}^{i,j} | \boldsymbol{\tau}_N, \boldsymbol{y}_N) P(\boldsymbol{\tau}_N, \boldsymbol{y}_N, t) \tag{S24}$$

$$\begin{aligned}
&= \sum_{\mathbf{y} \in \{\pm 1\}} \int_{\mathcal{P}^N} d\boldsymbol{\tau}_N \sum_{i=1}^{N} \sum_{\tilde{y}_i \in \{\pm 1\}} \int_{\mathcal{P}} d\tilde{\tau}_i \beta(\tau_i - \tilde{\tau}_{N+1}) \beta(\tau_{N+1} - \tilde{\tau}_i) g(\tilde{\tau}_i, \tilde{\boldsymbol{\tau}}_N^{i,\hat{j}}) \delta_{\eta,1} \delta_{\tilde{y}_i,1} \delta_{y_i,1} \delta_{y_{N+1},1} P(\tilde{\boldsymbol{\tau}}_N^{i,\widehat{N+1}}, \tilde{\boldsymbol{y}}_N^{i,\widehat{N+1}}) = \\
&= N \cdot \int_{\mathcal{P}^{N-1}} d\tilde{\boldsymbol{\tau}}_N^1 \sum_{\tilde{y}_1 \in \{\pm 1\}} \int_{\mathcal{P}} d\tau_1 \beta(\tau_1 - \tilde{\tau}_1) \int_P d\tau_{N+1} \beta(\tau_{N+1} - \tilde{\tau}_1) g(\tilde{\tau}_1, \tilde{\boldsymbol{\tau}}_N^1) P(\tilde{\boldsymbol{\tau}}_N^1, \tilde{\boldsymbol{y}}_N^1) \delta_{\eta,1} \delta_{\tilde{y}_1,1} \delta_{y_1,1} = \\
&= N \cdot \int_{\mathcal{P}^{N-1}} d\tilde{\boldsymbol{\tau}}_N^1 \sum_{\tilde{y}_1 \in \{\pm 1\}} g(\tilde{\tau}_1, \tilde{\boldsymbol{\tau}}_N^1) P(\tilde{\boldsymbol{\tau}}_N^1, \tilde{\boldsymbol{y}}_N^1) \delta_{\eta,1} \delta_{\tilde{y}_1,1} \delta_{y_1,1} = N \bar{g}_N \delta_{\eta,1},
\end{aligned} \tag{S25}$$

where the indices has been fixed as $i = 1, j = N+1$. Finally, the fourth one term can be written as:

$$\begin{aligned}
\Lambda_4 &= \sum_{\mathbf{y} \in \{\pm 1\}} \int_{\mathcal{P}^N} d\boldsymbol{\tau}_N \sum_{i=1}^{N} \sum_{y_i \in \{\pm 1\}} \mathcal{D}(\boldsymbol{\tau}_{N-1}^{\hat{i}}, \boldsymbol{y}_{N-1}^{\hat{i}} | \boldsymbol{\tau}_N, \boldsymbol{y}_N) P(\boldsymbol{\tau}_N, \boldsymbol{y}_N, t) = \\
&= \sum_{\mathbf{y} \in \{\pm 1\}} \int_{\mathcal{P}^N} d\boldsymbol{\tau}_N \sum_{i=1}^{N} \sum_{y_i \in \{\pm 1\}} P(\boldsymbol{\tau}_N, \boldsymbol{y}_N, t) \left[\mu(\tau_i, \boldsymbol{\tau}_{\boldsymbol{N}}) \delta_{y_i,1} + g(\tau_i, \boldsymbol{\tau}_N) \delta_{\eta,-1} \delta_{y_i,1} \right] = \\
&= N \sum_{\mathbf{y} \in \{\pm 1\}} \int_{\mathcal{P}^N} d\boldsymbol{\tau}_N \sum_{y_1 \in \{\pm 1\}} P(\boldsymbol{\tau}_N, \boldsymbol{y}_N, t) \left[\mu(\tau_1, \boldsymbol{\tau}_{\boldsymbol{N}}) \delta_{y_1,1} + g(\tau_1, \boldsymbol{\tau}_N) \delta_{\eta,-1} \delta_{y_1,1} \right] = N(\bar{\mu}_N + \bar{g}_N \delta_{\eta,-1}).
\end{aligned} \tag{S26}$$

Thus, by collecting the four terms (S22, S23, S24, S26) one finally obtains an explicit expression for the normalization factor:

$$\Lambda_N(t) = N \left[a_{N-1,N} \cdot \bar{g}_{N-1} - \bar{g}_N \right] \delta_{\eta,1} + (a_{N+1,N} \cdot \bar{\mu}_{N+1} - N \cdot \mu_N) + \left[a_{N+1,N} \bar{g}_{N+1} - N \cdot \bar{g}_N \right] \delta_{\eta,-1}. \tag{S27}$$

Moran process contribution. When the systems reaches the carrying capacity K each birth must be compensated by a random death. This part of the dynamics follows a master equation that conserves the system size $N = K$:

$$\Delta_M P(\boldsymbol{\tau}_n, \boldsymbol{y}_N, t) = \sum_{i=1}^{N} \sum_{j=1, j\neq i}^{N} \sum_{\tilde{y}_i \in \{\pm 1\}} \sum_{\tilde{y}_j \in \{\pm 1\}} \int_{\mathcal{P}^2} d\tilde{\tau}_i d\tilde{\tau}_j \Big[W_M(\boldsymbol{\tau}_N, \boldsymbol{y}_N | \tilde{\boldsymbol{\tau}}_N^{ij}, \tilde{\boldsymbol{y}}_N^{ij}) P(\tilde{\boldsymbol{\tau}}_N^{ij}, \tilde{\boldsymbol{y}}_N^{ij}, t) - W_M(\tilde{\boldsymbol{\tau}}_N^{ij}, \tilde{\boldsymbol{y}}_N^{ij} | \boldsymbol{\tau}_N, \boldsymbol{y}_N) P(\boldsymbol{\tau}_N, \boldsymbol{y}_N, t) \Big], \tag{S28}$$

where the transition rate is given by:

$$W_M(\boldsymbol{\tau}_N, \boldsymbol{y}_N | \tilde{\boldsymbol{\tau}}_N^{ij}, \tilde{\boldsymbol{y}}_N^{ij}) = \frac{1}{K-1} \beta(\tau_i - \tilde{\tau}_i) \beta(\tau_j - \tilde{\tau}_i) g(\tilde{\tau}_i, \tilde{\boldsymbol{\tau}}^{i,j}) \delta_{\eta,1} \delta_{\tilde{y}_i,1} \delta_{y_i,1} \delta_{y_j,1}. \tag{S29}$$

Growing and Dormant state contribution The part representing the transitions between growing and dormant states is relatively easy to define given that N is conserved:

$$\Delta_{GD} P(\boldsymbol{\tau}_N, \mathbf{y}_N, t) = \sum_{i=1}^{N} \sum_{\tilde{y}_i \in \{\pm 1\}} \Big[W_{GD}(\boldsymbol{\tau}_N, \mathbf{y}_N |, \boldsymbol{\tau}_N, \tilde{\mathbf{y}}_N^i) P(\boldsymbol{\tau}_N, \tilde{\mathbf{y}}_N^i, t) - W_{GD}(\boldsymbol{\tau}_N, \tilde{\mathbf{y}}_N^i |, \boldsymbol{\tau}_N, \mathbf{y}_N) P(\boldsymbol{\tau}_N, \mathbf{y}_N, t) \Big], \tag{S30}$$

where the transition rate $\tilde{y}_i \to y_i$ is given by:

$$W_{GD}(\boldsymbol{\tau}_N, \mathbf{y}_N | \boldsymbol{\tau}_N, \tilde{\mathbf{y}}_N^i) = \delta_{\tilde{y}_i,-1} \delta_{y_i,1} A(\tau_i, \boldsymbol{\tau}_N) + \delta_{\tilde{y}_i,1} \delta_{y_i,-1} \delta_{\eta,-1} S(\tau_i, \boldsymbol{\tau}_N), \tag{S31}$$

the δ stand for Kronecker deltas introduced because each term only contributes in a specific medium or state.

Thus, summarizing, the master equation defining our proposed model reads:

$$\frac{dP(\boldsymbol{\tau}_N, \boldsymbol{y}_N, t)}{dt} = (1 - \delta_{N,K}) \Delta_{BD} P(\boldsymbol{\tau}_N, \boldsymbol{y}_N, t) + \delta_{N,K} \Delta_M P(\boldsymbol{\tau}_N, \mathbf{y}_N, t) + \Delta_{GD} P(\boldsymbol{\tau}_N, \boldsymbol{y}_N, t), \tag{S32}$$

where the birth-death contribution Δ_{BD} is given by eqs.(S11,S27,S7,S10), the Moran process contribution Δ_M by eqs.(S28,S29) and the growing-dormant transition $\Delta_{GD}$is specified by eqs. (S30,S31).

B. Gillespie algorithm

To integrate the master equation (S2) numerically we use the Gillespie algorithm. The Gillespie algorithm is a kinetic Monte Carlo Method [7] initially proposed in the field of chemistry to simulate individual trajectories of a set of chemical reactions [3]. However, since its initial formulation, the Gillespie algorithm has gained great importance e.g. statistical physics and biophysics [4]. In general each individual simulation of the Gillespie algorithm represents an exact trajectory of the probability density function that is ruled by the master eq. (S2) [7].

At each time step one possible event is randomly chosen with a probability proportional to its rate. In our case there are four different events in the antibiotic-exposure phase and five events in the fresh medium phase. Moreover, each bacteria can be in two different states: growing state and dormant state.

Antibiotic-exposure phase: 1) A bacteria in the growing state can be randomly chosen to die with rate $d = const.$ Overall, bacteria death at rate $\varphi_{death} = N_G \cdot d$, where N_G is the number of growing individuals. 2) A bacteria in growing state can be randomly chosen to die while trying to reproduce with rate $b = const.$ Overall, bacteria are killed by antibiotics with rate $\varphi_{killed\ by\ a.} = b \cdot N_G$. 3) A bacteria in the growing state can sense the environment and enter in dormant state at rate $s = const.$ Overall, bacteria enter in dormant sate at rate $\varphi_{enter\ d.s.} = s \cdot N_G$. 4) A bacteria in the dormant state can randomly chosen to exit dormant state at rate $\varphi^i_{exit\ d.s.} = \frac{1}{\tau^i_{lag}}$. Overall, bacteria exit from dormant state at rate: $\sum_i \varphi^i_{exit\ d.s.}$.

Fresh medium phase: 1) A bacteria in the growing state can be randomly chosen for die at a constant rate d. Overall, bacteria death at rate φ_{death}. 2) A bacteria in the growing state can be randomly chosen to reproduce at constant rate b. Overall, bacteria reproduce at rate $\varphi_{birth} = b \cdot N_G$. Bacteria reproduce asexually by duplication, so both offspring will inherit the same phenotype τ^i_{lag} plus a variation. Here we explore two possibilities for the variation

$\sigma(\tau)$, additive amplitude $\sigma_A(\tau) \sim \alpha_A = const$ and multiplicative amplitude $\sigma_m(\tau) \sim \alpha_M \cdot \tau$, as defined in S3. 3)A bacteria in dormant state can randomly chosen to exit dormant state with rate $\varphi^i_{exit\ d.s.} = \frac{1}{\tau^i_{lag}}$. Overall, bacteria exit from dormant state at rate: $\sum_i \varphi^i_{exit\ d.s.}$. Importantly, there is also a maximum carrying capacity K, so once it is reached for each reproduction event , a randomly chosen member of the population is deleted (following a Moran process).

Since the rate $\varphi^i_{exit\ d.s.}$ is specific to each bacteria i, the search of the randomly chosen reaction could be computationally expensive, thus a binary search is implemented. In the worst case, binary search needs $O\left(Ln\left(N\right)\right)$ comparisons, whereas direct search needs $O\left(N\right)$ comparisons.

S2. FROM MICROSCOPIC TO MACROSCOPIC PROCESS

A. Marginalization

The master equation (S2,S14, S28, S30) rules the system's evolution at the microscopic level (individual-based). Our aim now is to derive the "one-cell" probability density [2], $\phi^{(1)}(\tau, y, t)$: given a certain pair (τ, y), $\phi^{(1)}$ gives the probability of finding an individual in that state at a time t, i.e. it is the one-particle probability density in the jargon of statistical physics. $\phi^{(1)}$ is an experimentally measurable magnitude that provides a macroscopic description (population-based) of the system.
To obtain the one-cell density one needs to marginalize $P(\boldsymbol{\tau}_N, \boldsymbol{y}_N, t)$, i.e. to remove the dependence of $\boldsymbol{P}$ in all individuals but one [5]. Mathematically, the one-cell probability density is defined as:

$$\phi^{(1)}(\tau, y, t) \equiv \frac{1}{N}\Big\langle \sum_{i=1}^{N} \sum_{\boldsymbol{y}_i \in \{\pm 1\}} \delta(\tau - \tau_i)\delta_{y,y_i} \Big\rangle, \tag{S33}$$

and, by writing down explicitly the average operator $\langle \dots \rangle$, it does coincide with the marginalized phenotypic distribution for the first individual:

$$\begin{aligned} \phi^{(1)}(\tau, y, t) &\equiv \frac{1}{N}\langle \sum_{i=1}^{N}\sum_{\boldsymbol{y}_i} \delta(\tau - \tau_i)\delta_{y,y_i}\rangle = \frac{1}{N}\sum_{\boldsymbol{y}\in\{\pm 1\}} \int_{\mathcal{P}^N} d\boldsymbol{\tau}_N \sum_{i=1}^{N} \delta(\tau - \tau_i)\delta_{y,y_i} P(\boldsymbol{\tau}, \boldsymbol{y}, t) = \\ &= \sum_{y_j \in\{\pm 1\}, j\in[2,N]} \int_{\mathcal{P}^{N-1}} d\tau_2 \dots d\tau_N P(\tau, \tau_2, \dots, \tau_N, y, y_2, y_3, .., y_N) = P^{(1)}(\tau, y, t). \end{aligned} \tag{S34}$$

In the same way the $n-$cells probability density can be defined:

$$\begin{aligned} \phi^{(n)}(\boldsymbol{\tau}_n, \boldsymbol{y}_n, t) &= \frac{N-n}{N!}\Big\langle \prod_{j=1}^{n}\sum_{i_j=1}^{N}\sum_{\boldsymbol{y}_N} \delta(\tau_j - \tau_{i_j})\delta_{y_j, y_i} \Big\rangle \\ &= P^n(\tau_1, \tau_2, .., \tau_n, y_1, y_2, .., y_n, t). \end{aligned} \tag{S35}$$

Taking the time derivative of $\phi^{(1)}$, by using the master eq.(S2) one obtains:

$$\begin{aligned} \partial_t \phi^{(1)}(\tau, y, t) &= \sum_{y_j\in\{\pm 1\}, j\in[2,N]} \int_{\mathcal{P}^{N-1}} d\tau_2 d\tau_3 .. d\tau_N \partial_t P(\boldsymbol{\tau}_N, \mathbf{y}_N, t) = \\ &= \sum_{y_j\in\{\pm 1\}, j\in[2,N]} \int_{\mathcal{P}^{N-1}} d\tau_2 d\tau_3 .. d\tau_N \Big[\Delta_D P(\boldsymbol{\tau}_N, \mathbf{y}_N, t) + \Delta_{GD} P(\boldsymbol{\tau}_N, \mathbf{y}_N, t)\Big] = \\ &= \Delta_{BD}\phi^{(1)}(\tau, y, t) + \Delta_M \phi^{(1)}(\tau, y, t) + \Delta_{GD}\phi^{(1)}(\tau, y, t). \end{aligned} \tag{S36}$$

Here on, we consider separately the birth-death (Δ_{BD}, $S14$), Moran (Δ_M, $S28$) and growing-dormant (Δ_{GD}, $S31$) contributions.

Birth and death contribution. The Birth-Death is defined as:

$$\Delta_{BD}\phi^{(1)}(\tau,y,t) = \sum_{y_k\in\{\pm1\},k\in[2,N]} \int_{\mathcal{P}^{N-1}} d\tau_2\dots d\tau_N \Delta_{BD} P(\boldsymbol{\tau}_N,\mathbf{y}_N,t). \tag{S37}$$

The birth-death contribution of the master eq.(S14) is composed by five terms: the four positive/negative contribution of birth and death, plus the normalizing factor. This last one does not depend on the variables, and thus it is not relevant for the marginalization. Therefore, we focus here on the first four terms one at the time:

$$\Delta_{BD}\phi^{(1)}(\tau,y,t) = B_1 + B_2 - B_3 - B_4 - \Lambda_N\phi^{(1)}(\tau,y,t) \tag{S38}$$

The first one takes into account the positive contribution of reproduction:

$$B_1 = a_{N-1,N} \sum_{y_k\in\{\pm1\},k\in[2,N]} \int_{\mathcal{P}^N} d\boldsymbol{\tau}^{\hat{1}}_{N-1} \sum_{i=1}^{N}\sum_{j=1,i\neq j}^{N}\sum_{\tilde{y}_i\in\{\pm1\}} \int_{\mathcal{P}} d\tilde{\tau}_i \frac{1}{N-1}\mathcal{B}(\boldsymbol{\tau}_N,\boldsymbol{y}_N|\tilde{\boldsymbol{\tau}}^{i,\hat{j}}_{N-1}\tilde{\boldsymbol{y}}^{i,\hat{j}}_{N-1})P(\tilde{\boldsymbol{\tau}}^{i,\hat{j}}_{N-1},\tilde{\boldsymbol{y}}^{i,\hat{j}}_{N-1},t). \tag{S39}$$

Let us split the i,j sums in three contributions: $\sum_{i=1}^N\sum_{j=1,i\neq j}^N = \delta_{i,1}\sum_{j=2}^N + \delta_{j,1}\sum_{i=2}^N + \sum_{i=2}^N\sum_{j=2\neq i}^N$, to decompose the expression in three contributions:

$$B_1 = \left(B_1^a + B_1^b + B_1^c\right) a_{N-1,N} \tag{S40}$$

The first two terms, B_1^a y B_1^b give a similar contribution: in the first sum, the individual 1 duplicates and gives birth to the new individual 1 and the j one, while the second term is just the reversed process. Indeed, there are $(N-1)$ ways of doing so, because when the first individual is chosen there are other $N-1$ that could be its offspring. Hence, the first term is then computed as follows:

$$B_1^a = \sum_{y_k\in\{\pm1\},k\in[2,N]} \int_{\mathcal{P}^{N-1}} d\boldsymbol{\tau}^{\hat{1}}_{N-1}\delta_{i,1}\sum_{j=2}^{N}\sum_{\tilde{y}_i\in\{\pm1\}}\int_P d\tilde{\tau}_i \frac{P(\tilde{\boldsymbol{\tau}}^{i,\hat{j}}_{N-1},\tilde{\boldsymbol{y}}^{i,\hat{j}}_{N-1},t)}{N-1}\beta(\tau_i-\tilde{\tau}_i)\beta(\tau_j-\tilde{\tau}_i)g(\tilde{\tau}_i,\tilde{\boldsymbol{\tau}}^{i,\hat{j}}_{N-1})\delta_{\eta,1}\delta_{\tilde{y}_i,1}\delta_{y_i,1}\delta_{y_j,1}, \tag{S41}$$

and using the fact that individuals are indistinguishable, one can rewrite the $\sum_{j=2}^N$ by fixing $j=2$ and pulling out a $N-1$ factor:

$$\begin{aligned} B_1^a &= \sum_{y_k\in\{\pm1\},k\in[2,N]} \int_{\mathcal{P}^N} d\tilde{\boldsymbol{\tau}}^1_N \sum_{\tilde{y}_1\in\{\pm1\}} P(\tilde{\boldsymbol{\tau}}^{1,\hat{2}}_{N-1},\tilde{\boldsymbol{y}}^{1,\hat{2}}_{N-1},t)\beta(\tau_1-\tilde{\tau}_1)\beta(\tau_2-\tilde{\tau}_1)g(\tilde{\tau}_1,\tilde{\boldsymbol{\tau}}^{1,\hat{2}}_{N-1})\delta_{\eta,1}\delta_{\tilde{y}_1,1}\delta_{y_1,1}\delta_{y_2,1} \\ &= \sum_{y_k\in\{\pm1\},k\in[2,N]} \int_{\mathcal{P}^{N-1}} d\tilde{\boldsymbol{\tau}}^{1,\hat{2}}_{N-1} \sum_{\tilde{y}_1\in\{\pm1\}} P(\tilde{\boldsymbol{\tau}}^{1,\hat{2}}_{N-1},\tilde{\boldsymbol{y}}^{1,\hat{2}}_{N-1},t)g(\tilde{\tau}_1,\tilde{\boldsymbol{\tau}}^{1,\hat{2}}_{N-1})\beta(\tau_1-\tilde{\tau}_1)\delta_{\eta,1}\delta_{\tilde{y}_1,1}\delta_{y_1,1}\delta_{y_2,1}. \end{aligned} \tag{S42}$$

The second contribution of the sum has a very similar form:

$$\begin{aligned} B_1^b &= \sum_{y_k\in\{\pm1\},k\in[2,N]} \int_{\mathcal{P}^{N-1}} d\boldsymbol{\tau}^{\hat{1}}_{N-1}\delta_{j,1}\sum_{i=2}^{N-1}\sum_{\tilde{y}_i\in\{\pm1\}}\int_P d\tilde{\tau}_i \frac{P(\tilde{\boldsymbol{\tau}}^{i,\hat{j}}_{N-1},\tilde{\boldsymbol{y}}^{i,\hat{j}}_{N-1})}{N-1}\beta(\tau_i-\tilde{\tau}_i)\beta(\tau_j-\tilde{\tau}_i)g(\tilde{\tau}_i,\tilde{\boldsymbol{\tau}}^{i,\hat{j}}_{N-1})\delta_{\eta,1}\delta_{\tilde{y}_i,1}\delta_{y_i,1}\delta_{y_j,1} \\ &= \sum_{y_k\in\{\pm1\},k\in[2,N]} \int_{\mathcal{P}^{N-1}} d\tilde{\boldsymbol{\tau}}^{\hat{1},2}_{N-1} \sum_{\tilde{y}_1\in\{\pm1\}} P(\tilde{\boldsymbol{\tau}}^{2,\hat{1}}_{N-1},\tilde{\boldsymbol{y}}^{2,\hat{1}}_{N-1})\beta(\tau_1-\tilde{\tau}_2)g(\tilde{\tau}_2,\tilde{\boldsymbol{\tau}}^{2,\hat{1}}_{N-1})\delta_{\eta,1}\delta_{\tilde{y}_2,1}\delta_{y_2,1}\delta_{y_1,1} \end{aligned} \tag{S43}$$

where the $\sum_{i=2}^N$ pulls out a $(N-1)$ factor by fixing $i=2$. By renaming $\tilde{\tau}_2\to\tilde{\tau}_1$ one obtains:

$$B_1^b = \sum_{y_k\in\{\pm1\},k\in[2,N]} \int_{\mathcal{P}^{N-1}} d\tilde{\boldsymbol{\tau}}^{1,\hat{2}}_{N-1} \sum_{\tilde{y}_1\in\{\pm1\}} P(\tilde{\boldsymbol{\tau}}^{1,\hat{2}}_{N-1},\tilde{\boldsymbol{y}}^{1,\hat{2}}_{N-1},t)g(\tilde{\tau}_1,\tilde{\boldsymbol{\tau}}^{1,\hat{2}}_{N-1})\beta(\tau_1-\tilde{\tau}_1)\delta_{\eta,1}\delta_{\tilde{y}_1,1}\delta_{y_1,1}\delta_{y_2,1}. \tag{S44}$$

Finally, the third contribution of the sum, $\sum_{i=2}^{N}\sum_{j=2\neq i}^{N}$ reads:

$$
\begin{aligned}
B_1^c =& \sum_{y_k\in\{\pm1\},k\in[2,N]} \int_{\mathcal{P}^{N-1}} d\boldsymbol{\tau}_{N-1}^{\hat{1}} \sum_{i=2}^{N}\sum_{j=2\neq i}^{N} \sum_{\tilde{y}_i=\in\{\pm1\}} \int_{\mathcal{P}} d\tilde{\tau}_i \frac{P(\tilde{\boldsymbol{\tau}}_{N-1}^{i,\hat{j}}, \tilde{\boldsymbol{y}}_{N-1}^{i,\hat{j}})}{N-1} \beta(\tau_i-\tilde{\tau}_i)\beta(\tau_j-\tilde{\tau}_i) g(\tilde{\tau}_i, \tilde{\boldsymbol{\tau}}_{N-1}^{i,\hat{j}})\delta_{\eta,1}\delta_{\tilde{y}_i,1}\delta_{y_i,1}\delta_{y_j,1} \\
=& (N-2) \sum_{y_k\in\{\pm1\},k\in[2,N]} \int_{\mathcal{P}^{N-1}} d\boldsymbol{\tau}_{N-1}^{\hat{1}} \sum_{\tilde{y}_2\in\{\pm1\}} \int_{\mathcal{P}} d\tilde{\tau}_2 P(\tilde{\boldsymbol{\tau}}_{N-1}^{2,\hat{3}}, \tilde{\boldsymbol{y}}_{N-1}^{2,\hat{3}})\beta(\tau_2-\tilde{\tau}_2)\beta(\tau_3-\tilde{\tau}_2) g(\tilde{\tau}_2, \tilde{\boldsymbol{\tau}}_{N-1}^{2,\hat{3}})\delta_{\eta,1}\delta_{\tilde{y}_2,1}\delta_{y_2,1}\delta_{y_3,1}.
\end{aligned}
\tag{S45}
$$

where first sum, $\sum_{i=2}^{N}$, pulls a $N-1$ factor by fixing $i=2$, whereas the second sum, $\sum_{j=2\neq i}^{N}$, pulls a $N-1$ factor by fixing $j=3$. By splitting the following factor $d\boldsymbol{\tau}_{N-1}^{\hat{1}}$ as $d\boldsymbol{\tau}_{N-3}^{\hat{1},\hat{2}} d\tau_2 d\tau_3$ and performing the integrals $\int_P d\tau_2\beta(\tau_2-\tilde{\tau}_2) = \int_P d\tau_3\beta(\tau_3-\tilde{\tau}_2) = 1$ one gets to:

$$
B_1^c = (N-2) \sum_{y_k\in\{\pm1\},k\in[2,N]} \int_{\mathcal{P}^{N-2}} d\tilde{\boldsymbol{\tau}}_{N-2}^{\hat{1},2,\hat{3}} \sum_{\tilde{y}_2\in\{\pm1\}} P(\tilde{\boldsymbol{\tau}}_{N-1}^{2,\hat{3}}, \tilde{\boldsymbol{y}}_{N-1}^{2,\hat{3}}) g(\tilde{\tau}_2, \tilde{\boldsymbol{\tau}}_{N-1}^{2,\hat{3}})\delta_{\eta,1}\delta_{\tilde{y}_2,1}\delta_{y_2,1}\delta_{y_3,1}.
$$

By summing all the three contributions (*S42*, *S44*, *S46*) one obtains the marginalization of the positive birth contribution:

$$
\begin{aligned}
B_1 =& 2a_{N-1,N} \sum_{y_k=\pm1,k\in[2,N]} \int_{\mathcal{P}^{N-1}} d\tilde{\boldsymbol{\tau}}_{N-1}^{1,\hat{2}} \sum_{\tilde{y}_1\in\{\pm1\}} P(\tilde{\boldsymbol{\tau}}_{N-1}^{1,\hat{2}}, \tilde{\boldsymbol{y}}_{N-1}^{1,\hat{2}}, t) g(\tilde{\tau}_1, \tilde{\boldsymbol{\tau}}_{N-1}^{1,\hat{2}})\beta(\tau_1-\tilde{\tau}_1)\delta_{\eta,1}\delta_{\tilde{y}_1,1}\delta_{y_1,1}\delta_{y_2,1} + \\
&+ (N-2)a_{N-1,N} \sum_{y_k\in\{\pm1\},k\in[2,N]} \int_{\mathcal{P}^{N-2}} d\tilde{\boldsymbol{\tau}}_{N-2}^{\hat{1},2,\hat{3}} \sum_{\tilde{y}_2\in\{\pm1\}} P(\tilde{\boldsymbol{\tau}}_{N-1}^{2,\hat{3}}, \tilde{\boldsymbol{y}}_{N-1}^{2,\hat{3}}) g(\tilde{\tau}_2, \tilde{\boldsymbol{\tau}}_{N-1}^{2})\delta_{\eta,1}\delta_{\tilde{y}_2,1}\delta_{y_2,1}.
\end{aligned}
\tag{S46}
$$

The marginalization of second term of the birth-death process, i.e. positive contribution of death, is straightforward:

$$
\begin{aligned}
B_2 =& a_{N+1,N} \sum_{i=1}^{N+1} \sum_{y_k\in\{\pm1\},k\in[2,N]} \int_{\mathcal{P}^{N-1}} d\boldsymbol{\tau}_{N-1}^{\hat{1}} \sum_{i=1}^{N+1} \sum_{y_{N+1}\in\{\pm1\}} \int_{\mathcal{P}} d\tau_i \frac{1}{N+1} \mathcal{D}(\boldsymbol{\tau}_N^{\hat{i}}, \boldsymbol{y}_N^{\hat{i}} | \boldsymbol{\tau}_{N+1}, \boldsymbol{y}_{N+1}) P(\boldsymbol{\tau}_{N+1}, \boldsymbol{y}_{N+1}, t) = \\
=& a_{N+1,N} \sum_{y_k\in\{\pm1\},k\in[2,N]} \int_{\mathcal{P}^{N-1}} d\boldsymbol{\tau}_{N-1}^{\hat{1}} \sum_{y_i\in\{\pm1\}} \int_{\mathcal{P}} d\tau_{N+1} [\mu(\tau_{N+1}, \boldsymbol{\tau_{N+1}})\delta_{y_{N+1},1} P(\boldsymbol{\tau}_{N+1}, \boldsymbol{y}_{N+1}, t) \\
&+ g(\tau_{N+1}, \boldsymbol{\tau}_{N+1})\delta_{\eta,-1}\delta_{y_{N+1},1}] P(\boldsymbol{\tau}_{N+1}, \boldsymbol{y}_{N+1}, t)],
\end{aligned}
\tag{S47}
$$

where the index i has been fixed to $i=N+1$. The third term of the master equation quantifies the negative contribution of reproduction

$$
\begin{aligned}
B_3 =& \sum_{y_k\in\{\pm1\},k\in[2,N]} \int_{\mathcal{P}^{N-1}} d\boldsymbol{\tau}_{N-1}^{\hat{1}} \sum_{i=1}^{N}\sum_{j=1\neq i}^{N+1} \sum_{\tilde{y}_i\in\{\pm1\}} \int_{\mathcal{P}^2} d\tilde{\tau}_i d\tilde{\tau}_j \frac{1}{N} \mathcal{B}(\tilde{\boldsymbol{\tau}}_{N+1}^{i,j}, \tilde{\boldsymbol{y}}_{N+1}^{i,j} | \boldsymbol{\tau}_N, \boldsymbol{y}_N) P(\boldsymbol{\tau}_N, \boldsymbol{y}_N, t) \\
=& \sum_{y_k\in\{\pm1\},k\in[2,N]} \int_{\mathcal{P}^{N-1}} d\boldsymbol{\tau}_{N-1}^{\hat{1}} \sum_{i=1}^{N} \sum_{\tilde{y}_i\in\{\pm1\}} \int_{\mathcal{P}^2} d\tilde{\tau}_i d\tau_{N+1} \mathcal{B}(\tilde{\boldsymbol{\tau}}_{N+1}^{i,N+1}, \tilde{\boldsymbol{y}}_{N+1}^{i,N+1} | \boldsymbol{\tau}_N \boldsymbol{y}_N) P(\boldsymbol{\tau}_N, \boldsymbol{y}_N, t).
\end{aligned}
\tag{S48}
$$

where the index j has been fixed to $N+1$. By splitting the sum $\sum_{i=1}^{N} = \delta_{i,1} + \sum_{i=2}^{N}$, and integrating the variations kernels one obtains:

$$
\begin{aligned}
B_3 =& \sum_{y_k\in\{\pm1\},k\in[2,N]} \int_{\mathcal{P}^{N-1}} d\boldsymbol{\tau}_{N-1}^{\hat{1}} \sum_{\tilde{y}_1\in\{\pm1\}} P(\boldsymbol{\tau}_N, \boldsymbol{y}_N, t) g(\tau_1, \boldsymbol{\tau}_N)\delta_{\eta,1}\delta_{\tilde{y}_1,1}\delta_{y_1,1} \\
&+ (N-1) \sum_{y_k\in\{\pm1\},k\in[2,N]} \int_{\mathcal{P}^{N-1}} d\boldsymbol{\tau}_{N-1}^{\hat{1}} \sum_{\tilde{y}_2\in\{\pm1\}} P(\boldsymbol{\tau}_N, \boldsymbol{y}_N, t) g(\tau_2, \boldsymbol{\tau}_N)\delta_{\eta,+1}\delta_{\tilde{y}_2,1}\delta_{y_2,1}.
\end{aligned}
\tag{S49}
$$

where in the second term we have fixed $i=2$. Finally, the fourth term, i.e. the negative contribution of death, is very similar to the second one:

$$B_4 = \sum_{y_k\in\{\pm1\},k\in[2,N]} \int_{\mathcal{P}^{N-1}} d\boldsymbol{\tau}^{\hat{1}}_{N-1} \sum_{i=1}^{N} \sum_{y_i\in\{\pm1\}} \mathcal{D}(\boldsymbol{\tau}^{\hat{i}}_{N-1},\boldsymbol{y}^{\hat{i}}_{N-1}|\boldsymbol{\tau}_N,\boldsymbol{y}_N)P(\boldsymbol{\tau}_N,\boldsymbol{y}_N,t), \tag{S50}$$

by splitting the sum as $\sum_{i=1}^N = \delta_{i,1} + \sum_{i=2}^N$, one obtains:

$$\begin{aligned} B_4 = & \sum_{y_k\in\{\pm1\},k\in[2,N]} \int_{\mathcal{P}^{N-1}} d\boldsymbol{\tau}^{\hat{1}}_{N-1} \sum_{y_1\in\{\pm1\}} \left[\mu(\tau_1,\boldsymbol{\tau_N})\delta_{y_1,1} + g(\tau_1,\boldsymbol{\tau}_N)\delta_{\eta,-1}\delta_{y_1,1}\right] P(\boldsymbol{\tau}_N,\boldsymbol{y}_N,t) \\ & + (N-1) \sum_{y_k\in\{\pm1\},k\in[2,N]} \int_{\mathcal{P}^{N-1}} d\boldsymbol{\tau}^{\hat{1}}_{N-1} \sum_{y_2\in\{\pm1\}} \left[\mu(\tau_2,\boldsymbol{\tau_N})\delta_{y_2,1} + g(\tau_2,\boldsymbol{\tau}_N)\delta_{\eta,-1}\delta_{y_2,1}\right] P(\boldsymbol{\tau}_N,\boldsymbol{y}_N,t). \end{aligned} \tag{S51}$$

By putting all terms together (S38, S46,S47, S49, S51) and marginalizing also on the y_1 variable one finally obtains the marginalized birth-death contribution:

$$\begin{aligned} \Delta_{BD}\phi^{(1)}(\tau,t) = & \sum_{y_k\in\{\pm1\},k\in[1,N]} \int_{\mathcal{P}^{N-1}} d\tau_2 \dots d\tau_N \Delta_{BD} P(\boldsymbol{\tau}_N,\mathbf{y}_N,t) \\ = & \, 2a_{N-1,N} \int_{\mathcal{P}^{N-1}} d\tilde{\boldsymbol{\tau}}^{1,\hat{2}}_{N-1} P(\tilde{\boldsymbol{\tau}}^{1,\hat{2}}_{N-1},\tilde{y}_1=1,t) g(\tilde{\tau}_1,\tilde{\boldsymbol{\tau}}^{1,\hat{2}}_{N-1})\beta(\tau_1-\tilde{\tau}_1)\delta_{\eta,1} + \\ & + a_{N-1,N}(N-2) \int_{\mathcal{P}^{N-1}} d\tilde{\tau}_2 d\boldsymbol{\tau}^{\hat{1},\hat{3}}_{N-2} P(\tilde{\boldsymbol{\tau}}^{2}_{N-1},\tilde{y}_2=1,t) g(\tilde{\tau}_2,\tilde{\boldsymbol{\tau}}^{2}_{N-1})\delta_{\eta,1} \\ & + a_{N+1,N} \int_{\mathcal{P}^{N-1}} d\boldsymbol{\tau}^{\hat{1}}_{N-1} \int_{\mathcal{P}} d\tau_{N+1} \left[\mu(\tau_{N+1},\boldsymbol{\tau_{N+1}}) + g(\tau_{N+1},\boldsymbol{\tau}_{N+1})\delta_{\eta,-1}\right] P(\boldsymbol{\tau}_{N+1},y_{N+1}=1,t) \\ & - \int_{\mathcal{P}^{N-1}} d\boldsymbol{\tau}^{\hat{1}}_{N-1} \left[g(\tau_1,\boldsymbol{\tau}_N)\delta_{\eta,1}P(\boldsymbol{\tau}_N,\tilde{y}_1=1,t) + \left(\mu(\tau_1,\boldsymbol{\tau_N}) + g(\tau_1,\boldsymbol{\tau}_N)\delta_{\eta,-1}\right)P(\boldsymbol{\tau}_N,y_1=1,t)\right] \\ & - (N-1)\cdot \int_{\mathcal{P}^{N-1}} d\boldsymbol{\tau}^{\hat{1}}_{N-1} \left[g(\tau_2,\boldsymbol{\tau}_N)\delta_{\eta,1}P(\boldsymbol{\tau}_N,\tilde{y}_2=1,t) + (\mu(\tau_2,\boldsymbol{\tau_N}) + g(\tau_2,\boldsymbol{\tau}_N)\delta_{\eta,-1})P(\boldsymbol{\tau}_N,y_2=1,t)\right] \\ & - \Lambda_N(t)\phi^{(1)}(\tau,t). \end{aligned} \tag{S52}$$

Moran Process Contribution The marginalization of the Moran process contribution (when $N=K$)

$$\Delta_M\phi^{(1)}(\tau,y,t) = \sum_{y_k\in\{\pm1\},k\in[2,N]} \int_{\mathcal{P}^{N-1}} d\tau_2 \dots d\tau_N \Delta_M P(\boldsymbol{\tau}_N,\mathbf{y}_N,t). \tag{S53}$$

is analogous to the birth-death one. It is necessary to consider separately the positive and negative contributions,

$$\Delta_M\phi^{(1)}(\tau,y,t) = M_1 - M_2. \tag{S54}$$

The first one reads:

$$M_1 = \sum_{y_k\in\{\pm1\},k\in[2,N]} \int_{\mathcal{P}^{N-1}} d\tau_2 \dots d\tau_N \sum_{i=1}^{N} \sum_{j=1,j\neq i}^{N} \sum_{\tilde{y}_i\in\{\pm1\}} \sum_{\tilde{y}_j\in\{\pm1\}} \int d\tilde{\tau}_i d\tilde{\tau}_j W_M(\boldsymbol{\tau}_N,\boldsymbol{y}_N|\tilde{\boldsymbol{\tau}}^{ij}_N,\tilde{\boldsymbol{y}}^{ij}_N)P(\tilde{\boldsymbol{\tau}}^{ij}_N,\tilde{\boldsymbol{y}}^{ij}_N,t). \tag{S55}$$

As done before, one can split the sum as $\sum_{i=1}^N\sum_{j=1,i\neq j}^N = \delta_{i,1}\sum_{j=2}^N + \delta_{j,1}\sum_{i=2}^N + \sum_{i=2}^N\sum_{j=2,j\neq i}^N$ to respectively separate three contributions:

$$M_1 = M_1^a + M_1^b + M_1^c. \tag{S56}$$

The calculation is identical to the first term of the birth-death part (S46). The three contributions read:

$$M_1^a = M_1^b = \sum_{y_k\in\{\pm 1\},k\in[2,N]} \int_{\mathcal{P}^N} d\tilde{\boldsymbol{\tau}}_N^{1,2} \sum_{\tilde{y}_1\in\{\pm 1\}} P(\tilde{\boldsymbol{\tau}}_N^{1,2}, \tilde{\boldsymbol{y}}_N^{1,2}, t) g(\tilde{\tau}_1, \tilde{\boldsymbol{\tau}}_N^{1,2}) \beta(\tau_1 - \tilde{\tau}_1) \delta_{\eta,1}\delta_{\tilde{y}_1,1}\delta_{y_1,1}\delta_{y_2,1}, \tag{S57}$$

$$M_1^c = (N-2) \sum_{y_k\in\{\pm 1\},k\in[2,N]} \int_{\mathcal{P}^N} d\boldsymbol{\tau}_N^{\hat{1}} \sum_{\tilde{y}_2\in\{\pm 1\}} \int_{\mathcal{P}} d\tilde{\tau}_2 P(\tilde{\boldsymbol{\tau}}_N^{2,3}, \tilde{\boldsymbol{y}}_N^{2,3}) \beta(\tau_2 - \tilde{\tau}_2)\beta(\tau_3 - \tilde{\tau}_2) g(\tilde{\tau}_2, \tilde{\boldsymbol{\tau}}_{N-1}^{2,3}) \delta_{\eta,1}\delta_{\tilde{y}_2,1}\delta_{y_2,1}\delta_{y_3,1}. \tag{S58}$$

By summing them all, one obtains:

$$\begin{aligned} M_1 = &\sum_{y_k\in\{\pm 1\},k\in[2,N]} \int_{\mathcal{P}^N} d\tilde{\boldsymbol{\tau}}_N^{1,2} \sum_{\tilde{y}_1\in\{\pm 1\}} P(\tilde{\boldsymbol{\tau}}_N^{1,2}, \tilde{\boldsymbol{y}}_N^{1,2}, t) g(\tilde{\tau}_1, \tilde{\boldsymbol{\tau}}_N^{1,2}) \beta(\tau_1 - \tilde{\tau}_1) \delta_{\eta,1}\delta_{\tilde{y}_1,1}\delta_{y_1,1}\delta_{y_2,1} \\ &+ (N-2) \sum_{y_k\in\{\pm 1\},k\in[2,N]} \int_{\mathcal{P}^N} d\boldsymbol{\tau}_N^{\hat{1}} \sum_{\tilde{y}_2\in\{\pm 1\}} \int_{\mathcal{P}} d\tilde{\tau}_2 P(\tilde{\boldsymbol{\tau}}_N^{2,3}, \tilde{\boldsymbol{y}}_N^{2,3}) \beta(\tau_2 - \tilde{\tau}_2)\beta(\tau_3 - \tilde{\tau}_2) g(\tilde{\tau}_2, \tilde{\boldsymbol{\tau}}_{N-1}^{2,3}) \delta_{\eta,1}\delta_{\tilde{y}_2,1}\delta_{y_2,1}\delta_{y_3,1}. \end{aligned} \tag{S59}$$

The marginalization of the negative term is analogous to the birth-death one ($S49$), leading to:

$$\begin{aligned} M_2 = &\sum_{y_k\in\{\pm 1\},k\in[2,N]} \int_{\mathcal{P}^{N-1}} d\boldsymbol{\tau}_N^{\hat{1}} \sum_{\tilde{y}_1\in\{\pm 1\}} P(\boldsymbol{\tau}_N, \boldsymbol{y}_N, t) g(\tau_1, \boldsymbol{\tau}_N) \delta_{\eta,1}\delta_{\tilde{y}_1,1}\delta_{y_1,1} \\ &+ (N-1) \sum_{y_k\in\{\pm 1\},k\in[2,N]} \int_{\mathcal{P}^{N-1}} d\boldsymbol{\tau}_{N-1}^{\hat{1}} \sum_{\tilde{y}_2\in\{\pm 1\}1} P(\boldsymbol{\tau}_N, \boldsymbol{y}_N, t) g(\tau_2, \boldsymbol{\tau}_N) \delta_{\eta,1}\delta_{\tilde{y}_2,1}\delta_{y_2,1}. \end{aligned} \tag{S60}$$

By summing the two terms and summing over $y_1$one obtains the marginalized contribution of the Moran process:

$$\begin{aligned} \Delta_M \phi^{(1)}(\tau, t) = &\sum_{y_k\in\{=\pm 1\},k\in[2,N]} \int_{\mathcal{P}^N} d\tilde{\boldsymbol{\tau}}_N^{1,2} \sum_{\tilde{y}_1\in\{\pm 1\}} P(\tilde{\boldsymbol{\tau}}_N^{1,2}, \tilde{\boldsymbol{y}}_N^{1,2}, t) g(\tilde{\tau}_1, \tilde{\boldsymbol{\tau}}_N^{1,2}) \beta(\tau_1 - \tilde{\tau}_1) \delta_{\eta,1}\delta_{\tilde{y}_1,1}\delta_{y_1,1}\delta_{y_2,1} \\ &+ (N-2) \sum_{y_k in\{\pm 1\},k\in[2,N]} \int_{\mathcal{P}^N} d\boldsymbol{\tau}_N^{\hat{1}} \sum_{\tilde{y}_2 in\{\pm 1\}} \int_{\mathcal{P}} d\tilde{\tau}_2 P(\tilde{\boldsymbol{\tau}}_N^{2,3}, \tilde{\boldsymbol{y}}_N^{2,3}) \beta(\tau_2 - \tilde{\tau}_2)\beta(\tau_3 - \tilde{\tau}_2) g(\tilde{\tau}_2, \tilde{\boldsymbol{\tau}}_{N-1}^{2,3}) \delta_{\eta,1}\delta_{\tilde{y}_2,1}\delta_{y_2,1}\delta_{y_3,1} \\ &- \sum_{y_k in\{\pm 1\},k\in[2,N]} \int_{\mathcal{P}^{N-1}} d\boldsymbol{\tau}_N^{\hat{1}} \sum_{\tilde{y}_1=\pm 1} P(\boldsymbol{\tau}_N, \boldsymbol{y}_N, t) g(\tau_1, \boldsymbol{\tau}_N) \delta_{\eta,1}\delta_{\tilde{y}_1,1}\delta_{y_1,1} \\ &- (N-1) \sum_{y_k in\{\pm 1\},k\in[2,N]} \int_{\mathcal{P}^{N-1}} d\boldsymbol{\tau}_{N-1}^{\hat{1}} \sum_{\tilde{y}_2 in\{\pm 1\}} P(\boldsymbol{\tau}_N, \boldsymbol{y}_N, t) g(\tau_2, \boldsymbol{\tau}_N) \delta_{\eta,2}\delta_{\tilde{y}_2,1}\delta_{y_2,1}\delta_{y_3,1}. \end{aligned} \tag{S61}$$

Growing and Dormant state contribution Using the definition of this part of the process from the master eq.(S30, S31):

$$\begin{aligned} \Delta_{GD}\phi^{(1)}(\tau, y, t) = &\sum_{y_j in\{\pm 1\},j\in[2,N]} \int_{\mathcal{P}^{N-1}} d\tau_2 \dots d\tau_N \Delta_{GD} P(\boldsymbol{\tau}_N, \mathbf{y}_N, t) = \\ = &\sum_{y_j in\{\pm 1\},j\in[2,N]} \int_{\mathcal{P}^{N-1}} d\boldsymbol{\tau}_N^{\hat{1}} \sum_{i=1}^{N} \sum_{\tilde{y}_i in\{\pm 1\}} \Big[W_{GD}(\mathbf{y}_N|\tilde{\mathbf{y}}_N^i, \boldsymbol{\tau}_N) P(\boldsymbol{\tau}_N, \tilde{\mathbf{y}}_N^i, t) - W_{GD}(\tilde{\mathbf{y}}_N^i|\mathbf{y}_N, \boldsymbol{\tau}_N) P(\boldsymbol{\tau}_N, \mathbf{y}_N, t) \Big] = \\ = &\sum_{y_j in\{\pm 1\},j\in[2,N]} \int_{\mathcal{P}^{N-1}} d\boldsymbol{\tau}_{N-1}^{\hat{1}} \sum_{i=1}^{N} \sum_{\tilde{y}_i in\{\pm 1\}} \Big[\big\{ \delta_{\tilde{y}_i,-1}\delta_{y_i,1} A(\tau_i, \boldsymbol{\tau}_N) + \delta_{\tilde{y}_i,1}\delta_{y_i,-1}\delta_{\eta,-1} S(\tau_i, \boldsymbol{\tau}_N) \big\} P(\boldsymbol{\tau}_N, \tilde{\mathbf{y}}_N^i, t) \\ &- \big\{ \delta_{\tilde{y}_i,1}\delta_{y_i,-1} A(\tau_i, \boldsymbol{\tau}_N) + \delta_{\tilde{y}_i,-1}\delta_{y_i,1}\delta_{\eta,-1} S(\tau_i, \boldsymbol{\tau}_N) \big\} P(\boldsymbol{\tau}_N, \mathbf{y}_N, t) \Big]., \end{aligned} \tag{S62}$$

and splitting the sum in i as $\sum_{i=1}^{N}=\sum_{i=1}^{N}\delta_{i,1}+\sum_{i=2}^{N}$, the expression is composed by two terms:

$$\Delta_{GD}\phi^{(1)}(\tau,y,t)=G_1+G_2, \tag{S63}$$

as defined now. Let us consider first the second term, which takes into account the transitions in which individual 1 is not involved:

$$G_2=\sum_{y_j in\{\pm1\},j\in[2,N]}\int_{\mathcal{P}^{N-1}}d\boldsymbol{\tau}^{\hat{1}}_{N-1}\sum_{i=2}^{N}\sum_{\tilde{y}_i in\{\pm1\}}\left\{\delta_{\tilde{y}_i,-1}\delta_{y_i,1}A(\tau_i,\boldsymbol{\tau}_N)+\delta_{\tilde{y}_i,1}\delta_{y_i,-1}\delta_{\eta,-1}S(\tau_i,\boldsymbol{\tau}_N)\right\}P(\boldsymbol{\tau}_N,\tilde{\mathbf{y}}^i_N,t)$$
$$-\left\{\delta_{\tilde{y}_i,1}\delta_{y_i,-1}A(\tau_i,\boldsymbol{\tau}_N)+\delta_{\tilde{y}_i,-1}\delta_{y_i,1}\delta_{\eta,-1}S(\tau_i,\boldsymbol{\tau}_N)\right\}P(\boldsymbol{\tau}_N,\mathbf{y}_N,t)\Big]. \tag{S64}$$

Using individual indistinguishability, the index i is fixed to $i=2$ and removing the sum $\sum_{i=2}^{N}$ by pulling out a factor $(N-1)$:

$$G_2=(N-1)\sum_{y_j in\{\pm1\},j\in[2,N]}\int_{\mathcal{P}^{N-1}}d\boldsymbol{\tau}^{\hat{1}}_{N-1}\sum_{\tilde{y}_2 in\{\pm1\}}\Big[\left\{\delta_{\tilde{y}_i,-1}\delta_{y_2,1}A(\tau_2,\boldsymbol{\tau}_N)+\delta_{\tilde{y}_2,1}\delta_{y_2,-1}\delta_{\eta,-1}S(\tau_2,\boldsymbol{\tau}_N)\right\}P(\boldsymbol{\tau}_N,\tilde{\mathbf{y}}^2_N,t)$$
$$-\left\{\delta_{\tilde{y}_2,1}\delta_{y_2,-1}A(\tau_2,\boldsymbol{\tau}_N)+\delta_{\tilde{y}_2,-1}\delta_{y_2,1}\delta_{\eta,-1}S(\tau_2,\boldsymbol{\tau}_N)\right\}P(\boldsymbol{\tau}_N,\mathbf{y}_N,t)\Big], \tag{S65}$$

and by marginalizing this expression in y_j, $j=3,\ldots,N$ one obtains:

$$G_2=(N-1)\sum_{y_2 in\{\pm1\}}\int_{\mathcal{P}^{N-1}}d\boldsymbol{\tau}^{\hat{1}}_{N-1}\sum_{\tilde{y}_2 in\{\pm1\}}\Big[\left\{\delta_{\tilde{y}_2,-1}\delta_{y_2,1}A(\tau_2,\boldsymbol{\tau}_N)+\delta_{\tilde{y}_2,1}\delta_{y_2,-1}\delta_{\eta,-1}S(\tau_2,\boldsymbol{\tau}_N)\right\}P(\boldsymbol{\tau}_N,y_1,\tilde{y}_2,t)$$
$$-\left\{\delta_{\tilde{y}_2,1}\delta_{y_2,-1}A(\tau_2,\boldsymbol{\tau}_N)+\delta_{\tilde{y}_2,-1}\delta_{y_2,1}\delta_{\eta,-1}S(\tau_2,\boldsymbol{\tau}_N)\right\}P(\boldsymbol{\tau}_N,y_1,y_2,t)\Big]. \tag{S66}$$

Obviously, if the individual before the transition was in a state, $\tilde{y}_2 in\,\{\pm1\}$, after it the state is opposite one $y_2 in\,\{\pm1\}$, implying that the sum of the contribution is zero:

$$G_2=(N-1)\int_{\mathcal{P}^{N-1}}d\boldsymbol{\tau}^{\hat{1}}_{N-1}\Big[\left\{A(\tau_2,\boldsymbol{\tau}_N)P(\boldsymbol{\tau}_N,y_1,\tilde{y}_2=-1,t)-S(\tau_2,\boldsymbol{\tau}_N)P(\boldsymbol{\tau}_N,y_1,y_2=+1,t)\right\}_{y_2=+1,\tilde{y}_2=-1}$$
$$+\left\{S(\tau_2,\boldsymbol{\tau}_N)P(\boldsymbol{\tau}_N,y_1,\tilde{y}_2=+1,t)-A(\tau_2,\boldsymbol{\tau}_N)P(\boldsymbol{\tau}_N,y_1,y_2=-1,t)\right\}_{y_2=-1,\tilde{y}_2=+1}\Big]=0. \tag{S67}$$

Thus, the first term $\sum_{i=1}^{N}\delta_{i,1}$, which takes in count the transitions in which individual 1 is involved, is the only net contribution to the growing-dormant part:

$$\Delta_{GD}\phi^{(1)}(\tau,y,t)=G_1=\sum_{y_j in\{\pm1\},j\in[2,N]}\int_{\mathcal{P}^{N-1}}d\boldsymbol{\tau}^{\hat{1}}_{N-1}\sum_{\tilde{y}_i in\{\pm1\}}\Big[\left\{\delta_{\tilde{y}_1,-1}\delta_{y_1,1}A(\tau_1,\boldsymbol{\tau}_N)+\right. \tag{S68}$$
$$\left.+\delta_{\tilde{y}_1,1}\delta_{y_1,-1}\delta_{\eta,-1}S(\tau_1,\boldsymbol{\tau}_N)\right\}P(\boldsymbol{\tau}_N,\tilde{\mathbf{y}}^1_N,t)-\left\{\delta_{\tilde{y}_1,1}\delta_{y_1,-1}A(\tau_1,\boldsymbol{\tau}_N)+\delta_{\tilde{y}_1,-1}\delta_{y_1,1}\delta_{\eta,-1}S(\tau_1,\boldsymbol{\tau}_N)\right\}P(\boldsymbol{\tau}_N,\mathbf{y}_N,t)\Big].$$

This is because index 1 is the only one that remains when function $P(\boldsymbol{\tau}_N,\boldsymbol{y}_N,t)$ is marginalized, i.e., $\tau=\tau_1$. It is possible to separate the previous equation in two contributions:

$$\Delta_{GD}\phi^{(1)}(\tau,y,t)=\Delta_{GD}\phi^{(1)}(\tau,y=+1,t)+\Delta_{GD}\phi^{(1)}(\tau,y=+1,t), \tag{S69}$$

where

$$\Delta_{GD}\phi(\tau,y=+1,t)=\sum_{y_j in\{\pm1\},j\in[2,N]}\int_{\mathcal{P}^{N-1}}d\boldsymbol{\tau}^{\hat{1}}_{N-1}\Big[A(\tau_1,\boldsymbol{\tau}_N)P(\boldsymbol{\tau}_N,y_1=+1,t)-S(\tau_1,\boldsymbol{\tau}_N)P(\boldsymbol{\tau}_N,y_1=-1,t)\Big], \tag{S70}$$

and

$$\Delta_{GD}\phi(\tau,y=-1,t)=\sum_{y_j in\{\pm1\},j\in[2,N]}\int_{\mathcal{P}^{N-1}}d\boldsymbol{\tau}^{\hat{1}}_{N-1}\Big[S(\tau_1,\boldsymbol{\tau}_N)P(\boldsymbol{\tau}_N,\tilde{y}_1=+1,t)-A(\tau_1,\boldsymbol{\tau}_N)P(\boldsymbol{\tau}_N,y_1=-1,t)\Big]. \tag{S71}$$

Given that the rates do not depend on the variable y, one can marginalize in the variables using the sum $\sum_{y_j in\{\pm 1\}, j\in[2,N]}$ and obtain:

$$\Delta_{GD}\phi^{(1)}(\tau,t) = \Delta_{GD}\phi_G^{(1)}(\tau,t) + \Delta_{GD}\phi_D^{(1)}(\tau,t), \tag{S72}$$

where the growing and dormant densities have been defined as:

$$\phi_G^{(1)}(\tau,t) \equiv \phi^{(1)}(\tau, y=+1, t), \tag{S73}$$
$$\phi_D^{(1)}(\tau,t) \equiv \phi^{(1)}(\tau, y=-1, t); \tag{S74}$$

while their evolution is given by:

$$\int_{\mathcal{P}^{N-1}} d\boldsymbol{\tau}_{N-1}^{\hat{1}} \Big[A(\tau_1,\boldsymbol{\tau}_N)P(\boldsymbol{\tau}_N,\tilde{y}_1=-1,t) - S(\tau_1,\boldsymbol{\tau}_N)P(\boldsymbol{\tau}_N,y_1=+1,t)\Big] \equiv \Delta_{GD}\phi_G(\tau,t), \tag{S75}$$

$$\int_{\mathcal{P}^{N-1}} d\boldsymbol{\tau}_{N-1}^{\hat{1}} \Big[S(\tau_1,\boldsymbol{\tau}_N)P(\boldsymbol{\tau}_N,y_1=+1,t) - A(\tau_1,\boldsymbol{\tau}_N)P(\boldsymbol{\tau}_N,\tilde{y}_1=-1,t)\Big] \equiv \Delta_{GD}\phi_D(\tau,t). \tag{S76}$$

B. Mean Field Approximation

To proceed further, one can first apply the individual rate approximation, i.e the rates depend just on the single individual traits:

$$g(\tau_i,\boldsymbol{\tau}_N) = g(\tau_i), \qquad \mu(\tau_i,\boldsymbol{\tau}_N) = \mu(\tau_i), \qquad A(\tau_i,\boldsymbol{\tau}_N) = A(\tau_i), \qquad S(\tau_i,\boldsymbol{\tau}_N) = S(\tau_i). \tag{S77}$$

Thanks to this approximation it is possible to integrate over most of individuals traits, but it leaves the dependence on the two-cells density as a kind of "correlation kernel" in the birth-death contribution eq.(S52):

$$\begin{aligned}\Delta_{BD}\phi^{(1)}(\tau_1,t) = {} & 2a_{N-1,N}\delta_{\eta,1}\int_{\mathcal{P}}\phi_G^{(1)}(\tilde{\tau}_1,t)g(\tilde{\tau}_1)\beta(\tau_1-\tilde{\tau}_1)d\tilde{\tau}_1 + a_{N-1,N}(N-2)\int_{\mathcal{P}}d\tau_2\phi^{(2)}(\tau_1,\tau_2,y_2=1,t)g(\tau_2)\delta_{\eta,1} \\ & + a_{N+1,N}[\bar{\mu}_{N+1}+\delta_{\eta,-1}\bar{g}_{N+1}]\phi^{(1)}(\tau_1,t) - [\mu(\tau_1)+g(\tau_1)]\,\phi_G^{(1)}(\tau_1,t) \\ & - (N-1)\int_{\mathcal{P}}d\tau_2\,[g(\tau_2)+\mu(\tau_2)]\,\phi^{(2)}(\tau_1,\tau_2,y_2=1,t) - \Lambda_N(t)\phi^{(1)}(\tau,t);\end{aligned} \tag{S78}$$

where we have used the definition of growing/dormant densities (S73), the $2-cells$ density function (S35) and the fact that $1=\delta_{\eta,1}+\delta_{\eta,-1}$.
Similarly for the Moran process contribution (S61) reads:

$$\begin{aligned}\Delta_M\phi^{(1)}(\tau_1,t) = {} & 2\int_{\mathcal{P}}d\tilde{\tau}_1\phi^{(1)}(\tilde{\tau}_1)g(\tilde{\tau}_1)\beta(\tau_1-\tilde{\tau}_1) - g(\tau_1)\phi_G^{(1)}(\tau_1,t) \\ & + \int_{\mathcal{P}^2}d\tilde{\tau}_2 d\tau_2\phi^{(2)}(\tau_1,\tilde{\tau}_2,\tilde{y}_2=1,t)g(\tilde{\tau}_2)\left[(N-1)\beta(\tau_2-\tilde{\tau}_2)-(N-1)\delta(\tau_2-\tilde{\tau}_2)\right].\end{aligned} \tag{S79}$$

On the contrary, in the marginalized growing-dormant part, eq.(S75-S76) , the rate approximation (S77) is enough to reach the final expression:

$$\Delta_{GD}\phi_G^{(1)}(\tau,t) = A(\tau)\phi_D^{(1)}(\tau,t) - \delta_{\eta,-1}S(\tau)\phi_G^{(1)}(\tau,t), \tag{S80}$$

$$\Delta_{GD}\phi_D^{(1)}(\tau,t) = \delta_{\eta,-1}S(\tau)\phi_G^{(1)}(\tau,t) - A(\tau)\phi^{(1)}(\tau,t). \tag{S81}$$

To simplify further the birth-death (S78) and Moran (S79) part one proceeds with a second mean field approximation. Specifically, we assume that the correlation between individuals is negligible. This is expected to be true in the

thermodynamic limit $N \to \infty$ [2, 6]. In such a case one can factorize the probability distribution:

$$P(\tau_1, \dots, \tau_N) = \phi^{(N)}(\tau_1, \dots, \tau_N) = \prod_{i=1}^{N} \phi(\tau_i), \tag{S82}$$

and the 1-cell density can be replaced by its mean-field approximation (removing also the individual-subindexτ_1):

$$\phi^{(1)}(\tau, t) \equiv \phi(\tau, t), \tag{S83}$$

$$\phi^{(2)}(\tau_1, \tau_2, t) \equiv \phi(\tau_1, t)\phi(\tau_2, t). \tag{S84}$$

An important consequence of this simplification is that the normalization coefficients $a_{N,M}$ fulfills the relationship:

$$a_{N,M} = \frac{\int_{P^N} d\boldsymbol{\tau}_N \psi(\boldsymbol{\tau}_N, t)}{\int_{P^M} d\boldsymbol{\tau}_M \psi(\boldsymbol{\tau}_M, t)} = \frac{\prod_{i=1}^{N} \int_P d\tau_i \psi(\tau_i, t)}{\prod_{j=1}^{M} \int_{P^M} d\tau_j \psi(\tau_j, t)} = a^{N-M}, \tag{S85}$$

and, moreover, the different marginal averages no longer dependent on the system size:

$$\bar{g}_N = \int_{P^N} d\boldsymbol{\tau}_N g(\tau_k, \boldsymbol{\tau}_N) P(\boldsymbol{\tau}_N, y_i, t)\delta_{y_k,1} = \prod_{i=1}^{N} \int_P d\tau_i g(\tau_k) P(\boldsymbol{\tau}_N, y_i, t)\delta_{y_k,1} = \int_P d\tau_k g(\tau_k)\phi(\tau_k, t)\delta_{y_k,1} \equiv \bar{g}. \tag{S86}$$

Hence, the birth-death term (S78) becomes:

$$\begin{aligned}
\Delta_{BD}\phi(\tau, t) = &\frac{2\delta_{\eta,1}}{a} \int_{\mathcal{P}} d\tilde{\tau}_1 \phi(\tilde{\tau}, t) g(\tilde{\tau}_1)\beta(\tau_1 - \tilde{\tau}_1) + \frac{(N-2)}{a}\phi(\tau_1, t)\bar{g}\delta_{\eta,1} + \\
&+ a\left[\bar{\mu} + \bar{g}\delta_{\eta,-1}\right]\phi(\tau, t) - (g(\tau) + \mu(\tau))\phi_G(\tau, t) - (N-1)(\bar{g} + \bar{\mu})\phi(\tau, t) \\
&- \left\{ N\Big(\frac{1}{a} - 1\Big)\bar{g}\delta_{\eta,1} + \bar{\mu}(a - N) - (N - a)\bar{g}\delta_{\eta,-1} \right\} \cdot \phi(\tau, t) = \\
&= \frac{2\delta_{\eta,1}}{a}\left(\int_{\mathcal{P}} d\tilde{\tau}_1 \phi(\tilde{\tau}, t) g(\tilde{\tau}_1)\beta(\tau_1 - \tilde{\tau}_1) - \bar{g}\phi(\tau, t) \right) \\
&- (\delta_{\eta,1} + \delta_{\eta,-1})\left(g(\tau)\phi_G(\tau, t) - \bar{g}\phi(\tau, t)\right) - (\mu(\tau))\phi_G(\tau, t) - \bar{\mu}\phi(\tau, t)) .
\end{aligned} \tag{S87}$$

In practical terms $\phi(\tau, t)$ is a frequency, and, for this reason, one can evaluate the normalization constant a as $\frac{1}{a} = \frac{N-1}{N}$. Furthermore, by implementing the mean-field approximation it has been assumed the thermodynamic limit $N \to \infty$, i.e. infinite population size, hence the the normalization constant can be approximated as: $\frac{N-1}{N} \approx 1$. Thanks to all of these, equation (S87) simplifies to:

$$\begin{aligned}
\Delta_{BD}\phi(\tau, t) = &\delta_{\eta,1}\left(2\int_{\mathcal{P}} d\tilde{\tau}_1 \phi(\tilde{\tau}, t) g(\tilde{\tau}_1)\beta(\tau_1 - \tilde{\tau}_1) - g(\tau)\phi_G(\tau, t) - \bar{g}\phi(\tau, t) \right) \\
&- \delta_{\eta,-1}(g(\tau)\phi_G(\tau, t) - \bar{g}\phi(\tau, t)) - (\mu(\tau)\phi_G(\tau, t) - \bar{\mu}\phi(\tau, t)).
\end{aligned} \tag{S88}$$

Importantly, if one applies the mean-field approximation (S82) to the Moran process contribution (S79) it coincides with the contribution of the birth-death process in the fresh medium (see [5]):

$$\Delta_M\phi(\tau, t) = \delta_{\eta,1}\left(2\int_{\mathcal{P}} d\tilde{\tau}_1 \phi(\tilde{\tau}, t) g(\tilde{\tau}_1)\beta(\tau_1 - \tilde{\tau}_1) - g(\tau)\phi_G(\tau, t) - \bar{g}\phi(\tau, t) \right). \tag{S89}$$

Thus, in the mean-field description, i.e. in the thermodynamics limit, it is not necessary to take into account if the population size is constant or varying: $\Delta_D\phi(\tau, t) = \Delta_{BD}\phi(\tau, t)$.
By using the decomposition in growing and dormant densities (S73) in the birth-death part, it is possible to separate such contribution in two equations:

$$\begin{aligned}
\Delta_{BD}\phi_G(\tau, t) = &\delta_{\eta,1}\left(2\int_{\mathcal{P}} d\tilde{\tau}_1 \phi(\tilde{\tau}, t) g(\tilde{\tau}_1)\beta(\tau_1 - \tilde{\tau}_1) - (g(\tau) + \bar{g})\phi_G(\tau, t) \right) \\
&- \delta_{\eta,-1}(g(\tau) - \bar{g})\phi_G(\tau, t) - (\mu(\tau) - \bar{\mu})\phi_G(\tau, t),
\end{aligned} \tag{S90}$$

$$\Delta_{BD}\phi_D(\tau, t) = -(\bar{g}[\delta_{\eta,1} - \delta_{\eta,-1}] - \bar{\mu})\phi_D(\tau, t). \tag{S91}$$

By summing the birth-death (S90)and the growing-dormant (S80) contributions, and by rewriting the deltas as

$$\delta_{\eta,1} = \frac{\eta+1}{2}, \qquad \delta_{\eta,-1} = \frac{1-\eta}{2} \tag{S92}$$

one finally obtains the general mean-field equations :

$$\begin{aligned}\dot{\phi}_G(\tau,t) = &\frac{1+\eta}{2}\Big[-g(\tau)\phi_G(\tau,t) + 2\int_0^\infty d\tilde{\tau} g(\tilde{\tau})\beta(\tau-\tilde{\tau})\phi_G(\tilde{\tau},t) - \phi_G(\tau,t)\int_0^\infty d\tilde{\tau}\phi_G g(\tilde{\tau})(\tilde{\tau},t)\Big]\\ &- \frac{1-\eta}{2}\Big[\Big(g(\tau) - \int_0^\infty d\tilde{\tau} g(\tilde{\tau})\phi_G(\tilde{\tau},t)\Big) + S(\tau)\Big]\phi_G(\tau,t)\\ &+ A(\tau)\phi_D(\tau,t) - \mu(\tau)\phi_G(\tau,t) + \bar{\mu}\phi(\tau,t)\end{aligned} \tag{S93}$$

$$\dot{\phi}_D(\tau,t) = -\Big(\eta\int_0^\infty d\tilde{\tau} g(\tilde{\tau})\phi_G(\tilde{\tau},t) + A(\tau)\Big)\phi_D(\tau,t) \;+ S(\tau)\frac{1-\eta}{2}\phi_G(\tau,t) + \bar{\mu}\phi_D(\tau,t). \tag{S94}$$

By adding these two eqs. one can easily compute the total marginalized distribution :

$$\begin{aligned}\dot{\phi}(\tau,t) &= \dot{\phi}_G(\tau,t) + \dot{\phi}_D(\tau,t)\\ &= \frac{1+\eta}{2}\Big(2\int_0^\infty d\tilde{\tau} g(\tilde{\tau})\beta(\tau-\tilde{\tau})\phi_G(\tilde{\tau},t) - g(\tau)\phi_G(\tau,t) - \Big[\int_0^\infty d\tilde{\tau} g(\tilde{\tau})\phi_G(\tilde{\tau},t)\Big]\phi(\tau,t)\Big)\\ &- \frac{1-\eta}{2}\Big(g(\tau)\phi_G(\tau,t) - \bar{g}\phi(\tau,t)\Big) - \mu(\tau)\phi_G(\tau,t) + \bar{\mu}\phi(\tau,t).\end{aligned} \tag{S95}$$

Finally, fixing the rates as

$$g(\tau_i) = b = \text{const}, \qquad \mu(\tau_i) = 0, \qquad A(\tau_i) = 1/\tau_i \qquad , S(\tau_i) = s = \text{const}; \tag{S96}$$

and by inserting them in the mean-field equation (S93) one finds the equations (1) and (2) of the main text.

S3. SMALL VARIATION APPROXIMATION

When variations are small, it is possible to perform a diffusive approximation to equation (S95). Specifically, we consider the term proportional to the variation kernel, $\beta(\tau-\tilde{\tau};\tilde{\tau})$, which regulates the probability of changing from trait $\tilde{\tau}$ to τ during reproduction, and expand it around $\tilde{\tau}\sim\tau$. Note that β depends on the magnitude of variation, $\delta = \tau - \tilde{\tau}$, but can also depend explicitly on the value of the starting trait, $\tilde{\tau}$, (i.e. state dependent, or multiplicative variation). Given that β is a probability distribution of variation, hence: $\int_{-\tilde{\tau}}^\infty d\delta\beta(\delta;\tilde{\tau}) = 1$. Furthermore, one can assume it to be symmetric in δ: $\beta(\delta;\tilde{\tau}) = \beta(-\delta;\tilde{\tau})$; and with finite first and second moments:

$$\mu(\tilde{\tau}) = \int_{-\tilde{\tau}}^\infty d\delta\delta\beta(\delta;\tilde{\tau}), \qquad \sigma(\tilde{\tau}) = \int_{-\tilde{\tau}}^\infty d\delta\delta^2\beta(\delta;\tilde{\tau}). \tag{S97}$$

Let us separate the mean-field equation for the birth-death change of the growing individual as(S90) in two parts:

$$\Delta_{BD}\phi_G(\tau,t) = \Delta_1\phi_G(\tau,t) + \Delta_2\phi_G(\tau,t), \tag{S98}$$

where the first part involves the local variation events:

$$\Delta_1\phi_G(\tau,t) = (1+\eta)\left(\int_0^\infty d\tilde{\tau}\phi_G(\tilde{\tau},t)g(\tilde{\tau})\beta(\tau-\tilde{\tau};\tilde{\tau}) - g(\tau)\phi_G(\tau,t)\right), \tag{S99}$$

and the second involves the remaining rates:

$$\begin{aligned}\Delta_2\phi_G(\tau,t) &= \eta(g(\tau)-\bar{g})\phi_G(\tau,t) - (\mu(\tau)-\bar{\mu})\phi_G =\\ &= [(g(\tau)-\mu(\tau)) - (\bar{g}-\bar{\mu})]\,\phi_G(\tau,t),\end{aligned} \tag{S100}$$

where the deltas have been replaced as in eq.(S92). Considering now Δ_1(S99), which does conserve the probability and has the typical structure of a master equation [8]):

$$\Delta_1\phi_G(\tau,t) = (\eta+1)\int_0^\infty d\tilde{\tau}\Big[W(\tilde{\tau},\tau)\phi_G(\tilde{\tau},t) - W(\tau,\tilde{\tau})\phi(\tau,t)\Big], \tag{S101}$$

with the "effective" stochastic transition rate from phenotype $\tilde{\tau}$ to τ:

$$W(\tilde{\tau},\tau) = g(\tilde{\tau})\beta(\tau-\tilde{\tau};\tilde{\tau}). \tag{S102}$$

Thus it is possible to perform a Kramers-Moyal expansion of eq.(S101) around $\tau=\tilde{\tau}$. The final trait can be written as the initial one plus the "jump" amplitude δ: $\tau=\tilde{\tau}+\delta$, allowing one to rewrite the transition rate as function just of the initial trait and of the jump amplitude:

$$W(\tilde{\tau},\tau) = W(\tau-\delta,\tau) := W(\tau-\delta;\delta) = g(\tau-\delta)\beta(\delta;\tau-\delta). \tag{S103}$$

Hence, equation(S99) can be rewritten as:

$$\Delta_1\phi_G(\tau,t) = (\eta(t)+1)\int_{-\infty}^{\tau} d\delta[W(\tau-\delta;\delta)\phi(\tau-\delta,t) - W(\tau;-\delta)\phi(\tau,t)], \tag{S104}$$

where we have performed the following change of variable:

$$\int_0^\infty d\tilde{\tau}W(\tilde{\tau},\tau)\phi_G(\tilde{\tau},t) = -\int_\tau^{\tau-\infty} d\delta W(\tau-\delta;\delta)\phi(\tau-\delta,t) = \int_{-\infty}^{\tau} d\delta W(\tau-\delta;\delta)\phi(\tau-\delta,t). \tag{S105}$$

One can consider δ as a small change in the *first* variable of the rate, and Taylor expand uo to second order:

$$W(\tau-\delta;\delta)\phi(\tau-\delta,t) = W(\tau;\delta)\phi(\tau,t) - \delta\partial_\tau W(\tau;\delta)\phi(\tau,t) + \frac{\delta^2}{2}\partial_\tau^2 W(\tau;\delta)\phi(\tau,t) + O(\delta^3). \tag{S106}$$

Inserting it back in the equation and noting that $W(\tau;\delta)=W(\tau;-\delta)$, one obtains:

$$\Delta_1\phi_G(\tau,t) = (\eta+1)\left(-\partial_\tau m_1(\tau)\phi_G(\tau,t) + \partial_\tau^2 m_2(\tau)\phi_G(\tau,t)\right), \tag{S107}$$

where

$$m_1(\tau) = 2\int_{-\infty}^{\tau} d\delta\delta\beta(\delta;\tau)g(\tau)\phi_G(\tau,t) = \theta(\tau)g(\tau)\phi_G(\tau,t), \tag{S108}$$

$$m_2(\tau) = \int_{-\infty}^{\tau} d\delta\delta^2\beta(\delta;\tau)g(\tau)\phi_G(\tau,t) = \frac{\sigma^2(\tau)}{2}g(\tau)\phi_G(\tau,t). \tag{S109}$$

Plugging the eq. for Δ_1 (S107) into eq.(S98), and then inserting it back in the mean-field equation for the growing population (S95), one obtains:

$$\begin{aligned}\partial_t\phi_G(\tau,t) = \left(\Big\{g(\tau)\eta-\mu(\tau)\Big\} - \Big\{\bar{g}\eta-\bar{\mu}\Big\}\right)\phi_G(\tau,t) - (1+\eta)\partial_\tau\theta(\tau)g(\tau)\phi_G(\tau,t) + \frac{1+\eta}{2}\partial_\tau^2\sigma^2(\tau)g(\tau)\phi_G(\tau,t)\\ + A(\tau)\phi_D(\tau,t) - \frac{1-\eta}{2}S(\tau)\phi_G(\tau,t).\end{aligned} \tag{S110}$$

By adding $\partial_t\phi_G$ (S110) and $\partial_t\phi_D$ (S94) one obtains the following expression for $\partial_t\phi(\tau,t)$ within the small-variation approximation:

$$\begin{aligned}\partial_t\phi(\tau,t) = \Big\{g(\tau)\eta-\mu(\tau)\Big\}\phi_G(\tau,t) - \Big\{\bar{g}\eta-\bar{\mu}\Big\}\phi(\tau,t) +\\ -(1+\eta)\partial_\tau\theta(\tau)g(\tau)\phi_G(\tau,t) + \frac{1+\eta}{2}\partial_\tau^2\sigma^2(\tau)g(\tau)\phi_G(\tau,t).\end{aligned} \tag{S111}$$

By choosing the rates as in main text(S96), one obtains the equations for the growing and dormant densities:

$$\dot{\phi}_G(\tau,t) = b\left(1 - \int_0^\infty d\tilde{\tau}\phi_G(\tilde{\tau},t)\right)\phi_G(\tau,t) - s\frac{(1-\eta)}{2}\phi_G(\tau,t) + \frac{1}{\tau}\phi_D(\tau,t) + \\ - b(1+\eta(t))\partial_\tau\theta(\tau)\phi_G(\tau,t) + b\left(\frac{1+\eta}{2}\right)\partial_\tau^2\sigma^2(\tau)\phi_G(\tau,t), \tag{S112}$$

$$\dot{\phi}_D(\tau,t) = -\frac{1}{\tau}\phi_D(\tau,t) + s\frac{(1-\eta)}{2}\phi_G(\tau,t) - b\phi_D(\tau,t)\cdot\int_0^\infty d\tilde{\tau}\phi_G(\tilde{\tau},t). \tag{S113}$$

In addition, one can define an effective fitness function $f(\tau,t) \equiv b\frac{\phi_G(\tau,t)}{\phi(\tau,t)}$, leading to the following expression of eq.(S111) :

$$\partial_t\phi(\tau,t) = \eta(t)\left(f(\tau,t) - \bar{f}\right)\phi(\tau,t) - (1+\eta(t))\partial_\tau\theta(\tau)f(\tau)\phi(\tau,t) + \frac{1+\eta(t)}{2}\partial_\tau^2\sigma^2(\tau)f(\tau)\phi(\tau,t). \tag{S114}$$

This expression for the marginal probability distribution is a version of the celebrated Crow-Kimura selection-mutation equation for "infinite alleles" in population genetics [1, 5]. From here on, we refer eq. (S114) as the *generalized Crow-Kimura* equation (GCK). The first term of this equation, $\phi(\tau,t)\left(f(\tau,t) - \bar{f}\right)$, is a replicator-like term for continuous phenotypes. The replicator dynamics captures the essence of selection. If a phenotype τ has fitness which is below the average fitness $\bar{f}$ it will tend to disappear, whereas if it is above $\bar{f}$ it will tend to expand through the population. The second term, that resembles the divergence of a current from a Fokker-Planck eq.([8]), captures the effects of mutation/variation:

$$\partial_t\phi(\tau,t) = \eta(t)\left(f(\tau,t) - \bar{f}\right)\phi(\tau,t) - \partial_\tau J(\tau,t) \tag{S115}$$

$$J(\tau,t) = (1+\eta(t))\theta(\tau)g(\tau)\phi_G(\tau,t) - \left(\frac{1+\eta(t)}{2}\right)\partial_\tau\sigma^2(\tau)g(\tau)\phi_G(\tau,t). \tag{S116}$$

Note that this term has the typical structure of a conserved local probability current of a Fokker-Planck equation, modeling variations as a reaction-diffusion dynamics [5] and then allowing $\phi(\tau,t)$ to move across the phenotype space. Let us note that the main structural difference with the classic Crow-Kimura eq. is the presence of the fitness function in the variation terms. This dependence is typical of phenotypic evolution and couple together ecological and evolutionary timescales.

Consider the two types of variation kernel presented in the main text. The additive case has no direct dependence on the initial phenotype; so we choose :

$$\beta_A(\tau-\tilde{\tau}) = \frac{e^{-\frac{(\tau-\tilde{\tau})^2}{2\alpha_A^2}}}{Z_A}, \qquad Z_A = \sqrt{2}\alpha_A\int_{-\frac{\tilde{\tau}}{\sqrt{2}\alpha_A}}^\infty e^{-y^2/2}dy = \alpha_A\frac{\sqrt{\pi}}{\sqrt{2}}Erfc\left(-\frac{\tilde{\tau}}{\sqrt{2}\alpha_A}\right), \tag{S117}$$

where, due to the truncation in the domain $[-\tilde{\tau},\infty]$, the normalization function Z_A presents a dependence on the phenotypic state and $Erfc$ stands for the complementary error function. Its first two cumulants are:

$$\theta_A(\tau) = \frac{\int_{-\tau}^\infty \delta e^{-\frac{\delta^2}{2\alpha_A^2}}}{Z_A}d\delta = 2\alpha_A e^{-\frac{\tau^2}{2\alpha_A^2}}Erfc\Big(-\frac{\tau}{\sqrt{2}\alpha_A}\Big)^{-1}, \tag{S118}$$

$$\sigma_A^2(\tau) = \alpha_A^2\left[1 - Erfc\left(\frac{\tau}{\sqrt{2}\alpha_A}\right)^{-1} e^{-\frac{\tau^2}{2\alpha_A^2}}\left(\frac{\tau}{\sqrt{2}\alpha_A} - e^{-\frac{\tau^2}{2\alpha^2}}Erfc\left(\frac{\tau}{\sqrt{2}\alpha_A}\right)^{-1}\right)\right]. \tag{S119}$$

For sufficiently small α it is possible to ignore the $Erfc$ function, such that:

$$\theta_A(\tau) \approx 0 \qquad \sigma_A^2(\tau) \approx \alpha_A^2. \tag{S120}$$

On the other hand, is the multiplicative case we consider a Gaussian with the variance proportional to the phenotype:

$$\beta_M(\tau-\tilde{\tau};\tilde{\tau}) = \frac{e^{-\frac{(\tau-\tilde{\tau})^2}{2\alpha_M^2\tilde{\tau}^2}}}{Z_M}, \qquad Z_M = \int_{-\tilde{\tau}}^\infty d\delta e^{-\frac{\delta^2}{2\alpha_M^2\tilde{\tau}^2}} = \sqrt{2}\alpha_M\tilde{\tau}\int_{-\frac{1}{\sqrt{2}\alpha_M}}^\infty e^{-y^2}dy = \alpha_M\tilde{\tau}\sqrt{\frac{\pi}{2}}Erfc\left(-\frac{1}{\sqrt{2}\alpha_M}\right); \tag{S121}$$

whose first two moments are:

$$\theta_M(\tau) = \sqrt{\frac{2}{\pi}}\tau\alpha_M e^{-\frac{1}{2\alpha_M^2}} Erfc\left(-\frac{1}{\sqrt{2}\alpha_M}\right)^{-1},$$
$$\sigma_M^2(\tau) = \alpha_M^2\tau^2\left[1 - Erfc\left(-\frac{1}{\sqrt{2}\alpha_M}\right)^{-1} e^{-\frac{1}{2\alpha_M^2}}\left(\frac{1}{\sqrt{2}\alpha_M} - e^{-\frac{1}{2\alpha_M^2}} Erfc\left(-\frac{1}{\sqrt{2}\alpha_M}\right)^{-1}\right)\right]. \quad \text{(S122)}$$

For sufficiently small α it is possible to approximate them to:

$$\theta_M(\tau) \approx 0, \qquad \sigma_M^2(\tau) \approx \tau^2\alpha_M^2. \quad \text{(S123)}$$

S4. DEVIATION BETWEEN THEORY AND SIMULATIONS

Systematic deviations between theory and simulations have been reported in the main text. Hence, one needs to check if the performed approximations are valid for the range of parameters used in simulations.

A. Validity of the small variation approximation and border effects

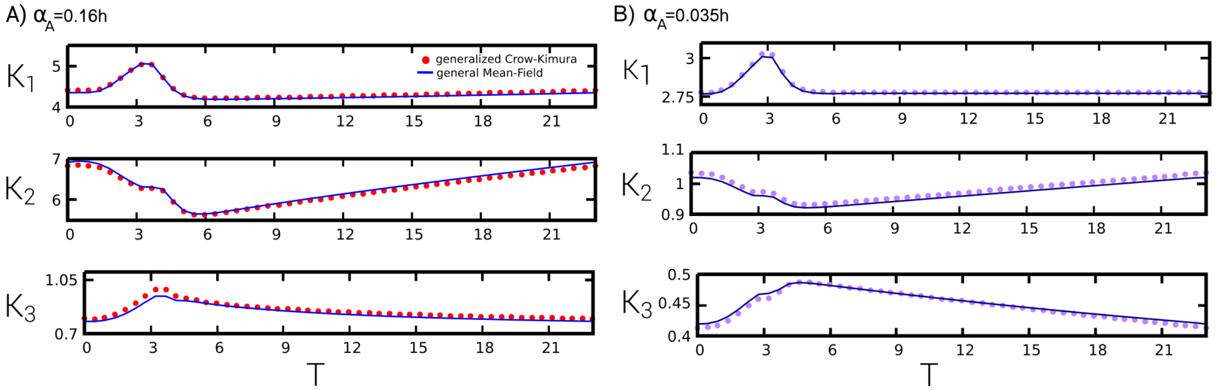

FIG. S1. **Validity of the small variation approximation in the additive case.** Evolution of the first three cumulants (K_1, K_2, K_3) in a asymptotic cycle calculated by the numerical integration of both the general mean-field equation (S95)(solid line) and the generalized Crow-Kimura equation (S114)(points) for two different values of α_A, in **(A)** $\alpha_A = 0.16h$ and in **(B)** $\alpha_A = 0.035h$. The first variation value is the best fit the experimental results (main text), while the second causes negligible border effects. In both cases the small-variation approximation is a good approximation. Parameter values: $T_a = 3h$, the rest of the parameters, as well as the initial conditions, are kept fixed as specified in the main text).

In this section we explore the validity of two different approximations used in the previous section: (i) the small-variation approximation, that leads from the general mean field equation (S95) to the GCK equation (S114), and (ii) the neglection of the border effects in the truncated domain $\delta \in]-\tilde{\tau}, \infty[$, (S120-S123) in equation (S114).

Validity of the small variation approximation. The generalized Crow-Kimura equation (S114) is derived through a diffusion approximation of the full mean-field equation (S95). This approximation is valid only in the limit of small variation, thus here we verify if the optimal values of α_A and α_M, i.e. the ones that best fits to experimental results, meet this condition. At a practical level, the objective of this section is to compare the results given by the general mean-field eq. (S95) and the generalized Crow-Kimura eq. (S111) for the full density both in the additive (S118) and multiplicative(S122) case. In order to quantitatively compare these two equations one can define the parameter δ:

$$\delta^{as.}(\alpha) \equiv \frac{1}{M}\sum_{j=0}^{M}\delta_j^{as.} = \frac{1}{M}\sum_{j=0}^{M}|\langle\tau_j^{G.M.F.}\rangle - \langle\tau_j^{G.C.K.}\rangle| \quad \text{(S124)}$$

where $M+1 = \frac{T_{max}}{\Delta t}+1$ is the number of measures taken through a whole cycle (note that $+1$ is added since measures starts at $t=0$), $T_{max} = 23h$ is the total duration of one single experimental cycle, $\Delta t = 0.5h$ is the interval between

measures, $\langle \tau_j^{G.M.F.} \rangle$ and $\langle \tau_j^{G.C.K.} \rangle$ are the mean of the general mean field and the generalized Crow-Kimura equation respectively at time $j \cdot \Delta t$, and super-index $as.$ in $\delta^{as.}(\alpha)$ points out that comparison is performed in the asymptotic state (see fig. (S4 B) for a visual definition of $\delta^{as.}$).

On the one hand, the numerical integration in the additive amplitude case (S117) is straightforward. Fig. S1 shows the time evolution of the first three cumulants (K_1,K_2 and K_3) of $\phi(t,\tau)$ along a cycle in the asymptotic state both for the general mean field and the GCK equation. Moreover, in fig. S2 the parameter δ is plotted for different variation amplitudes α_A, showing the range of applicability of the small-variation approximation.

On the other hand, the multiplicative case presents some subtleties since the multiplicative variation kernel (S121)

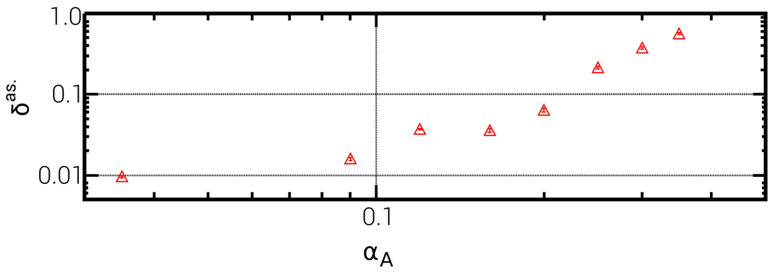

FIG. S2. **Range of validity of the small variation approximation in the additive scenario**. Systematic comparison of equations (S114) and (S95) via parameter (S124) for different α_A values, both axis in $log-$scale. Note that the deviation monotonically increases with α_A. In particular for $\alpha_A = 0.16h$, the one used in the main text results, the deviation $\delta_{st.} = (3.5 \pm 0.2) \cdot 10^{-2}h$, (S124) is small enough to use equation (S114). Parameter values: $T_a = 3h$, the other as in the main text.

has a singularity in $\tau = 0$. In addition, since its variance is proportional to $\alpha \cdot \tilde{\tau}$, one has to numerically impose that:

$$\lim_{\tilde{\tau} \to 0} \beta(\tau - \tilde{\tau}) = \delta(\tau - \tilde{\tau}). \tag{S125}$$

Individuals in the vicinity of $\tilde{\tau} = 0$ experience either negligible or null variations after reproduction, thus one can safely decompose the variation contribution of the general mean field equation (S95) in two terms:

$$2\delta_{\eta,1} b \int_0^\infty d\tilde{\tau} \beta(\tau - \tilde{\tau}) \phi_G(\tilde{\tau}, t) = 2\delta_{\eta,1} b \left(\int_0^\epsilon d\tilde{\tau} \delta(\tau - \tilde{\tau}) \phi_G(\tilde{\tau}, t) + \int_\epsilon^\infty d\tilde{\tau} \beta(\tau - \tilde{\tau}) \phi_G(\tilde{\tau}, t) \right) \tag{S126}$$

where ϵ is a small parameter that delimits the vicinity of $\tilde{\tau} = 0$, such that variations of individuals with $\tilde{\tau} < \epsilon$ can be considered as null. The initial condition $\phi(t = 0, \tau) = \phi_G(t = 0, \tau)$ is defined by cases: it is a truncated Gaussian (see methods in the main text), with the peak $\mu\epsilon$, and standard deviation is $\sigma = 1h$ 16min for $\tau \geq \epsilon$, and it is set to zero for $0 < \tau < \epsilon$. Fig. (S3) shows the integration of the mean-field equation with this numerical prescription and it is compared with the GCK eq. In conclusion, the validity of the small-variation approximation both in the additive and multiplicative case is confirmed.

Border effects. We also study the importance of border effects, i.e the dependence on $\tilde{\tau}$ (initial phenotype) in the normalization/moments of β given by the truncation of the probability density in $]-\tilde{\tau}, \infty[$. Equivalently, we check if their negligence, eqs.(S120,S123), is a good approximation in the range of variation amplitudes α_A and α_M that fits the experimental results. In fig. (S4) the exact moments, (S118-S122), are compared with the approximated ones,(S120,S123), both in the additive and multiplicative case. Clearly, the results indicate the border effects are considerable in the additive case, but negligible in the multiplicative one.

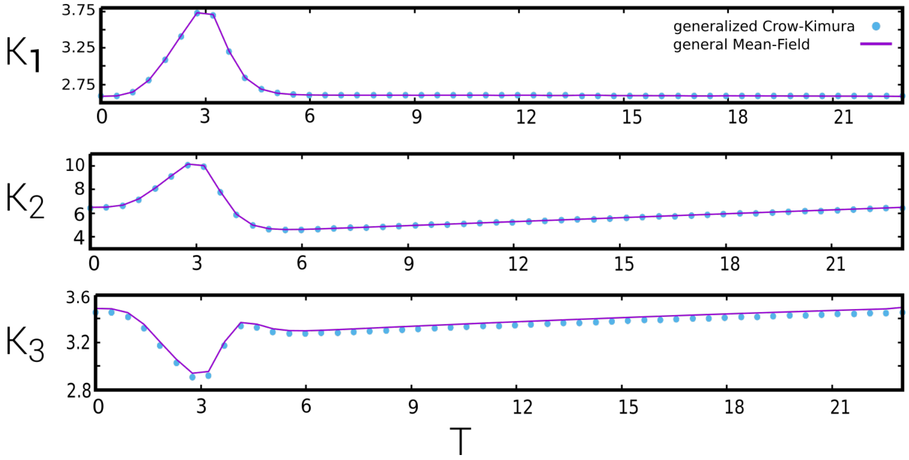

FIG. S3. **Validity of the small variation approximation in the multiplicative scenario**. Evolution of the first three cumulants (K_1, K_2, K_3) in a asymptotic cycle calculated by the numerical integration of both the general mean-field equation (S95)(solid line) and the generalized Crow-Kimura equation (S114)(points). The generalized Crow-Kimura equation is a good approximation of the general mean-field equation for the α_M-value that best fits the experimental results, e.g.$\delta_{st.} = (1.5 \pm 0.3) \cdot 10^{-3} h$. Parameter values: $T_a = 3h, \alpha_M = 0.048$, the remaining are specified in the main text.

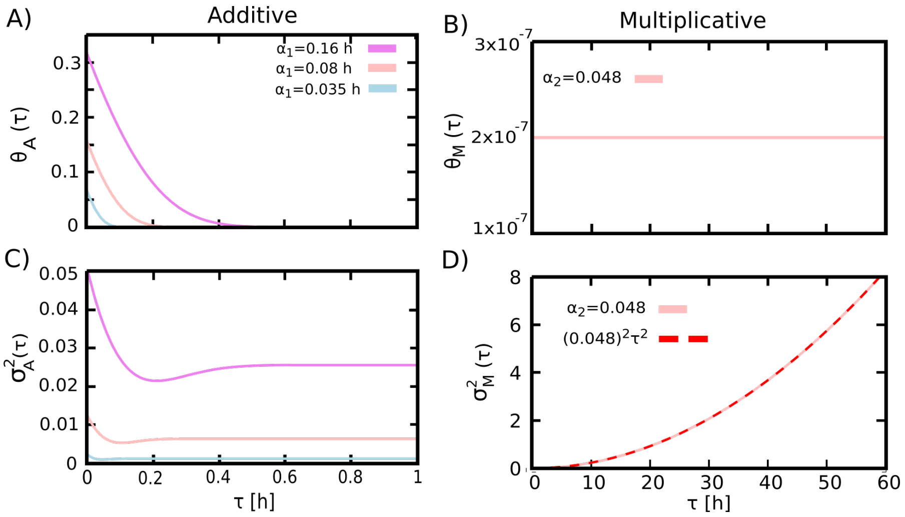

FIG. S4. **Border effects in the variation functions**. First (**A-B**) and second (**C-D**) moment of the variation functions as function of the trait τ in additive (left) and multiplicative (right) variation cases. In **(A)** the additive case, $\theta_A(\tau)$ is positive in $\tau = 0$ but decays rapidly to zero as τ increases, such that it is sufficient to restrict τ axis between 0 and 1. In **(C)** the same is shown for the second moment $\sigma_A^2(\tau)$. Note that the magnitude of the dependence decreases with α_A). Nevertheless, the value used in the main text, $\alpha_A = 0.16h$, is too large to neglect (S120) this effect in the generalized Crow-Kimura eq. On the other hand, in the multiplicative case the main text value, $\alpha_M = 0.048$, is small enough to avoid the border effects in the GCK eq. In **(B)** one can observe that $\theta_A(\tau)$ almost vanishes, and in **(C)** that the exact moment $\sigma_M^2(\tau)$ coincides quite well with the approximation $\alpha_M \tau^2$(S123).

B. Finite-size effects

Here we complement the analysis of the finite-size effects of the main text with the number of growing and dormant individuals as a function of time during a asymptotic cycle (fig. *S6*). In addition, the definition of the parameter $\delta^{st.}(T_a)$ (see fig. S4 B), which os analogous to eq.(S124), to measure the difference between the macroscopic theory and the simulations is:

$$\delta^{as.}(T_a) \equiv \frac{1}{M} \sum_{j=0}^{M} \delta_j^{as.} = \frac{1}{M} \sum_{j=0}^{M} |\langle \tau_j^{sim.} \rangle - \langle \tau_j^{theo.} \rangle|. \tag{S127}$$

where $M+1 = \frac{T_{max}}{\Delta t}+1$ is the number of measures taken through a whole cycle (note that +1 is added since measures starts at $t=0$), $T_{max}=23h$ is the total duration of one single experimental cycle, $\Delta t = 0.5h$ is the waiting interval between measures, $\langle \tau_j^{sim.} \rangle$ and $\langle \tau_j^{theo.} \rangle$ are the mean lag time calculated from simulations and the generalized Crow-Kimura equation at time $j \cdot \Delta t$, respectively and the super-index as indicates that it is calculated in the asymptotic state.

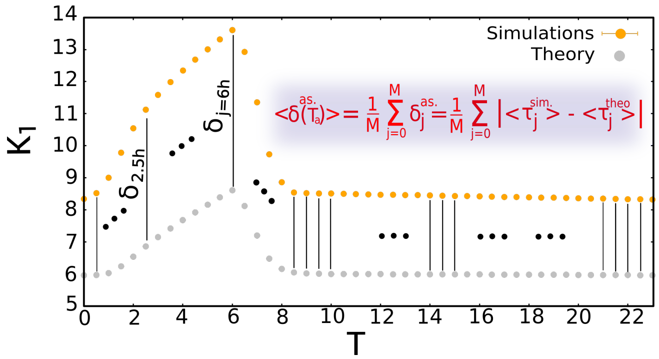

FIG. S5. **Schematic definition of the Parameter** δ (illustrated in the multiplicative amplitude scenario). $\alpha_M = 0.048, T_a = 6h$

S5. SPONTANEOUS SHIFTING TO THE DORMANT STATE

In this section we investigate how the main results are changed when there is a non-vanishing spontaneous rate to enter the dormant state in the absence of antibiotics, e.g. when starving. Let us call such a generalized rate $S(\tau_i, \boldsymbol{\tau}_N)$; in the main text we use

$$S(\tau_i, \boldsymbol{\tau}_N) = \begin{cases} s_k = s & \text{if} \quad 0 \le T \le T_a \\ s_f = 0 & \text{if} \quad T_a < T \le T_{max} \end{cases} \tag{S128}$$

where the sub-indexes k and f denote “killing medium” and “fresh medium”, respectively.

Given that bacteria can enter in dormant state when they sense lack of resources, i.e. when they approach starvation (i.e. the system carrying capacity), we consider a small, but non-zero, value for the rate to enter the dormant state in the fresh medium. In particular, we keep s_k constant as in the main text but consider two possible scenarios for s_f:

(i) a constant rate $s_f = \tilde{s} = 0.01$ all across the fresh-medium phase, or

(ii) a state-dependent rate, increasing as the carrying capacity is approached; in particular we consider a sigmoidal-like function $s_f = \tilde{s}\,(\tanh[-cN_G/K])$, ($\tilde{s} = 0.01$).

Computational results for the variants of the model obtained by implementing these types of rate are displayed in Figures S6 and S7, corresponding to the end of the $10th$ exposure cycle (results are presented only for the multiplicative version of the model, but similar curves and conclusions can be obtained for the additive version).

Observe that, on the one hand, a non-vanishing s_f increases the values of K_1, K_2, K_3, but maintains the functional form of the lag-time distributions, see S6 (b) and (c). Thus, one can recover the results of the main text by just tuning the mutational amplitude parameter, α_M. On the other hand, the dynamics of the total number of bacteria in both growing, N_G, and dormant state, N_D, along the cycles changes significantly as shown in Fig. S7. In particular, there is a larger number of bacteria in the dormant state —with respect to the scenario in the main text, with $s_f = 0$— when the killing phase starts, as bacteria enter the dormant state during the fresh medium of the previous cycle. Indeed, N_D decreases until a minimum is reached, as bacteria die in presence of the antibiotics. N_G maintains a very similar dynamics, but there is an overall reduction as the number of bacteria approaches the maximum carrying capacity. A similar behavior is observed in Fig.S6. In conclusion, considering $s_f = 0$ —though not a completely realistic assumption— is a reasonable approximation which eases the tractability of the model by reducing the computational times without significantly modifying the qualitative results.

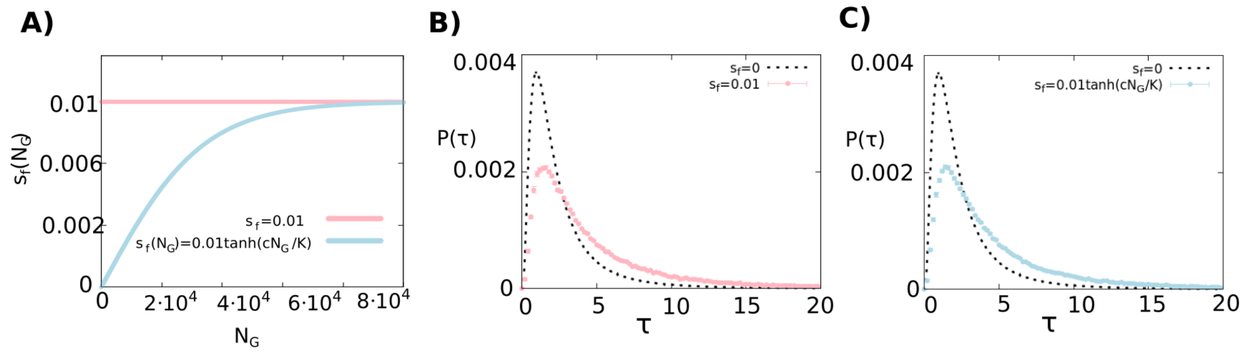

FIG. S6. **A)** Functional dependence of the rate to enter the dormant state in fresh medium s_f and the number of particles in growing state N_G. **B)** and **C)** Lag-time probability distribution function, $P(\tau)$, at the end of the tenth cycle (for the multiplicative case) for a constant s_f and $s_f = s_k\,(\tanh[-cN_G/K])$, respectively. The results in the main text, for $s_f = 0$, are represented by a dashed line. Observe that in both cases the value of K_1, K_2, K_3 increase, but the qualitative form of the distributions remains unchanged. Parameter values: $h_\tau = 0.01$, $\alpha_M = 0.048$, $T_a = 3h$, $c = 3$, $T = 23h$, the rest of parameters are fixed as in the main text.

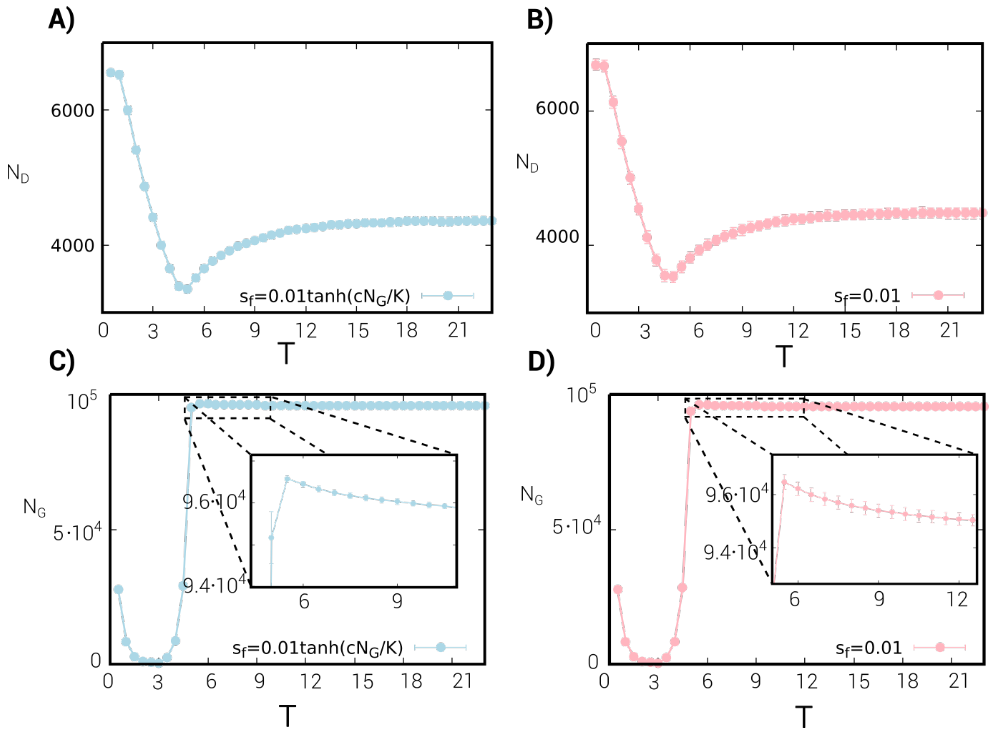

FIG. S7. **Dynamics of the averaged population structure within one cycle**. Abundances N_G and N_D along the 10th cycle for s_f constant —**A)** and **C)**— and $s_f = \tilde{s}\,(\tanh[-cN_G/K])$ —**B)** and **D)**—. The two scenarios are very similar to each other. At the beginning of the cycle N_D is non-vanishing, since bacteria entered this state during the fresh phase of the previous cycle. During the antibiotic exposure phase both, N_D and N_G, decrease to a minimum as the bacteria die. When the antibiotic is removed and a fresh medium is added, N_G grows towards the system's carrying capacity. Observe that the bacteria still enter the dormant state, but the reproduction rate is much higher, in such a way that an overall reduction of N_G is only observed as the system approaches the carrying capacity.

S6. ADDITIONAL FIGURES

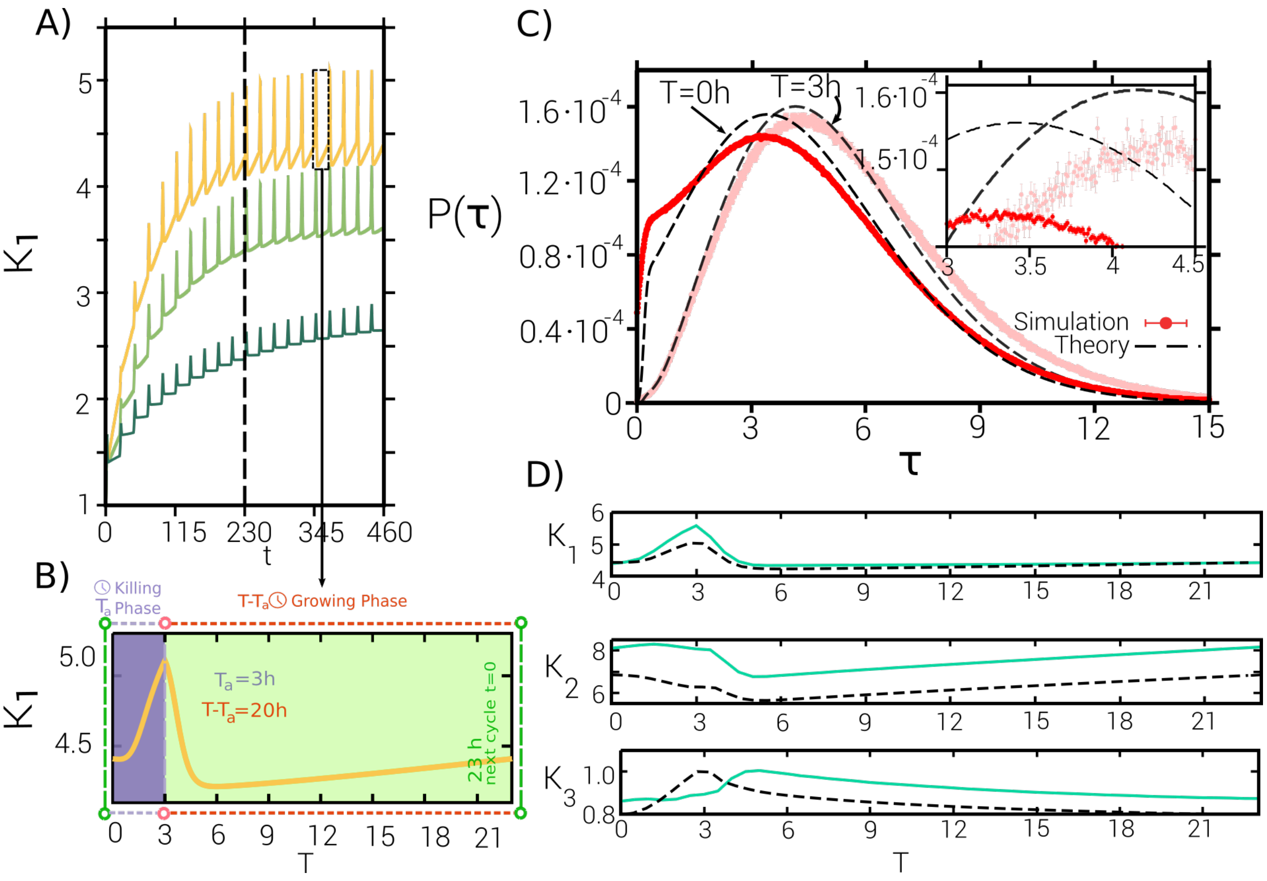

FIG. S8. **Characterization of the asymptotic state in the additive version of the model.** **(A)** Relaxation of the mean lag-time to its asymptotic state (curves obtained from the integration of Equation (S114) with $T_a = 3h$ and additive model. The different curves correspond to three different values of the variation amplitude, α_A; from the lowest to the highest: $\alpha_A = 0.035h$, $\alpha_A = 0.1h$ and $\alpha_A = 0.16h$. **(B)** Zoom of the curve $\alpha_A = 0.16h$ for one single cycle. In particular, the mean lag time, K_1, is shown along a cycle in the asymptotic regime. At $T = 0$ the antibiotic is added and the system enters in the "killing phase" ($T \in [0, T_a]$). When the antibiotic is present, the system experiences a selection pressure towards longer lag times, in consequence, K_1 increases. At $T = T_a$ the antibiotic is washed and the fresh medium is added ($T \in [T_a, T_{max}]$). In this regime, the selection pressure is towards shorter lag times and K_1 relaxes back to the initial value. **(C)** Lag-time probability distribution at $T = 0$ (leftmost curve) and $T = T_a = 3h$ (rightmost curve) as derived theoretically (Eq.(S114), dashed lines) and computationally (dots). In the asymptotic state the system oscillates between these two limiting probability distributions, both of them exhibiting weak tails. **(D)** Evolution of the first three cumulants, K_1, K_2 and K_3, (mean, variance , and skewness respectively) along a cycle in the asymptotic state (both theoretical and computational results are displayed). Observe that in C/D the theory correctly predicts the properties of the distribution, however there are deviations due to finite size effects.

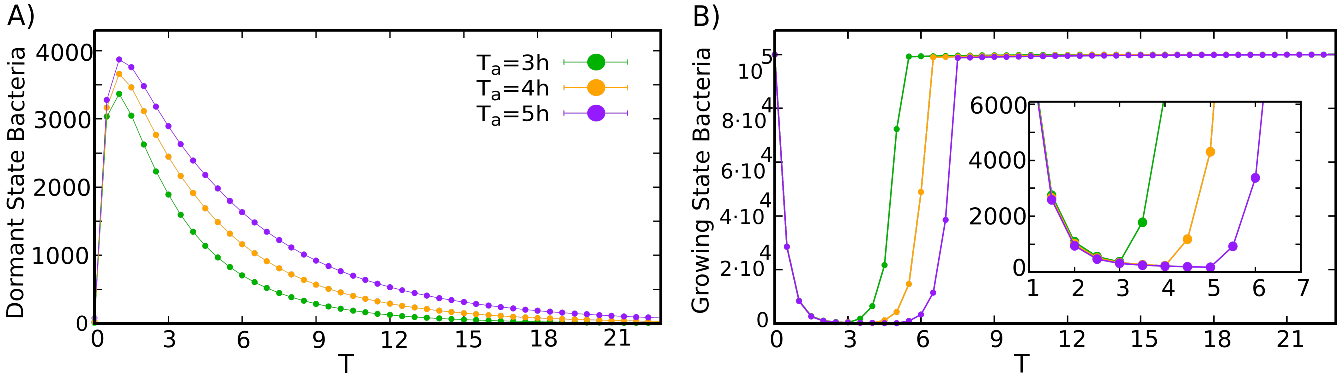

FIG. S9. **Simulated number of cells via the Gillespie algorithm** —additive-amplitude scenario, linear scale—. **a)** Number of cell in dormant state during a whole cycle in the asymptotic state for different exposition times T_a. **b)** Same as a) but for growing state bacteria. Obviously, the minimum number of bacteria in growing state $N_{G,min}$ is reached at $t = T_a$, since, during the antibiotic exposure phase, that number can only decrease. Parameters: $\alpha_A = 0.16h$, the rest are fixed as in the main text.

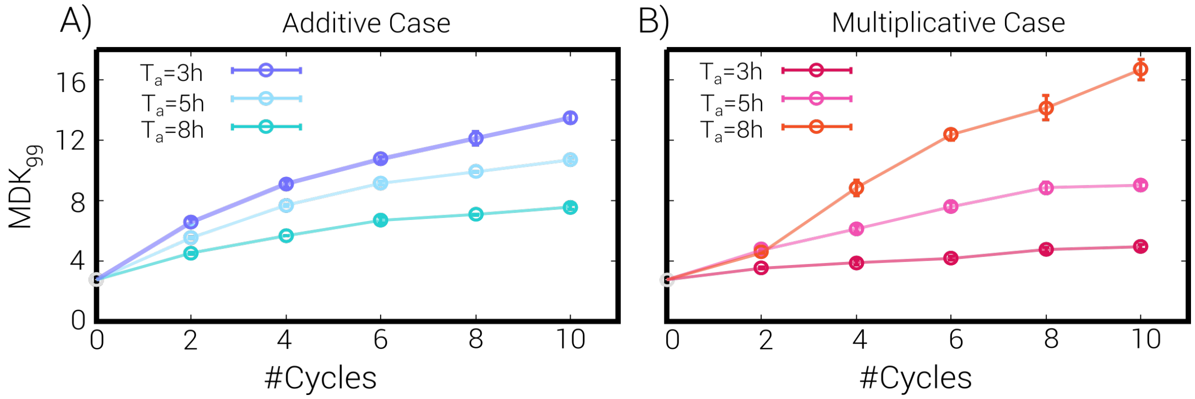

FIG. S10. **Evolution of the MDK_{99} over 10 exposure cycles**. **a)** Additive case. **b)** Multiplicative case. In our simulations, the MDK_{99} is calculated analogously to the experimental procedure. After a certain number cycles (i.e. # in the figure) of antibiotics-fresh environment, the evolved population is posed back in the antibiotic phase for a long time. The maximum number of cycles is 10 as in the experiments. The MDK_{99} is estimated by the time necessary to kill to the 99% of the population. Both in the additive and multiplicative case, the MDK_{99} increases with the # and T_a. Interestingly, in the multiplicative case by increasing T_a the change in MDK_{99} is bigger than the additive one.

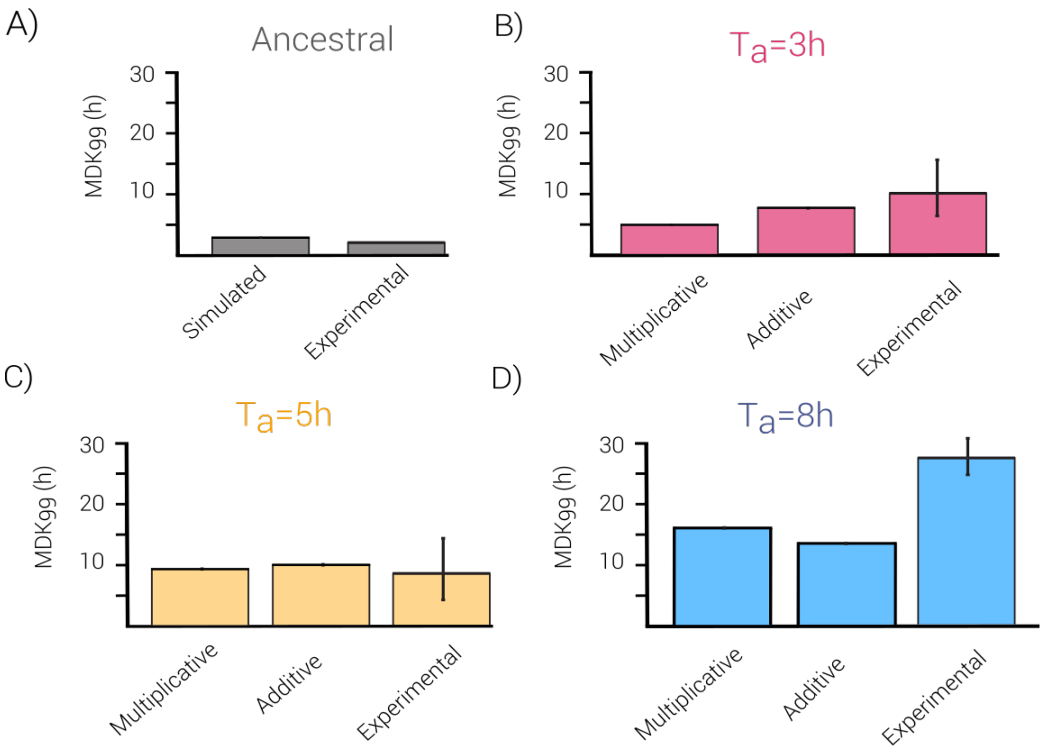

FIG. S11. **Comparison of experimental and simulated MDK_{99} of the evolved population after 10 cycles of exposure**. For both the additive and multiplicative cases, we observe that the simulated MDK_{99} falls within —or it is very close— the experimental values (i.e. the mean plus error) for $T_a = 3h$ and $5h$, but outside for the case of $T_a = 8h$. This result is to be expected given the higher noise of the experimental measurements. In particular the experimental mean is $\langle\tau\rangle^{exp.}_{T_a=8h} = 10 \pm 1h$ higher than the theoretical prediction of $8h$.

S7. MOVIES

We provide three videos to help visualize the system dynamics:

- (V1) https://github.com/MCMateu/Phenotypic-dependent-variability/blob/main/AsymptoticAdditive.mp4
- (V2) https://github.com/MCMateu/Phenotypic-dependent-variability/blob/main/AsymptoticMultiplicative.mp4
- (V3) https://github.com/MCMateu/Phenotypic-dependent-variability/blob/main/TransitoryAdditive.mp4

The first two videos show the evolution of $\phi(\tau, t)$ along an antibiotic exposure cycle in the asymptotic state, calculated both by the simulation via Gillespie algorithm and by numerical integration of generalized Crow-Kimura equation (S111) in the additive (V1) and multiplicative (V2) case respectively. Note that, as in fig. 4 of the main text, there exists a small deviation between theory and simulation, due to finite-size effects. The duration of the killing phase is $T_a = 3h$, and the duration of the whole cycle is $T = 23h$. The full length of the video is 23 s, thus each second approximately matches $1h$ of the cycle. During the first three seconds the distributions move towards longer lag times (to the right side), since individuals with longer lag times are the one that survives, until the mean lag time reaches a maximum value when antibiotics are removed. Then, the distribution moves back towards the initial configuration. Numerical value the of parameters: $\alpha_A = 0.16h$, $\alpha_M = 0.048$, $b = 2.4h^{-1}$, $s = 0.12h^{-1}$, $d = 3.6 \cdot 10^{-5}h^{-1}$.

On the other hand, the third video (V3) shows the transient dynamics of $\phi(\tau, t)$ as calculated by the numerical integration of the general mean-field equation (S95) and the generalized Crow-Kimura (S111) in the additive case. The full length of the video is $50s$ at a speed of $25fps$, the full video counts with 1250 frames. Each cycle has 50 frames, so the total duration of each cycle is approximately $2s$. The duration of the killing phase is $T_a = 5h$. The oscillations observed in the video correspond with the killing phase, when the density moves to the right, and with the growing phase, when it moves to the left. Note that the two distributions matches almost perfectly. Numerical value the of parameters: $\alpha_A = 0.035h$, $b = 2.4h^{-1}$, $s = 0.12h^{-1}$, $d = 3.6 \cdot 10^{-5}h^{-1}$.

REFERENCES

[1] James F Crow, Motoo Kimura, et al. "An introduction to population genetics theory." In: *An introduction to population genetics theory.* (1970).

[2] Erwin Frey, Johannes Knebel, and Peter Pickl. *Mean-field equation for a stochastic many-particle model of quorum-sensing microbial populations.* 2018. arXiv: 1802.05307 [math-ph].

[3] Daniel T Gillespie. "A general method for numerically simulating the stochastic time evolution of coupled chemical reactions". In: *Journal of computational physics* 22.4 (1976), pp. 403–434.

[4] Subhadip Raychaudhuri. "Kinetic Monte Carlo simulation in biophysics and systems biology". In: *Theory and applications of Monte Carlo simulations* (2013).

[5] Matteo Sireci and Miguel Angel Muñoz. "Statistical physics of phenotypic evolution: Adaptive dynamics and beyond". unpublished.

[6] Herbert Spohn. *Large Scale Dynamics of Interacting Particles.* Springer, 1991.

[7] Andrew Charteris Tadrowski. "Role of phenotype switching during biological evolution in static environments". In: (2017).

[8] Nicolaas Godfried Van Kampen. *Stochastic processes in physics and chemistry.* Vol. 1. Elsevier, 1992.
